# Supplementary material for: A mechanochromic donor-acceptor torsional spring
Source: Nat Commun. 2021 Jul 9;12:4243. doi: 10.1038/s41467-021-24501-1 (PMC8270966; doi:10.1038/s41467-021-24501-1)
Supplement: Supplementary file 1 — Supplementary Information [file 41467_2021_24501_MOESM1_ESM.pdf]

Supplementary Information for

A Mechanochromic Donor-Acceptor Torsional Spring

Maximilian Raisch[1], Wafa Maftuhin[2,3], Michael Walter[2,3,4,*] and Michael Sommer[1,*]

[1] Institut für Chemie, Technische Universität Chemnitz, Straße der Nationen 62, 09111 Chemnitz, Germany

[2] FIT Freiburg Centre for Interactive Materials and Bioinspired Technologies, University of Freiburg, Georges-Köhler-Allee 105, 79110 Freiburg, Germany

[3] Cluster of Excellence livMatS @ FIT

[4] Fraunhofer IWM, MikroTribologie Centrum µTC, Wöhlerstrasse 11, 79108 Freiburg, Germany

Table of contents

1. General information 2
2. Theoretical Investigation 3
2.1 Contributions to the optical shifts 7
2.2 Investigation of rotamers 14
2.3 Comparison of different substituents: methyl vs. methoxybutyl 18
3. Mechanochromic response in polydimethylsiloxane (PDMS) 20
4. Synthesis 22
5. Thermal analysis 35
6. Experimental investigation of mechanochromic response in polyphenylene 36
7. NMR Spectra 41
8. Supplementary references 70

1. General information

Chemicals. All chemicals, reagents and solvents were purchased from commercial sources and used as received unless otherwise noted. Solvents used for column chromatography were distilled under reduced pressure before use. *tert*-amyl alcohol, dimethylformamide (DMF), and dimethylacetamide (DMAc) were dried one week over molecular sieve (4 Å).

NMR Spectroscopy. NMR spectra were recorded on an Avance NEO 600 FT spectrometer (^{1}H: 600 MHz, ^{13}C: 151 MHz). The spectra were referenced to the residual solvent peak ($CHCl_3$: δ(^{1}H) = 7.26 ppm, δ(^{13}C) = 77.16 ppm; DMSO: δ(^{1}H) = 2.50 ppm, δ(^{13}C) = 39.99 ppm) and analyzed using Mestrelab Mestrenova v11.0.4. Chemical shifts are given in [ppm], coupling constants in [Hz]. Peak assignments are supported by HSQC and HMBC experiments.

Size Exclusion Chromatography (SEC). Molecular weights were measured on a Shimadzu system comprising a 5 µm precolumn and three SDplus columns with pore sizes ranging from 10^2 to 10^4 Å (Polymer Standards), connected in series with a RID20A RI detector and a SPD-20AV photodiode array UV-vis detector (Shimadzu). Calibration was done with polystyrene standards. THF was used as eluent at 40 °C with a flow rate of 1.0 mL min^{-1}.

High resolution mass spectra (HRMS) were obtained from a Thermo Fisher Scientific Q-Exactive (Orbitrap) instrument in ESI+ mode. Only compound **7** was measured using Bruker Trapped Ion Mobility Spectrometry (tims) time-of-flight (TOF) mass spectrometer in ESI+ mode.

UV-vis Spectroscopy. UV-vis absorption spectra in solution were recorded at 25 °C on a Cary 60 UV-Vis (Agilent Technologies). Thin-film absorption spectra were measured on a Flame-S UV-Vis spectrometer from Ocean Optics, controlled by the OceanView 1.5.2 software. All UV-vis spectra were measured in transmission. Thin-film absorption spectra were smoothed via Savitzky-Golay filter considering 51 pts (**6a**) and 21 pts (**6b**).

Photoluminescence Spectroscopy. PL spectra in solution were recorded on a Horiba Fluoromax 4 at 25 °C using a xenon flash lamp and a Czerny Turner monochromator. Excitation for thin-film emission spectra was carried out using a UV LED (Nichia NVSU233A UV SMD-LED, 365 nm, max. 1030 mW) operated with 900 mA at a distance of 10 cm. PL spectra were measured on a Flame-S UV-Vis spectrometer (see above). Emission spectra were smoothed via Savitzky-Golay filter considering 11 pts (solution) and 21 pts (thin film).

Differential Scanning Calorimetry. DSC measurements were carried out on a DSC 2500 (TA Instruments) in aluminum standard pans under nitrogen atmosphere. Sample masses were between 2.5 and 4.5 mg and the heating-/cooling rate was 10 K min^{-1}.

Thermogravimetric Analysis. TGA measurements were performed on a Mettler-Toledo TGA/DSC 3+ with HT/1600/557-DTA sensor in nitrogen atmosphere. The heating rate was 10 K min^{-1}.

Tensile Testing. Stress-strain experiments were carried out on a Linkam TST-350 using a displacement ramp of 5 mm min^{-1}. A standard specimen shape according to DIN 53504 type 3 was used. The shown values of stress were calculated by considering the constant cross-section area of the specimen's mid-point at 0% strain.

2. Theoretical Investigation

We modeled the structures within Density functional theory (DFT) as implemented in the GPAW package.[1,2] The smooth Kohn-Sham wave functions were represented on real space grids, with grids spacing of 0.15 Å and the electronic density on grids of 0.075 Å spacing. The grid was ensured to cover at least 4 Å around each atom. The relaxation of the structures is considered converged if no force exceed 0.01 eV/Å. The exchange correlation potential is modeled in the generalized gradient correction modeled as devised by Perdew, Burke and Ernzerhof (PBE).[3] Optical properties are calculated by time dependent DFT (TDDFT) in Casidas linear response formalism.[4,5] The number of unoccupied orbitals considered are chosen to be the same as the number of occupied orbitals. All transitions between Kohn-Sham orbitals within an energy range of 8 eV are considered in the linear response calculations, which is checked for convergence for excitations in the optical range (< 3 eV) of interest here.

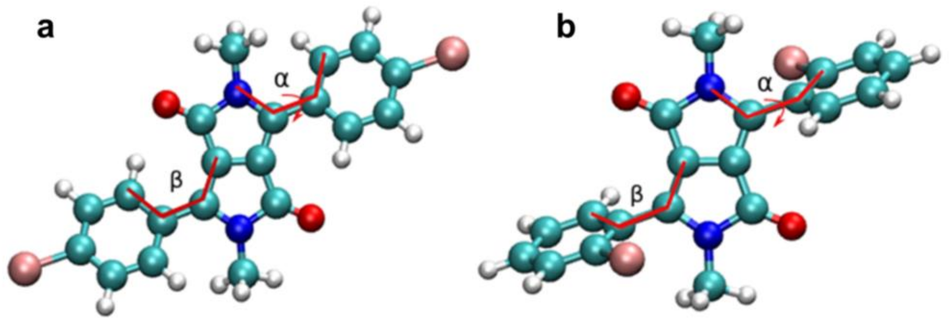

Supplementary Fig. 1. Relaxed structures of **a** *p*-BrPhDPP and **b** *o*-BrPhDPP.

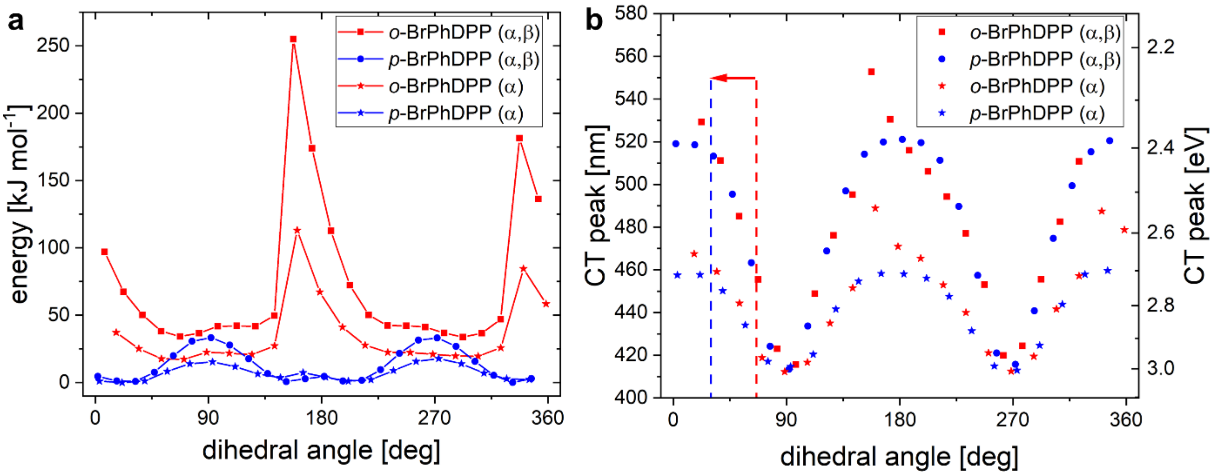

Supplementary Fig. 2. a Torsion potentials for the rotation of one (α) and two (α, β) BrPh groups. **b** The corresponding variation of the CT peak position with the dihedral angle (dashed lines for equilibrium geometries, arrow for direction with external force applied).

Supplementary Fig. 1 shows our relaxed structures of *p*-BrPhDPP and *o*-BrPhDPP. We varied two different rotations of the BrPh ring at the DPP: i) rotations of both BrPh rings (α and β), as we already discussed in the main text (Fig. 2), and ii) rotation of only one BrPh ring (α). Both kinds of rotation started from equilibrium geometry with 30° and 67° for *p*-BrPhDPP and *o*-BrPhDPP, respectively. In Supplementary Fig. 2a we see that for both cases the rotational barrier of *o*-BrPhDPP is larger than in *p*-BrPhDPP due to the strong sterical hindrance between Br and Me. As expected, rotation of one-ring (α) shows about half of the energy barrier compared to the rotation of two rings (α, β). Similarly, a change of both dihedral angles (α, β) is shown to have a higher impact on the CT peak than the rotation of a single ring (α) (Supplementary Fig. 2b).

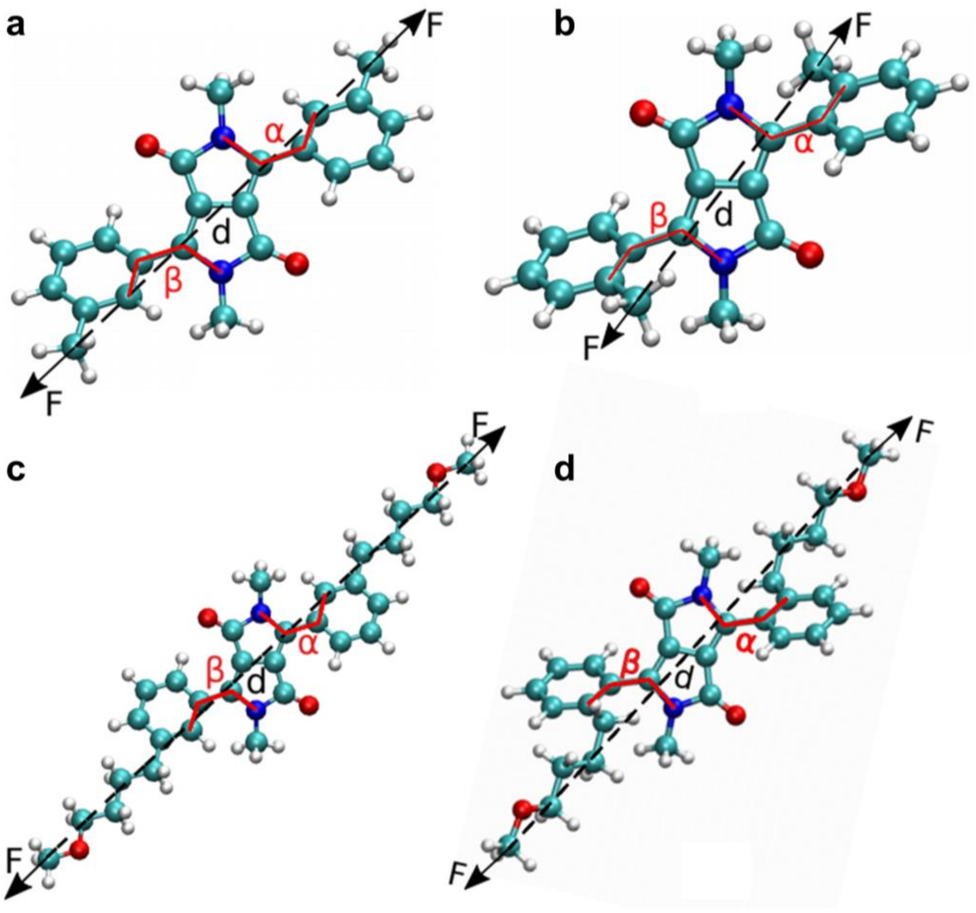

Supplementary Fig. 3. Structures of **a** *m*-MeDPP **b** *o*-MeDPP **c** *m*-MeOBuDPP **d** *o*-MeOBuDPP together with the corresponding distance vector d between the pulling points defining the direction of the external force *F*.

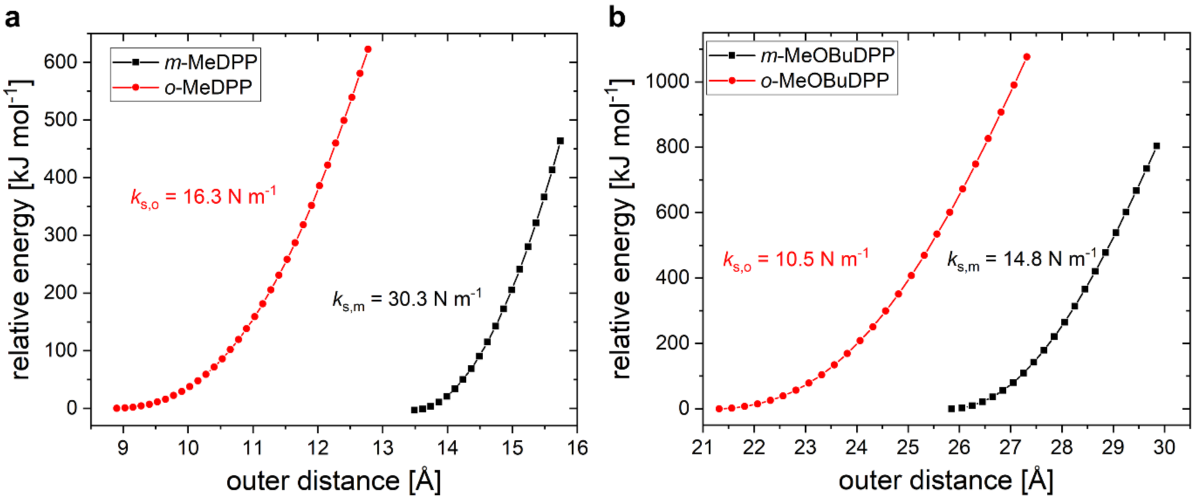

Supplementary Fig. 4. Energy vs outer distance data obtained through COGEF modelling of **a** MeDPP and **b** MeOBuDPP with the corresponding effective spring constants $k_{s,o}$ and $k_{s.m}$.

The modeling of the influence of the external force on the structures was conducted using the constrained geometries to simulated external force (COGEF) strategy.[6–8] There the energy path depends on the outer distance d between the two atoms in the structure where the external force is thought to act on (see Supplementary Fig. 3). The potential $U(d)$ is obtained via the relaxation of all other degrees of freedom. The parameter d is then increased stepwise. The force can be determined either from the derivative $\delta U / \delta d$ or equivalently from the forces acting on the constrained atoms. The resulting COGEF energies are depicted in Supplementary Fig. 4 and show very spring-like behavior, which allowed assigning an effective spring constant to each structure.

The dihedral angle N-C-C-C between DPP core and Ph ring (Supplementary Fig. 3) has a severe effect on its CT peak due to effectiveness of the π-orbital overlap (see main text Fig. 2). Figure 3b shows the change in dihedral angles of *o*-/*m*-MeOBuDPP with increasing external forces. The dihedral angles α and β are practically symmetric independent of the external force. The angles of *m*-MeOBuDPP and *o*-MeOBuDPP change from 30° to 12° and 58° to 21°, respectively, with an external force. The *ortho*-derivative shows a larger shift of dihedral angle compared to *meta*-configuration resulting in a higher mechanochromic response.

2.1 Contributions to the optical shifts

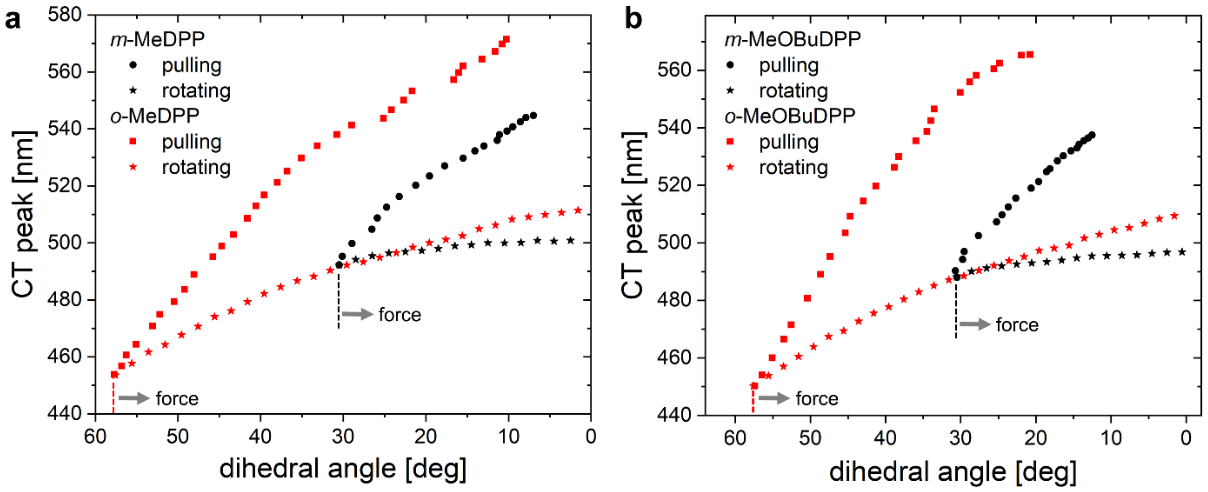

Supplementary Fig. 5. Effect of deformation to the optical properties of **a** MeDPP and **b** MeOBuDPP. The star symbols represent the constrained rotation and square and circle represent stress to the *ortho*- and *meta*-DPP, respectively.

The CT peak shift is not only due to changes in the dihedral angle, but also to other deformations of the DPP molecules. The effect of the deformation itself can be seen from the CT peak difference between pulling (COGEF) and constrained rotation (rotating both Ph rings symmetrically) depicted in Fig 3c of the main text and Supplementary Fig. 5. The significant gap between the two curves shows that deformation also plays an important role for the shift of the optical spectra. The CT peak of rotation of *o*-DPP at ~30° has the same wavelength as the relaxed form of *m*-DPP (zero force). This shows the clear effect of the dihedral angle on the optical spectra. Constrained rotation of *o*-DPP starts to deviate from constrained rotation of *m*-DPP below dihedral angles of 20°. The reason is the unavoidable deformation due to repulsion between the Me-group of the Ph ring and the N-Me-group at the DPP core.

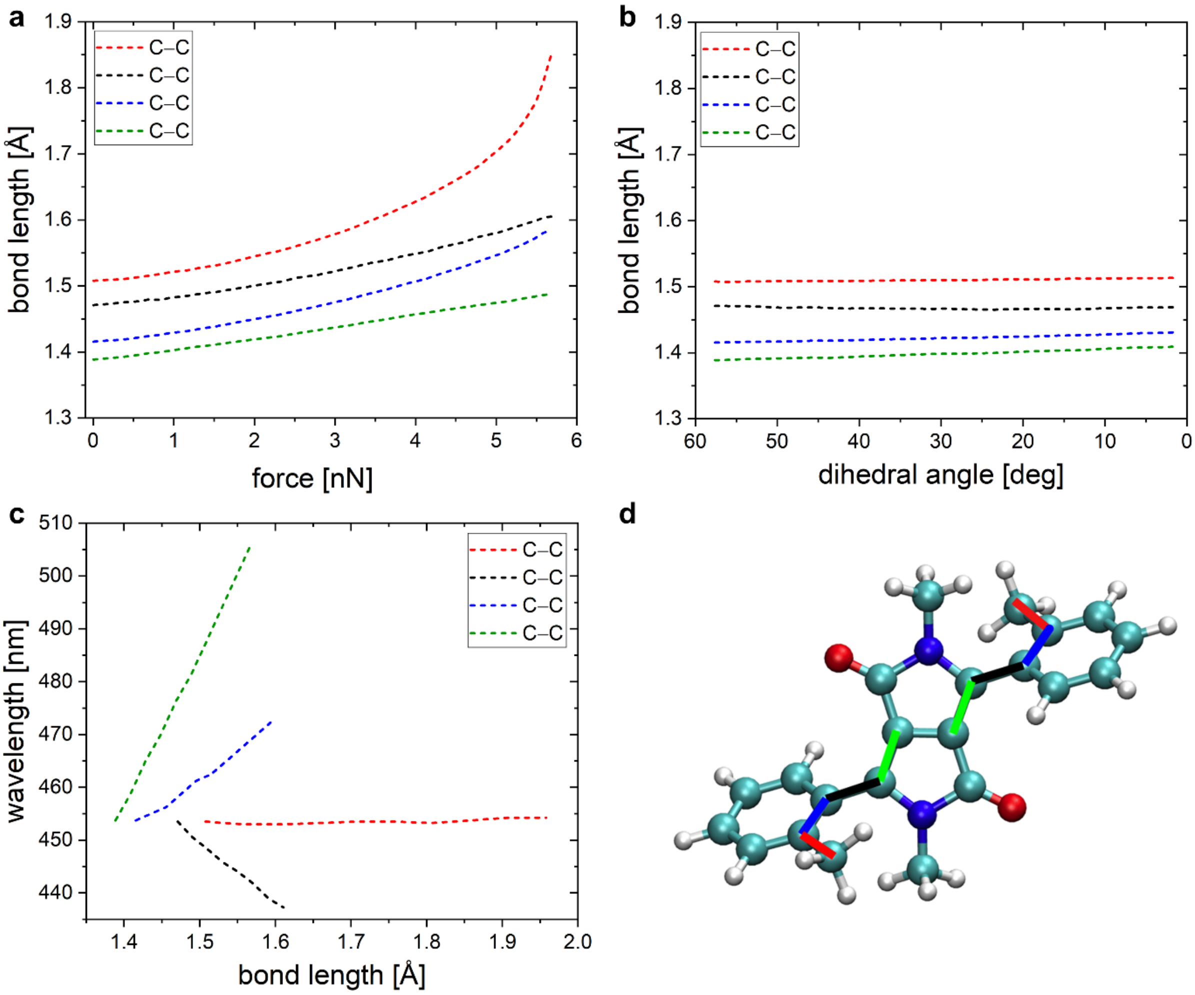

Supplementary Fig. 6. a Deformations due to elongated C-C bonds, **b** influence of constrained rotation on C-C bond lengths and **c** effects of elongated C-C bonds on the optical spectra. **d** Structure of *o*-MeDPP with C-C bond colors as in **a**-**c**.

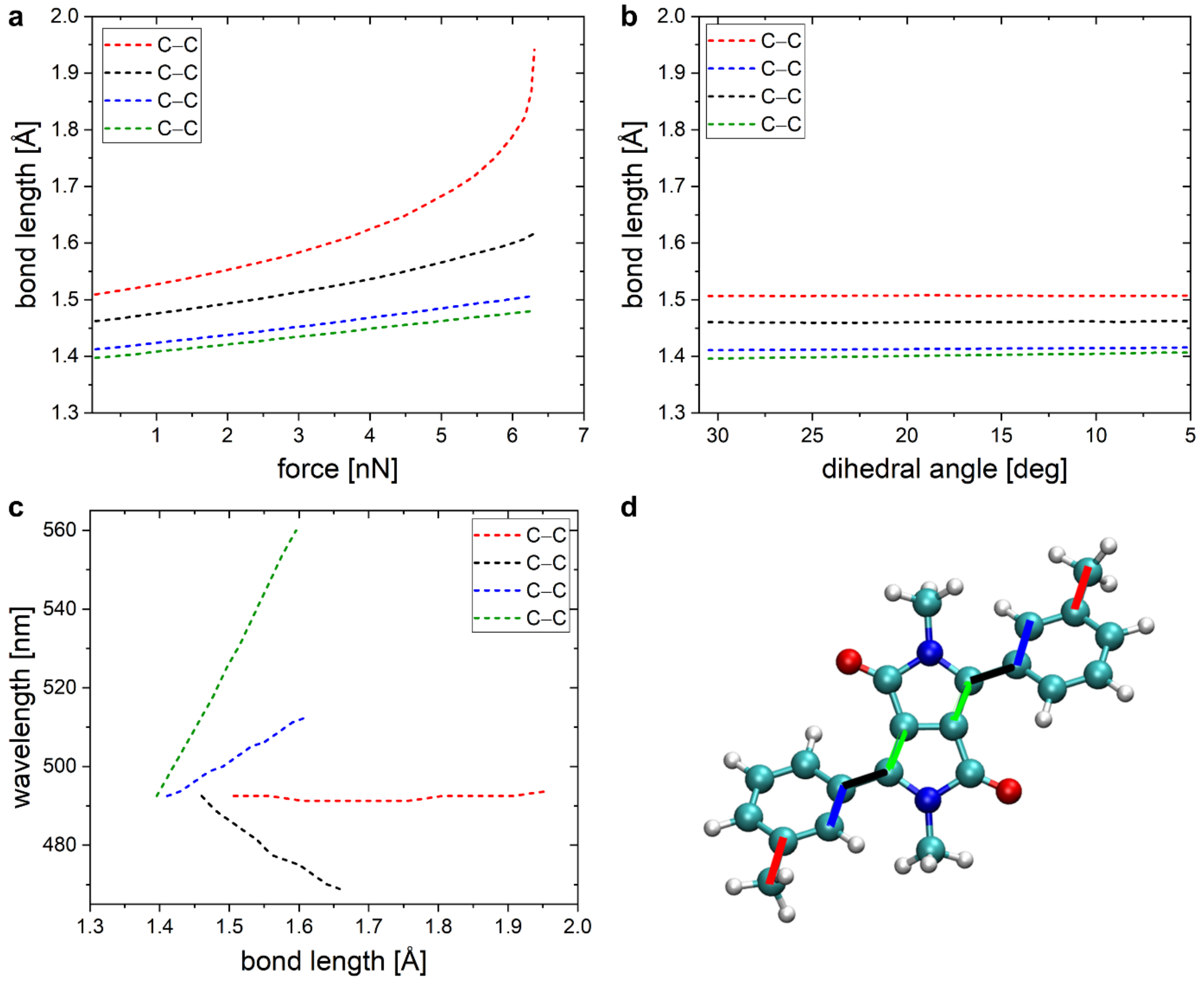

Supplementary Fig. 7. **a** Deformations due to elongated C-C bonds, **b** influence of constrained rotation on C-C bond lengths and **c** effects of elongated C-C bonds on the optical spectra. **d** Structure of *m*-MeDPP with C-C bond colors as in **a**-**c**.

We have further analyzed the deformations in detail in what follows. One of the deformations we observed was the elongation of C-C bonds as seen in Supplementary Figs. 6 and 7 for *o*-MeDPP and *m*-MeDPP, respectively, The C-C bond between outer carbon atom and the Ph ring (red) is the most affected as the force is directly acting at this position, but other C-C bonds are elongated too. As expected, Supplementary Figs. 6b and 7b show that pure rotation does not change these bonds at all. We evaluate the effect of the individual bonds variation on the optical spectra with C-C bond distance in Supplementary Figs. 6c and 7c. COGEF calculations are used here to vary the individual C-C bond length while allowing all other degrees of freedom to relax. While the outer C-C bond length (red) does not affect the CT frequency, changing bond lengths within the Ph ring or the DPP core leads to clear shifts. Elongation of the bonds

marked in green and blue leads to an increase in the wavelength, while elongation of the bond length of the bonds marked in black decreases the wavelength.

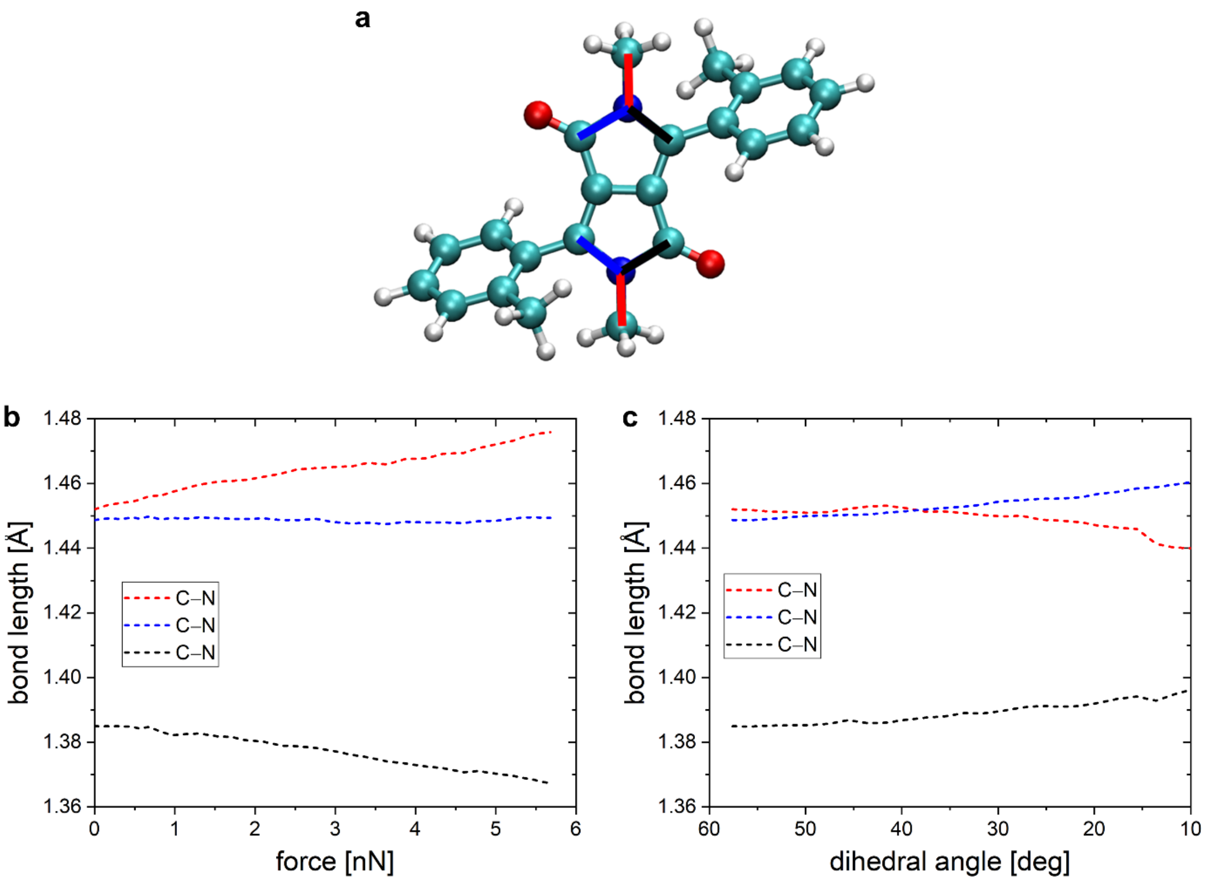

Supplementary Fig. 8 **a** Structure of *o*-MeDPP with C-N bond color code used in **b** and **c**. Changes in C-N bond lengths due to **b** the outer force and **c** constrained rotation.

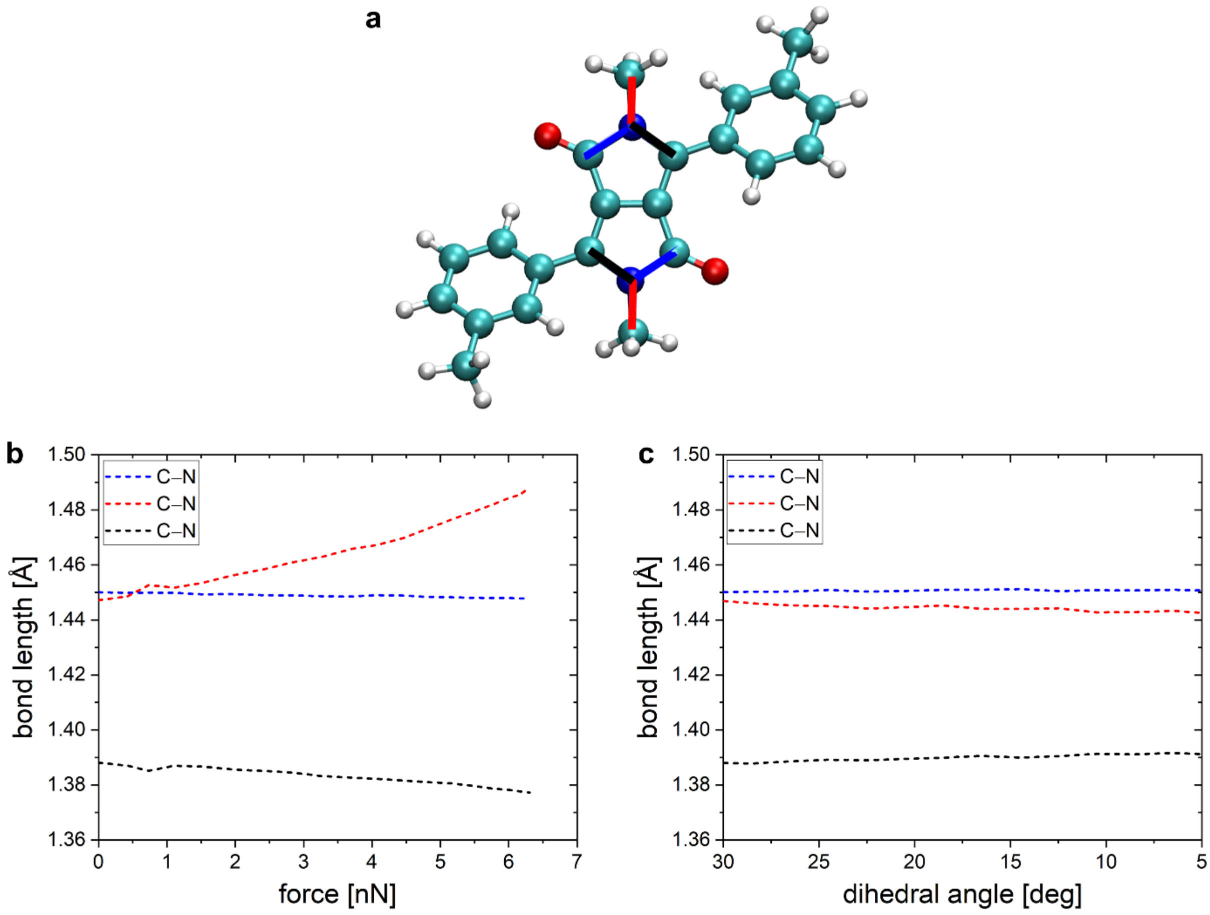

Supplementary Fig. 9. **a** Structure of *m*-MeDPP with C-N bond color code used in **b** and **c**. Changes in C-N bond lengths due to **b** the outer force and **c** constrained rotation.

The effect of the outer force and constrained rotation on the C-N bonds is shown in Supplementary Figs. 8 and 9. The C-N bond changes with external force are very small (about 0.04 Å) for both derivatives and nearly nonexistent with constrained rotation of *m*-MeDPP. In the constrained rotation of *o*-MeDPP the C-N bonds slightly change for small dihedral angles, as the Me-group of the Ph ring comes close to the N-Me-group of the DPP core. These data indicate that the C-N bond does not play a role in the shift of the optical spectra.

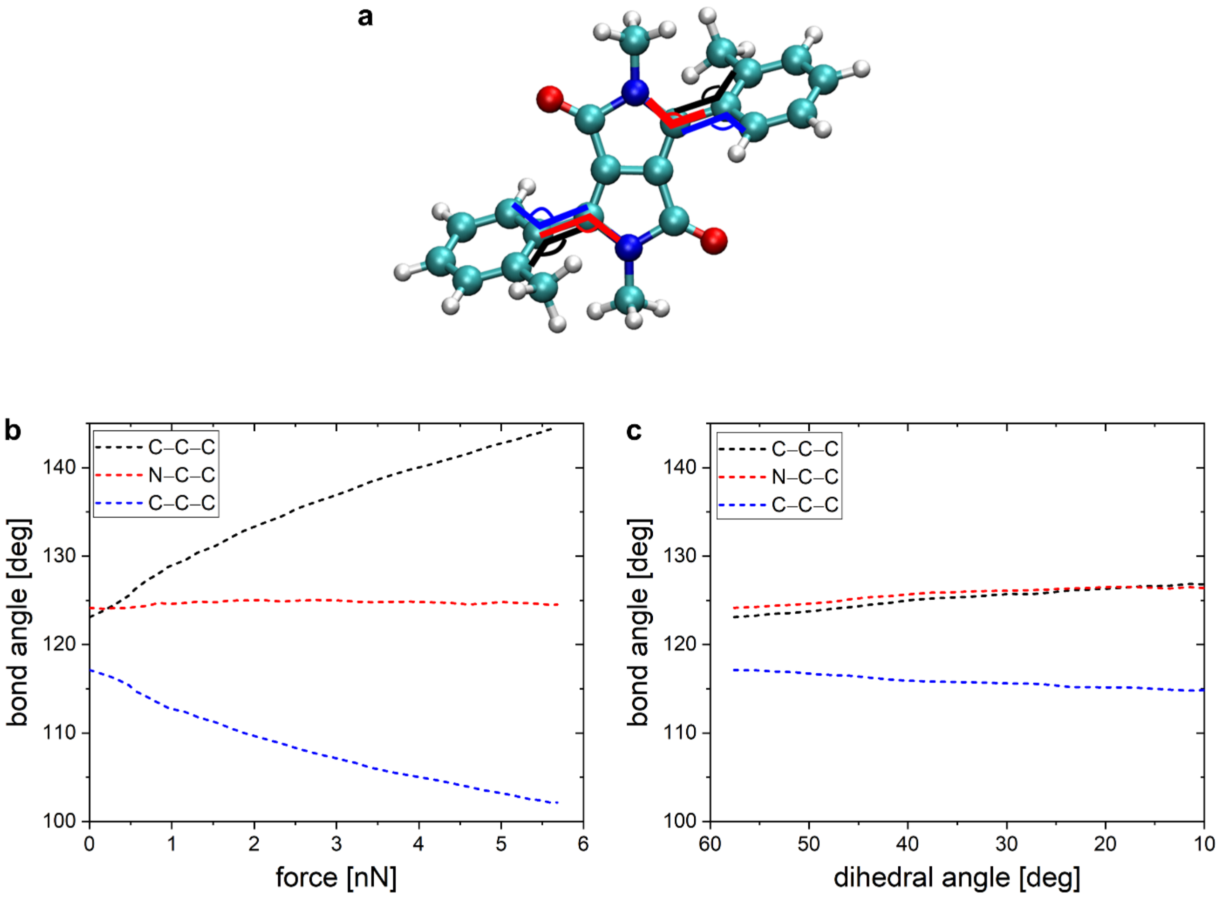

Supplementary Fig 10 a Structure of *o*-MeDPP with bond angle color code used in **b** and **c**. Changes in bond angles due to **b** the outer force and **c** constrained rotation.

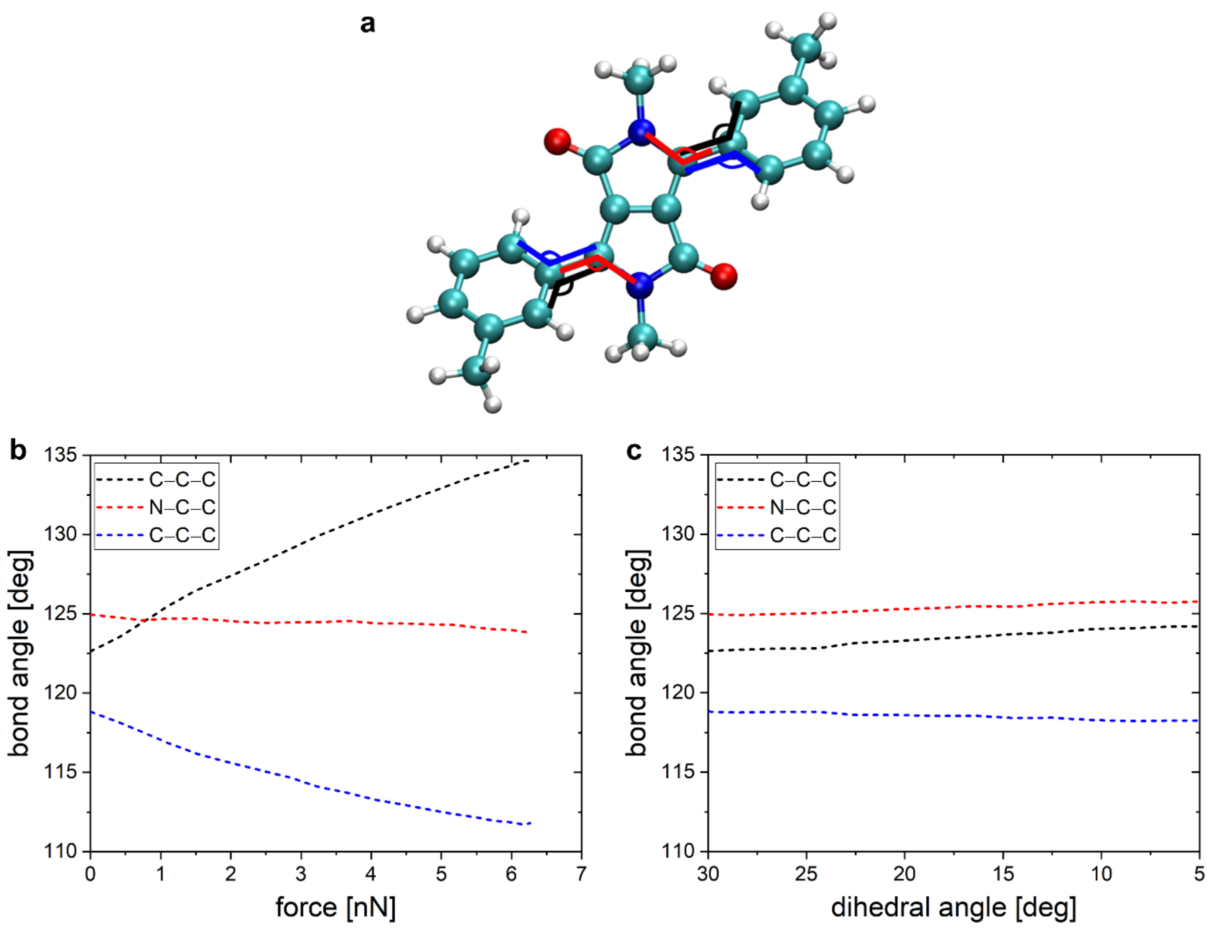

Supplementary Fig 11 a Structure of *m*-MeDPP with bond angle color code used in **b** and **c**. Changes in bond angles due to **b** the outer force and **c** constrained rotation.

The other deformation we observed was the change of bond angles between Ph ring and DPP core (see Supplementary Figs. 10 and 11). The black and blue angles change along with force, while none of these angles changes for the constrained rotation.

2.2 Investigation of rotamers

Supplementary Table 1. The rotamers of o/m-MeOBuDPP. Energies are given relative to the lowest conformer.

Conf.	Trans up-down	Trans up-up	Cis up-down	Cis up-up
ortho	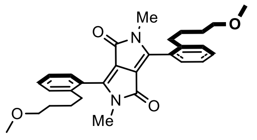	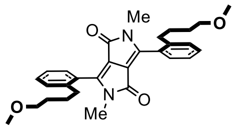	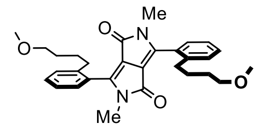	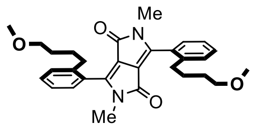
Rel. energy (kJ mol^{-1})	54	53	50	51
meta	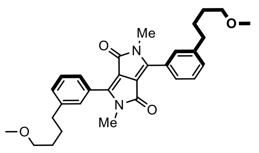	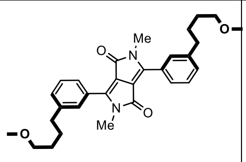	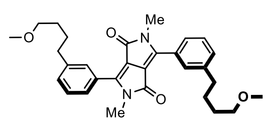	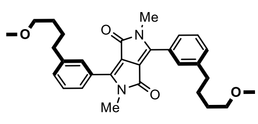
Rel. energy (kJ mol^{-1})	1	2	0	2

We consider four possible rotamers of *o/m*-MeOBuDPP: 1. trans up-down; the MeOBu-groups are in trans conformation with one of them above and the other one below the DPP core. This rotamer was discussed above and in the main text. 2. trans up-up; trans conformation with both MeOBu above the DPP plane, 3. cis up-up; cis conformation with one MeOBu above and the other one below the DPP core, 4. cis up-down; cis conformation with both MeOBu above the DPP plane. The *ortho* conformers are consistently about 50 kJ mol^{-1} higher in energy than the *meta* conformers, but the respective rotamers are very close in energy as we can see in Supplementary Table 1.

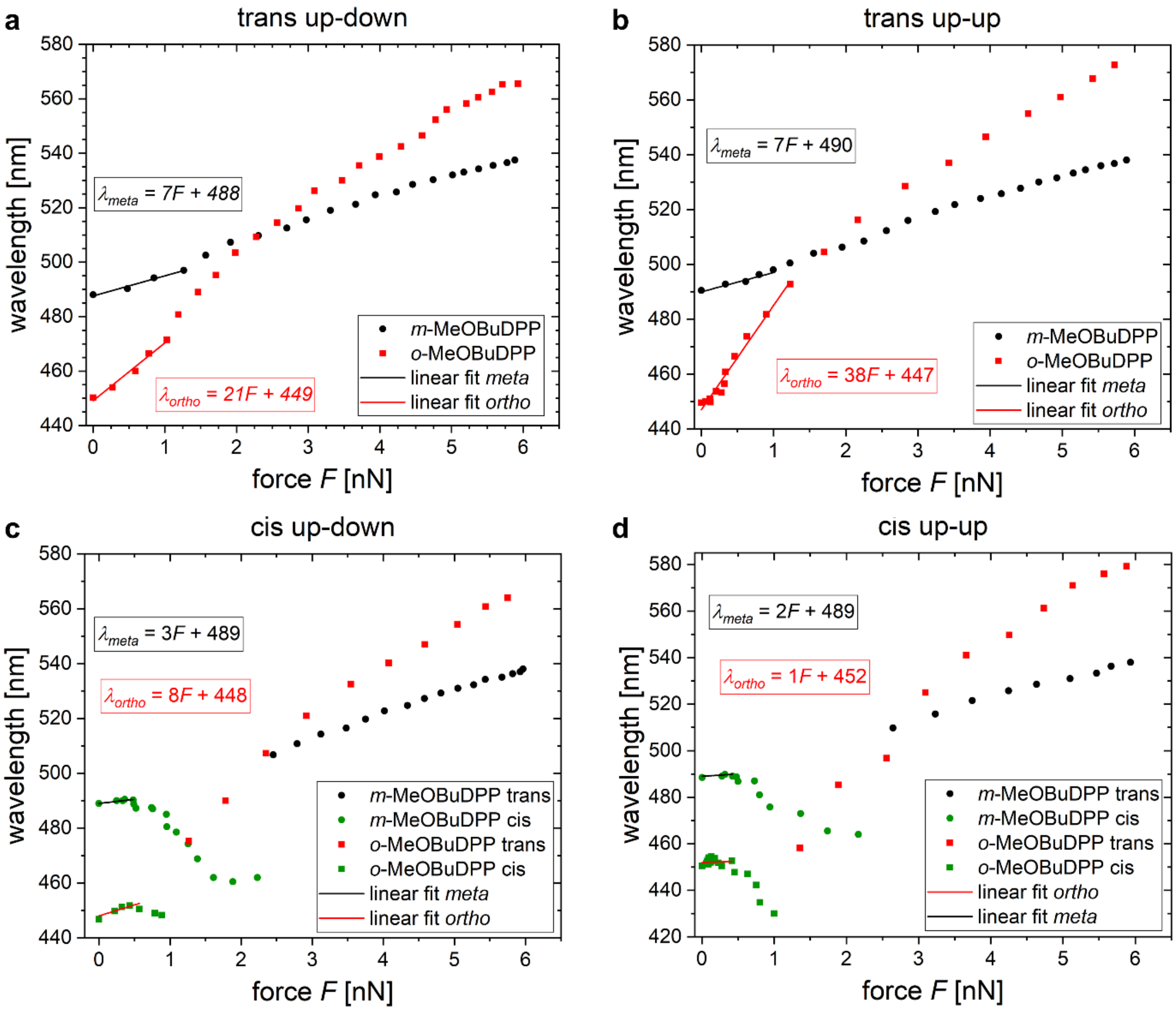

Supplementary Fig. 12. Calculated relation between the external force F and the wavelength of the charge-transfer absorption band in *o*-/*m*-MeOBuDPP for the four rotamers from Supplementary Table 1. The starting conformations are **a** trans up-down **b** trans up-up **c** cis up-down and **d** cis up-up. **c**,**d** The cis-forms (green color) transform into trans (red/black) with increasing force.

The effect of force on the optical spectra for different rotamers is summarized in Supplementary Fig. 12. The absorption wavelength of the cis rotamers is rather constant in the force range below 1 nN. Interestingly, the cis forms flip to the trans forms due to force, leading to a non-linear relation between force and CT transition wavelength in this force region. However, the resulting trans structures in c) and d) follow the trend seen in a) and b). Assuming all rotamers to be present with equal probability, the averaged optical response $\Delta\lambda_{max,abs}/F$ is 5 ± 1 nm nN^{-1} and 17 ± 2 nm nN^{-1} for *m*-MeOBuDPP and *o*-MeOBuDPP, repectively.

Supplementary Table 2. Rotamers of *o/m*-MeDPP. Energies are given relative to the lowest conformer.

Conf.	Trans up-down	Trans up-up	Cis up-down	Cis up-up
ortho	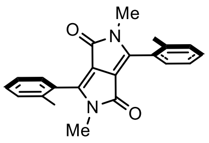	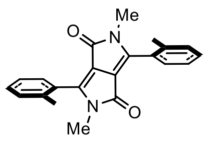	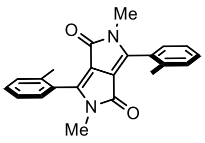	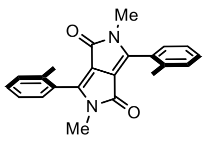
Rel. energy (kJ mol^{-1})	29	31	31	23
meta	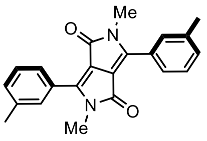	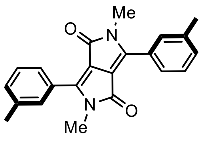	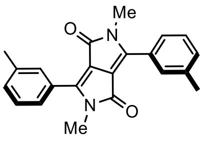	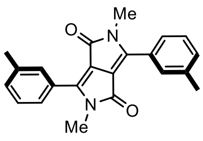
Rel. energy (kJ mol^{-1})	3	1	0	2

The rotamers of *o/m*-MeDPP given in Supplementary Table 2 show similar energetics as seen for *o/m*-MeOBuDPP (cf. Supplementary Table 1). The energy difference between *o*-MeDPP and *m*-MeDPP is slightly smaller than that of *o*-MeOBuDPP and *m*-MeOBuDPP due to decreased sterical hindrance compared to the longer chains in the latter pair.

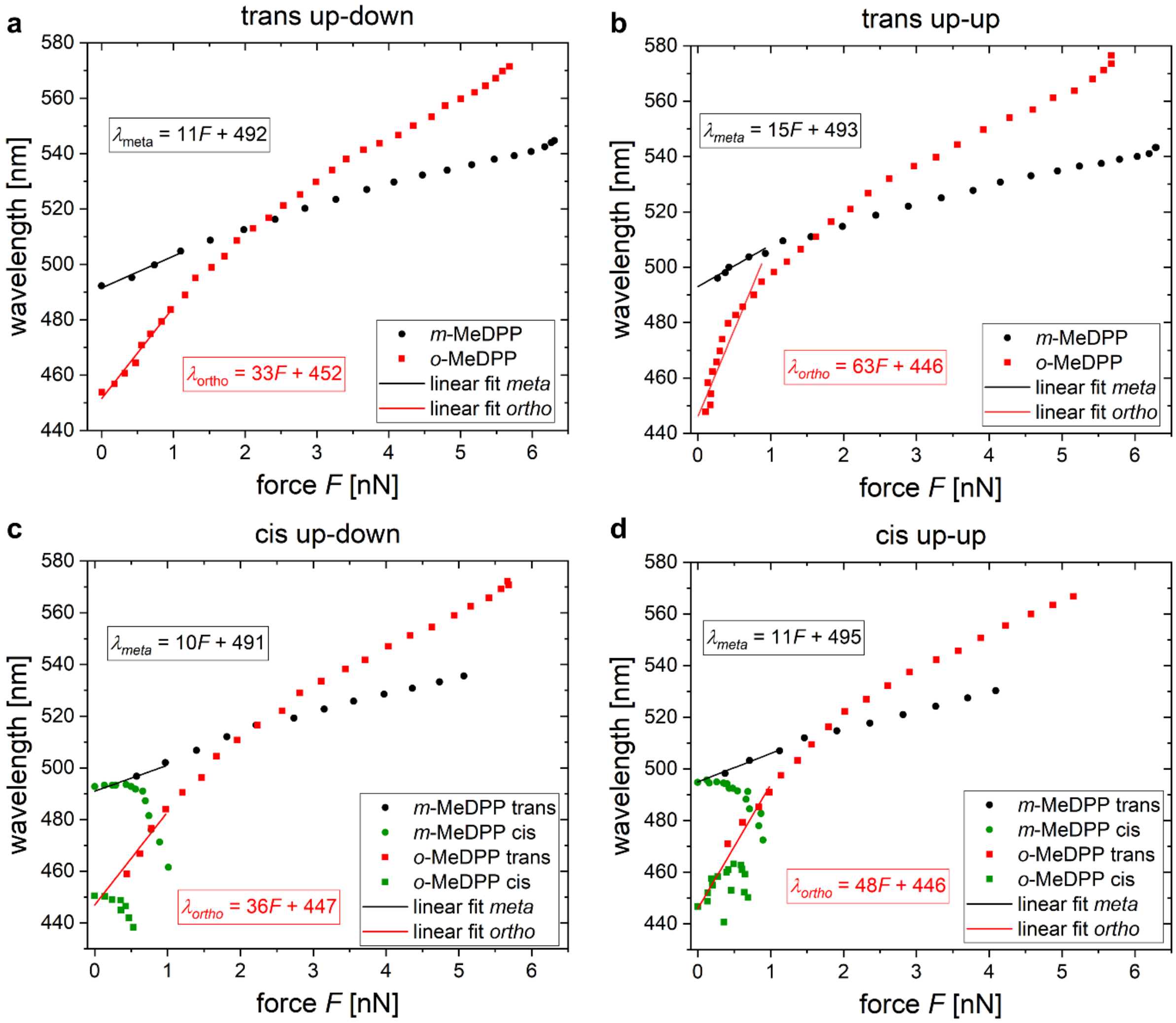

Supplementary Fig. 13. Calculated relation between the external force F and the wavelength of the charge-transfer absorption band in *o*-/*m*-MeDPP for the four rotamers from Supplementary Table 2. The starting configurations are **a** trans up-down **b** trans up-up **c** cis up-down and **d** cis up-up. **c**,**d** The cis-forms (green color) transform into trans (red/black) with increasing force.

The optical response in dependence of the force for the rotamers of *o*-/*m*-MeDPP follow the trend already seen for *o*-/*m*-MeOBuDPP. The cis-rotamers are transformed to trans-rotamers due to force and only after this, a meaningful force-wavelength relation can be extracted. We therefore use the trans-structure for the fitting process. Assuming all rotamers to be present with equal probability, this leads to an averaged $\Delta\lambda_{max,abs}/F$ of 12 ± 1 nm nN^{-1} and 45 ± 3 nm nN^{-1} for *meta* and *ortho*, respectively.

2.3 Comparison of different substituents: methyl vs. methoxybutyl

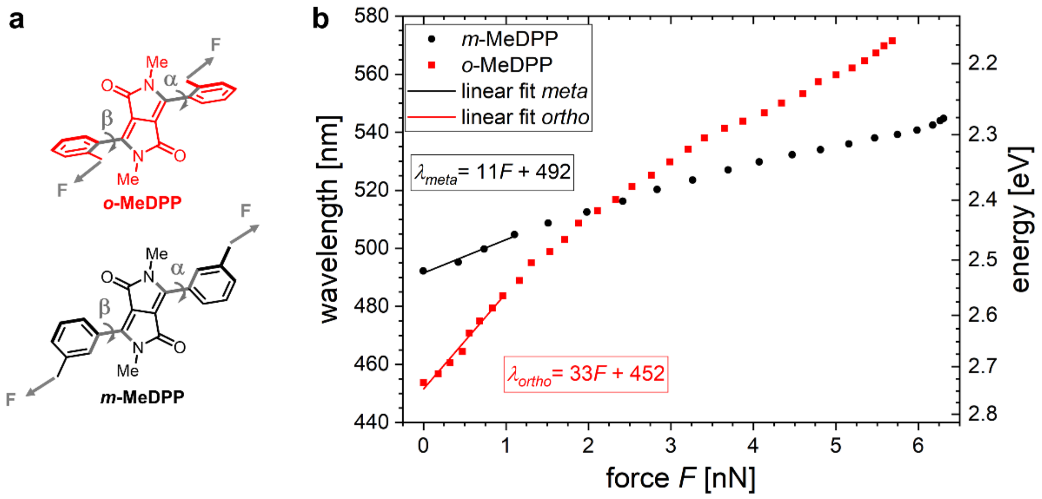

Supplementary Fig. 14. a Structures of the *ortho*- and *meta*-methyl functionalized DA springs (MeDPP). **b** Calculated relation between the external force (F) and both wavelength and energy of the charge-transfer absorption band in *o*-/*m*-MeDPP.

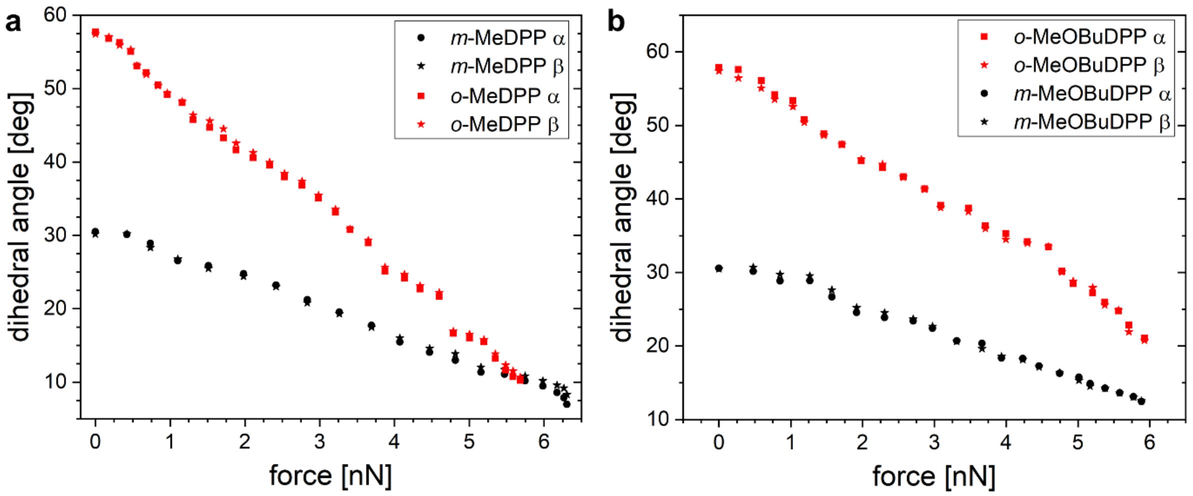

Supplementary Fig. 15. Variation of the dihedral angle with external force for **a** *o*-/*m*-MeDPP and **b** *o*-/*m*-MeOBuDPP. The angles of *m*-MeDPP and *o*-MeDPP change from 30° to 8° and from 58° to 10°, respectively, with an external force. The dihedral angles α and β are practically symmetric independent of the external force.

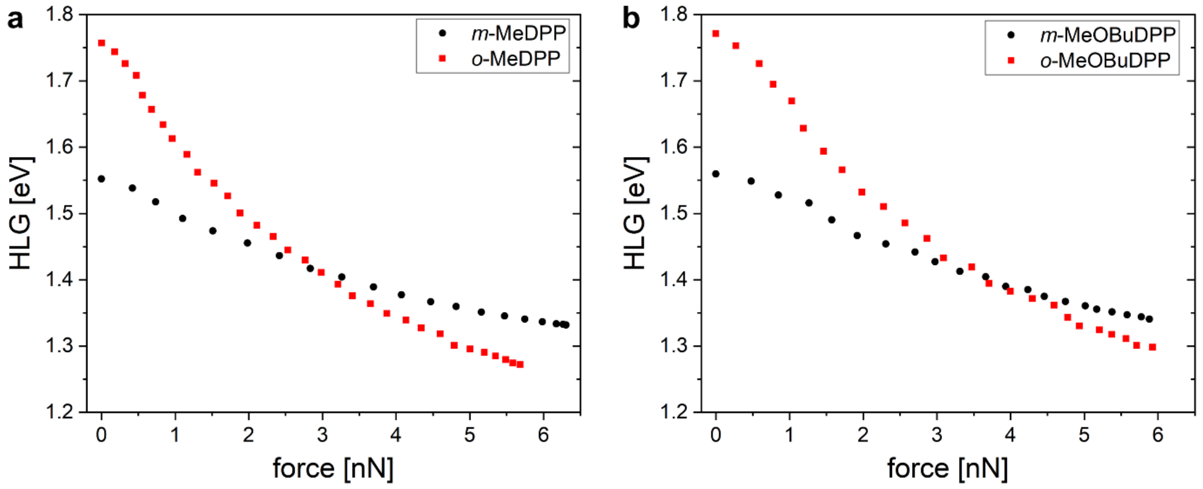

Supplementary Fig. 16. Change of HOMO-LUMO gap (HLG) in relation to external force for **a** *o*-/*m*-MeDPP and **b** *o*-/*m*-MeOBuDPP. The changes in optical spectra are reflected within single particle picture in the separation between highest occupied molecular orbital (HOMO) and lowest unoccupied molecular orbital (LUMO) energies, the HLG. The transition between these orbitals is the main contribution to the strongest optical peak. The dependence of the HLG on force is stronger for *ortho* configuration compared to *meta* configuration in accordance with Fig. 3 in the main text.

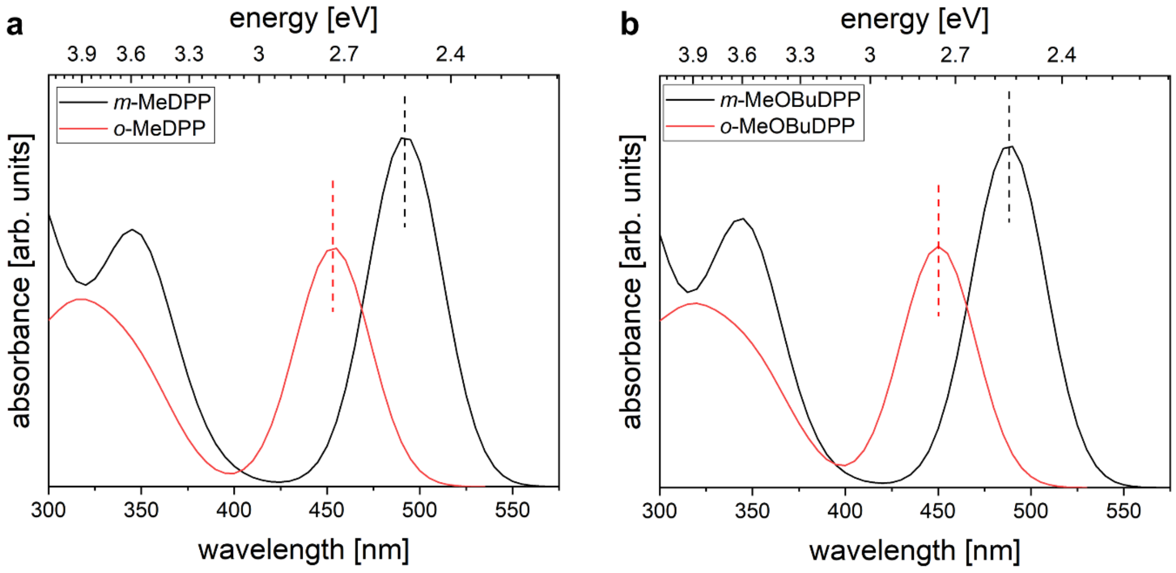

Supplementary Fig. 17. Full computational UV-Vis spectra for **a** *o-*/*m*-MeDPP and **b** *o-*/*m*-MeOBuDPP. The TDDFT CT peaks in the equilibrium geometries of *m*-MeDPP and *o*-MeDPP are found at 492 nm (2.53 eV) and 454 nm (2.73 eV), respectively. Their absorption maxima differ by about 0.21 eV (38 nm). Similar UV-vis absorption spectra are found for the methoxybutyl substituent (*o-*/*m*-MeOBuDPP). Compared to MeDPP the CT peaks are marginally shifted by about 0.02 eV (3 nm), but have an equal energetic distance of about 0.21 eV (38 nm) between *ortho*- and *meta*-DPP.

3. Mechanochromic response in polydimethylsiloxane (PDMS)

A solution of the olefin-functionalized *o*-DPP compound **7** (4.3 mg, 10 µmol) in 0.1 mL *p*-xylene was mixed with Sylgard 184 Base (2.5 g) as reported by Gossweiler et al. for different mechanophores.[9] After adding the Sylgard 184 curing agent (0.25 mL), the mixture was manually stirred vigorously for 3 min. A film was made by doctor blading on a surfactant-coated glass substrate and cured for 16 h at 65 °C. The specimen **7-K** (picture in Supplementary Fig. 18) was cut with a self-made punching tool.

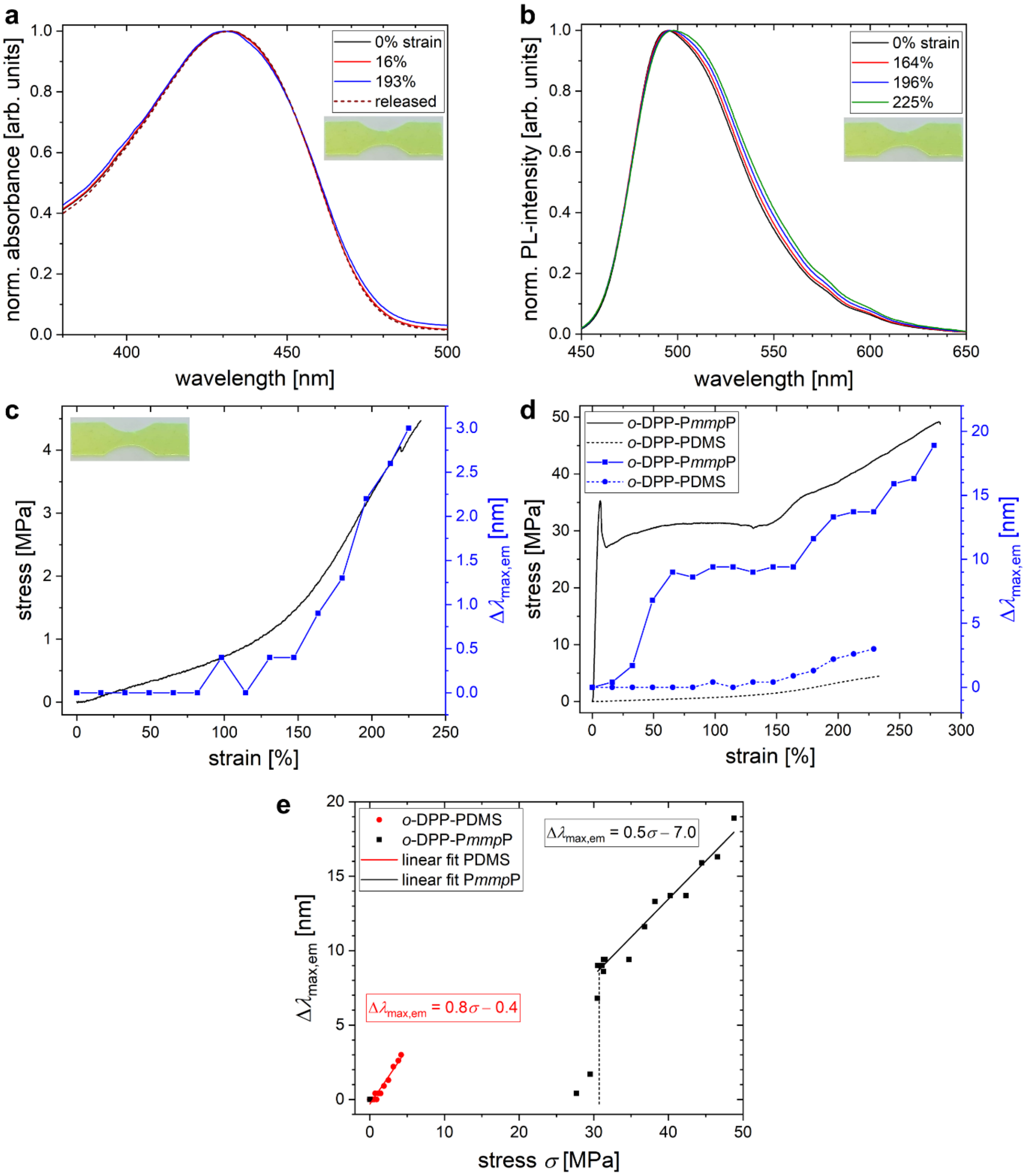

Supplementary Fig. 18. Mechanochromic response of *o*-DPP used as cross-linker in PDMS networks. **a** Normalized UV-vis absorption spectra and **b** normalized PL spectra of **7-K** during tensile testing. **c** Stress-strain curve (black) together with the corresponding shifts of the emission maxima $\Delta\lambda_{max,em}$ (blue) for **7-K** alone and **d** together with the values of **6a**. **e** Stress σ vs. $\Delta\lambda_{max,em}$ of **7-K** (red) and **6a** (black). While there is no visible change in the absorption spectra, the PL-spectra of **7-K** show a small bathochromic shift and a slight broadening of the emission band with increasing strain. The cross-linked and soft PDMS specimen shows a linear correlation of stress and emission shift, whereas necking in high T_g, tough P*mmp*P produces more scattered data points. Film thickness of **7-K**: 0.33 mm; width at mid-point: 3.3 mm.

4. Synthesis

3,6-bis(2-bromophenyl)-2,5-dihydropyrrolo[3,4-c]pyrrole-1,4-dione **(2a)**

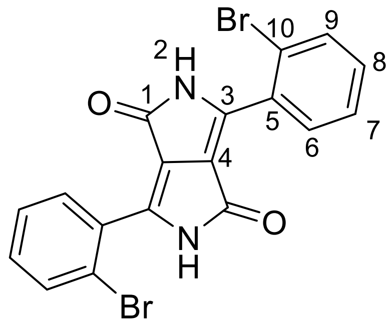

MW: 446,10

Sodium (6.40 g, 278 mmol, 3.35 eq) was dissolved in dry tert amyl alcohol (300 mL) with a catalytic amount of anhydrous $FeCl_3$ at 100 °C. After 2 h of stirring, 2-bromobenzonitrile (35.9 g, 197 mmol, 2.38 eq) was added. Diisopropyl succinate (16.8 mL, 16.8 g, 83.0 mmol) was added dropwise via a syringe pump (1.6 mL h^{-1}). After 15 h stirring at 100 °C, the purple reaction mixture was cooled down to 50 °C and methanol (300 mL) and glacial acetic acid (30 mL) were added in succession. After 15 min stirring, the red precipitate was filtered off, washed with methanol (2x200 mL) and dried in the fume hood for 3 days. The raw product mixture (5.46 g) was used in the next step without further purification. The major part of the impurities contained are the debrominated derivatives Ph(*o*-BrPh)DPP and Ph_2DPP.

Via ^{1}H NMR spectrum corrected (see Supplementary Fig. 19) yield of title compound: (3.77 g, 8.45 mmol, 10%)

^{1}H NMR (600 MHz, DMSO-d_6): δ 10.75 (br. s, 2H, H_2), 7.81 (d, J = 8.0 Hz, 2H, H_9), 7.72 (d, J = 7.7 Hz, 2H, H_6), 7.54 (t, J = 7.5 Hz, 2H, H_7), 7.47 ppm (t, J = 7.5 Hz, 2H, H_8); ^{13}C NMR (151 MHz, DMSO-d_6): δ 161.6 (C_1), 144.4 (C_3), 134.1 (C_9), 132.8 (C_8), 131.9 (C_6), 130.1 (C_5), 128.2 (C_7), 121.9 (C_{10}), 111.7 ppm (C_4); HRMS (m/z): $[M+H]^+$ calcd. for $C_{18}H_{11}Br_2N_2O_2$, 444.9182; found, 444.9183.

3,6-bis(3-bromphenyl)-2,5-dihydropyrrolo[3,4-c]pyrrole-1,4-dione **(2b)**

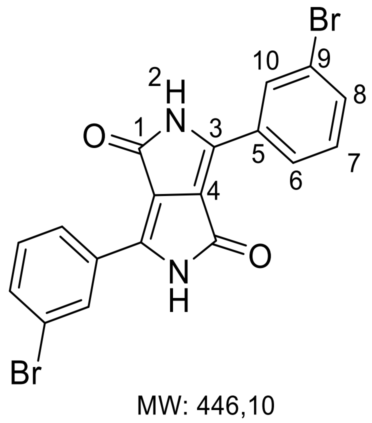

Sodium (996 mg, 43.3 mmol, 3.33 eq) was dissolved in dry tert amyl alcohol (300 mL) with a catalytic amount of anhydrous $FeCl_3$ at 100 °C. After 3 h of stirring, 3-bromobenzonitrile (5.47 g, 30.0 mmol, 2.31 eq) was added. Diisopropyl succinate (2.62 mL, 2.62 g, 13.0 mmol) was mixed with dry tert amyl alcohol (10 mL) and added dropwise via a syringe pump (1.8 mL h^{-1}). After 15 h stirring at 100 °C, the red suspension was cooled to 50 °C and methanol (60 mL) and glacial acetic acid (3.3 mL) were added in succession. After 15 min stirring, the red precipitate was filtered off, washed with methanol (2x50 mL) and dried in the fume hood for 2 days. The raw mixture was used in the next step without further purification (5.22 g, 11.7 mmol, 90%).

^{1}H NMR (600 MHz, DMSO-d_6): δ 11.43 (br. s, 2 H, H_2), 8.76 (s, 2 H, H_{10}), 8.46 (d, 2 H, H_6), 7.77 (d, 2 H, H_8), 7.56 ppm (t, 2 H, H_7).

The very poor solubility of **2b** prevented further ^{13}C NMR and HRMS characterization with the available methods. Elemental analysis was not considered to be useful due to lack of purity.

3,6-bis(2-bromophenyl)-2,5-dimethyl[3,4-c]pyrrole-1,4-dione **(3a)**

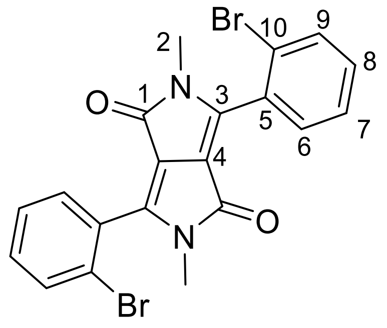

MW: 474,15

2a (3.76 g, 8.43 mmol ≡ calculated amount of **2a** in raw mixture) and K_2CO_3 (11.7 g, 84.7 mmol, 6.94 eq) were suspended in dry dimethylformamide (250 mL) under Argon atmosphere and stirred for 1 h at 120 °C. Methyl *p*-toluenesulfonate (22.6 mL, 27.9 g, 150 mmol, 12 eq) was added dropwise within 1 h at 120 °C to the purple reaction mixture. After stirring for another 2 h, the reaction mixture was cooled down to 60 °C and poured into H_2O (1.5 L). The orange precipitate was filtrated and dried for 1 week in the fume hood. After washing with hot acetonitrile (150 mL), the target compound was recrystallized (3x, chloroform/ethanol-mixture) and obtained in form of yellow crystals (2.42 g, 5.10 mmol, 60%).

sublimation point: 241 °C; ^{1}H NMR (600 MHz, $CDCl_3$): δ 7.74 (d, J = 8.1 Hz, 2 H, H_9), 7.45-7.52 (m, 4 H, $H_{6,7}$), 7.39 (m, 2 H, H_8), 3.02 ppm (2s, 6 H, H_2); ^{13}C NMR (151 MHz, $CDCl_3$): δ 161.5 (C_1), 147.3 (C_3), 133.8 (C_9), 132.2 (C_8), 131.1 (C_6), 130.0 (C_5), 128.0 (C_7), 122.6 (C_{10}), 109.9 (C_4), 28.2 ppm (C_2); HRMS (m/z): $[M+H]^+$ calcd. for $C_{20}H_{15}Br_2N_2O_2$, 472.9495; found, 472.9500; elemental analysis (%): (calcd., found for $C_{20}H_{14}Br_2N_2O_2$): C (50.66, 50.77), H (2.98, 3.09), N (5.91, 5.87).

3,6-bis(3-bromphenyl)-2,5-dimethyl[3,4-c]pyrrole-1,4-dione **(3b)**

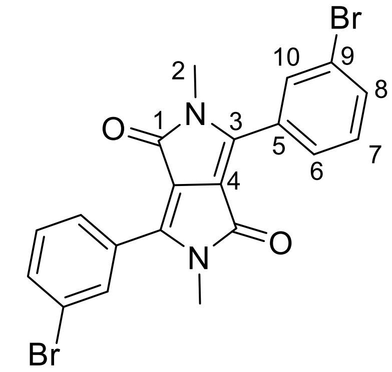

MW: 474,15

2b (3.44 g, 7.71 mmol) and K_2CO_3 (11.7 g, 84.8 mmol, 11.0 eq) were suspended in dry DMF (250 mL) under Argon atmosphere and stirred for 1 h at room temperature. A mixture of methyl *p*-toluenesulfonate (14.0 mL, 17.2 g, 92.5 mmol, 12.0 eq) and dry DMF (8 mL) was added dropwise within 1 h at 120 °C to the purple reaction mixture. After stirring another 2 h, the reaction mixture was cooled down to 60 °C and poured into H_2O (1.2 L). The red precipitate was filtered, washed with H_2O (0.4 L) and dried under vacuum at 80 °C for 18 h. The target compound was recrystallized twice, first from toluene and then from a chloroform/ethanol-mixture. The target compound was obtained as red crystals (1.53 g, 3.23 mmol, 42%).

mp: 245 °C; ^{1}H NMR (600 MHz, $CDCl_3$): δ 7.95 (s, 2 H, H_{10}), 7.78 (d, J = 7.8 Hz, 2 H, H_6), 7.59 (d, J = 8.1 Hz 2 H, H_8), 7.36 (dd, 2 H, H_7), 3.30 ppm (s, 6 H, H_2); ^{13}C NMR (151 MHz, $CDCl_3$): δ 162.3 (C_1), 147.3 (C_3), 134.4 (C_8), 131.8 (C_{10}), 130.5 (C_7), 129.7 (C_5), 127.9 (C_6), 123.0 (C_9), 109.8 (C_4), 29.5 ppm (C_2); HRMS (m/z): $[M+H]^+$ calcd. for $C_{20}H_{15}Br_2N_2O_2$, 472.9495; found, 472.9497; elemental analysis (%): (calcd., found for $C_{20}H_{14}Br_2N_2O_2$): C (50.66, 50.56), H (2.98, 3.09), N (5.91, 5.88).

*General synthesis of **4a,b***[10]. **4-Bromobutylzinc bromide:** In a flame dried Schlenk tube, zinc powder (663 mg, 10.1 mmol) and iodine (131 mg, 0.516 mmol, 5 mol%) were mixed with dry, degassed DMAc (10 mL) and stirred for 5 min at 80 °C under argon atmosphere. After decoloration of the suspension, 1,4-dibromobutane (1.42 mL, 2.59 g, 12.0 mmol, 1.2 eq) was added. The mixture was stirred fo 2.5 h at 80 °C.

In a flame dried Schlenk tube under argon atmosphere, compound **3a** (476 mg, 1.00 mmol), tris(dibenzylidene-acetone)dipalladium(0) (9.17 mg, 10.0 µmol, 1 mol%) and 2-dicyclohexylphosphino-2′,6′-dimethoxybiphenyl (SPhos, 32.4 mg, 79.9 µmol, 8 mol%) were mixed with dry, degassed DMAc (5 mL) and stirred for 5 min at 80 °C. The solution of 4-bromobutylzinc bromide was added and the mixture was stirred for 3 h at 80 °C. The reaction mixture was cooled to room temperature, mixed with an aqueous solution of HCl (1M, 16 mL) and stirred for 15 min. The organic phase was extracted with ethyl acetate (3x30 mL), washed with brine (30 mL) and dried over $MgSO_4$. The solvent was removed under reduced pressure (7 mbar, 65 °C). The product was purified by column chromatography (silica gel, petroleum ether / ethyl acetate 4:1 (v/v)) and obtained as yellow solid (81 mg, 0.14 mmol, 14%).

3,6-bis(2-(4-brombutyl)phenyl)-2,5-dimethyl[3,4-c]pyrrole-1,4-dione **(4a)**

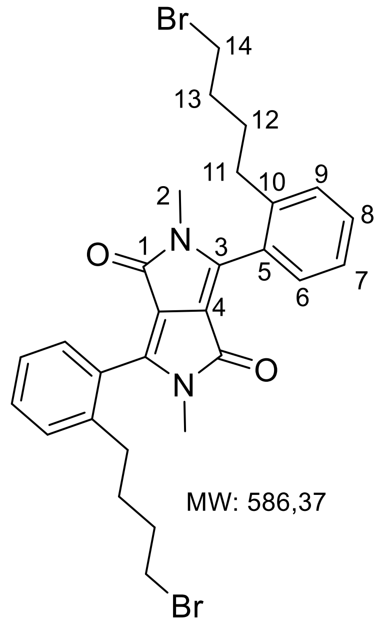

Yellow solid (14% yield)

m.p.: 174 °C; ^{1}H NMR (600 MHz, $CDCl_3$): δ 7.47-7.42 (m, 2 H, H_8), 7.39 (m, 2 H, H_9), 7.36-7.26 (m, 4 H, $H_{6,7}$), 3.40-3.35 (2 t, 4 H, H_{14}), 3.00-2.97 (2 s, 6 H, H_2), 2.99-2.62 (m, 4 H, H_{11}), 1.87-1.79 (m, 4 H, H_{13}), 1.81-1.67 ppm (m, 4 H, H_{12}); ^{13}C NMR (151 MHz, $CDCl_3$): δ 161.2 (C_1), 148.2 (C_3), 141.9-141.6 (C_{10}), 130.82 (C_8), 130.1 (C_9), 129.2-129.0 (C_6), 127.9 (C_5), 126.5 (C_7), 109.7 (C_4), 33.6 (C_{14}), 32.3-32.2 ($C_{11,13}$), 29.5 (C_{12}), 28.2 ppm (C_2); HRMS (m/z): $[M+H]^+$ calcd. for $C_{28}H_{31}Br_2N_2O_2$, 585.0747; found, 585.0754; elemental analysis (%): (calcd., found for $C_{28}H_{30}Br_2N_2O_2$): C (57.35, 57.11), H (5.16, 5.20), N (4.78, 4.67).

HSQC and HMBC experiments support our claim that the extensive splitting pattern in ^{1}H and the multiple signals in ^{13}C NMR spectra are due to the existence of rotamers at room temperature (see 7. NMR spectra).

3,6-bis(3-(4-brombutyl)phenyl)-2,5-dimethyl[3,4-c]pyrrole-1,4-dione **(4b)**

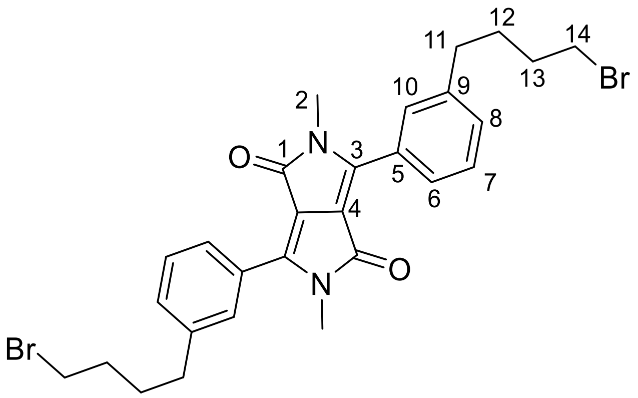

MW: 586,37

Red wax (25% yield)

^{1}H NMR (600 MHz, $CDCl_3$): δ 7.77 (s, 2 H, H_{10}), 7.65 (d, J = 7.8 Hz, 2 H, H_6), 7.45 (t, J = 7.7 Hz, 2 H, H_7), 7.33 (d, J = 7.6 Hz, 2 H, H_8), 3.45 (t, J = 6.7 Hz, 4 H, H_{14}), 3.34 (s, 6 H, H_2), 2.75 (t, J = 7.5 Hz, 4 H, H_{11}), 1.94 (m, 4 H, H_{13}), 1.85 ppm (m, 4 H, H_{12}); ^{13}C NMR (151 MHz, $CDCl_3$): δ 162.8 (C_1), 148.7 (C_3), 142.8 (C_9), 131.6 (C_8), 129.5 (C_{10}), 128.9 (C_7), 128.2 (C_5), 126.6 (C_6), 109.4 (C_4), 35.0 (C_{11}), 33.7 (C_{14}), 32.3 (C_{13}), 29.8 (C_{12}), 29.7 ppm (C_2); HRMS (m/z): $[M+H]^+$ calcd. for $C_{28}H_{31}Br_2N_2O_2$, 585.0747; found, 585.0748; elemental analysis (%): (calcd., found for $C_{28}H_{30}Br_2N_2O_2$): C (57.35, 57.32), H (5.16, 5.27), N (4.78, 4.75).

*General synthesis of **5a,b***. 4-Bromophenol (66 mg, 0.36 mmol, 3 eq), **4a** (71 mg, 0.12 mmol) and K_2CO_3 (66 mg, 0.48 mmol, 4 eq) were mixed with dry DMF (1.0 mL) under argon. The suspension was stirred for 24 h at 60 °C. After adding HCl (1M, 1.5 mL, aq.) and 30 mL H_2O, the yellow raw product was centrifuged, washed with H_2O (2x20 mL) and purified by column chromatography (silica gel, petroleum ether / ethyl acetate 4:1). The target compound was obtained as yellow solid (35 mg, 0.045 mmol, 38%).

3,6-bis(2-(4-(4-bromphenoxy)but-1-yl)phenyl)-2,5-dimethyl[3,4-c]pyrrole-1,4-dione **(5a)**

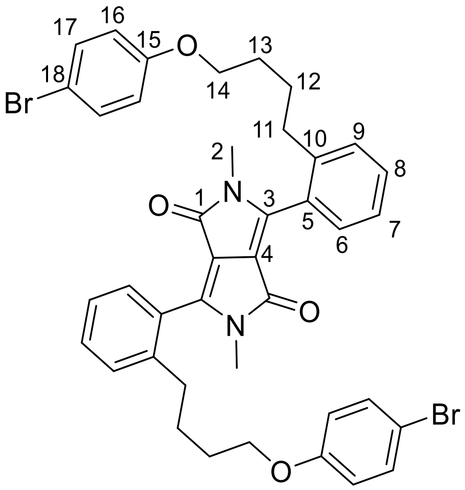

Yellow solid (38% yield)

mp: 172 °C; ^{1}H NMR (600 MHz, $CDCl_3$): δ 7.47-7.42 (m, 2 H, H_8), 7.42-7.39 (m, 2 H, H_9), 7.35-7.30 (m, 6 H, $H_{7,17}$), 7.30-7.21 (m, 2 H, H_6), 6.73-6.66 (m, 4 H, H_{16}), 3.87-3.77 (m, 4 H, H_{14}), 2.99-2.94 (2 s, 6 H, H_2), 3.00-2.68 (m, 2 H, H_{11}), 1.80-1.65 ppm (m, 8 H, $H_{12,13}$); ^{13}C NMR (151 MHz, $CDCl_3$): δ 161.8 (C_1), 158.1 (C_{15}), 148.3 (C_3), 142.0-141.8 (C_{10}), 132.3 (C_{17}), 130.8 (C_8), 130.1 (C_9), 129.2-129.0 (C_6), 127.8 (C_5), 126.4 (C_7), 116.3 (C_{16}), 112.8 (C_{18}), 109.6 (C_4), 67.8 (C_{14}), 33.0-32.8 (C_{11}), 28.9-27.5 ppm ($C_{2,12,13}$); HRMS (m/z): $[M+H]^+$ calcd. for $C_{40}H_{39}Br_2N_2O_4$, 769.1271; found, 769.1277; elemental analysis (%): (calcd., found for $C_{40}H_{38}Br_2N_2O_4$): C (62.35, 62.11), H (4.97, 5.09), N (3.64, 3.60).

HSQC and HMBC experiments support our claim that the extensive splitting pattern in ^{1}H and the multiple signals in ^{13}C NMR spectra are due to the existence of rotamers at room temperature (see 7. NMR spectra).

3,6-bis(3-(4-(4-bromphenoxy)but-1-yl)phenyl)-2,5-dimethyl[3,4-c]pyrrole-1,4-dione **(5b)**

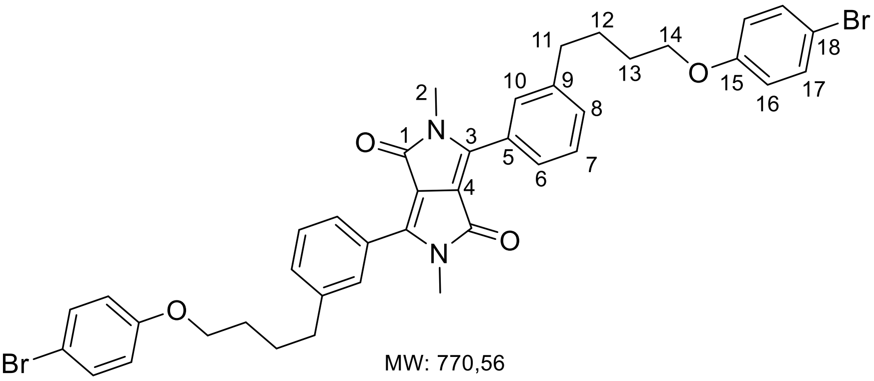

MW: 770,56

Red solid (55% yield)

mp: 138 °C; ^{1}H NMR (600 MHz, $CDCl_3$): δ 7.80 (s, 2 H, H_{10}), 7.65 (d, J = 7.8 Hz, 2 H, H_6), 7.45 (t, J = 7.7 Hz, 2 H, H_7), 7.35 (m, 6 H, $H_{8,17}$), 6.77 (m, 4 H, H_{16}), 3.96 (m, 4 H, H_{14}), 3.34 (s, 6 H, H_2), 2.79 (m, 4 H, H_{11}), 1.89-1.83 ppm (m, 8 H, $H_{12,13}$); ^{13}C NMR (151 MHz, $CDCl_3$): δ 162.8 (C_1), 158.3 (C_{15}), 148.8 (C_3), 143.1 (C_9), 132.3 (C_{17}), 131.7 (C_8), 129.5 (C_{10}), 128.9 (C_7), 128.2 (C_5), 126.5 (C_6), 116.4 (C_{16}), 112.8 (C_{18}), 109.4 (C_4), 68.1 (C_{14}), 35.6 (C_{11}), 29.7 (C_2), 28.8 (C_{13}), 27.8 ppm (C_{12}); HRMS (m/z): $[M+H]^+$ calcd. for $C_{40}H_{39}Br_2N_2O_4$, 769.1271; found, 769.1267; elemental analysis (%): (calcd., found for $C_{40}H_{38}Br_2N_2O_4$): C (62.35, 60.91), H (4.97, 5.00), N (3.64, 3.53).

1,4-benzenediboronic acid bis(pinacol)ester (**M1**)[11]

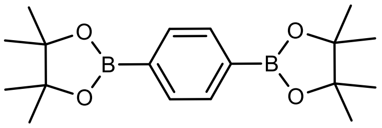

MW: 330,04

Mg (6.2380 g, 256.60 mmol, 3.0 eq.) and LiCl (7.9555 g, 187.67 mmol, 2.2 eq.) were dried inside the reaction vessel at 600 °C under high vacuum for 5 min and the vessel flooded with N_2. 1,4-Dibromobenzene (20.034 g, 84.926 mmol) and THF (dry, 400 mL) were added and the reaction heated under reflux for 3 h. The mixture was cooled to 0 °C (ice/water bath). After

addition of iPrO-Bpin (CAS: 61676-62-8, 34.5 mL, 31.4 g, 169 mmol, 2.0 eq.) the reaction was stirred for 20 h, slowly reaching room temperature. After addition of NH_4Cl (sat., aq., 11.0 mL) and HCl (aq., 2 M, 42.0 mL) the mixture was extracted with Et_2O (3x 100 mL), the organic phases extracted with brine (100 mL) and dried with $MgSO_4$. Recrystallization from ethanol (2x 500 mL) afforded **M1** as a white crystalline powder (8.18 g, 86.2 mmol, 29 %).

mp: 233 °C; ^{1}H NMR (600 MHz, $CDCl_3$): δ 7.80 (s, 4 H), 1.35 ppm (s, 24 H); ^{13}C NMR (151 MHz, $CDCl_3$): δ 134.0, 84.0, 25.0 ppm.

Full characterization is reported elsewhere[11].

5,5'-dibromo-2,2'-bis(hexyloxy)-1,1'-biphenyl (**M2**)[11]

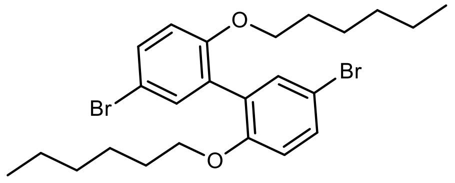

MW: 512,33

2,2'-Biphenol (10.075 g, 54.106 mmol) and K_2CO_3 (15.636 g, 113.13 mmol, 2.1 eq.) were dissolved in acetonitrile (ACN, dry, 140 mL) under N_2. After addition of 1-bromohexane (22.5 mL, 26.5 g, 161 mmol, 3.0 eq.) the reaction was heated under reflux for 18 h. The crude mixture was freed from precipitates by filtration under air (flushed with an additional 20 mL of ACN). Addition of N-Bromosuccinimide (19.1 g, 107 mmol, 2.0 eq.) and continuous purging with N_2 resulted in precipitation within 30 min. After an additional 30 min of mild agitation the crude product was isolated by filtration under air. Recrystallization from 2-propanol (3x 300 mL) afforded **M2** as colorless crystals (8.11 g, 15.8 mmol, 29%).

mp: 65-66 °C; ^{1}H NMR (600 MHz, $CDCl_3$): δ 7.37 (dd, 2 H), 7.36 (br. S, 2 H), 6.79 (d, 2 H), 3.88 (t, 4 H), 1.62 (tt, 4 H), 1.32-1.19 (m, 12 H), 0.85 ppm (t, 6 H); ^{13}C NMR (151 MHz, $CDCl_3$): δ 155.7, 134.1, 131.4, 128.7, 113.7, 112.2, 68.8, 31.7, 29.2, 25.8, 22.7, 14.2 ppm.

Full characterization is reported elsewhere[11].

General synthesis of functionalized poly(*meta*,*meta*,*para*-phenylene) (P*mmp*P) **6a,b**[11]: 1,4-benzenediboronic acid bis(pinacol)ester (**M1**) (186.8 mg, 565.8 µmol, 1.00 eq), 5,5'-dibromo-2,2'-bis(hexyloxy)-1,1'-biphenyl (**M2**) (287.0 mg, 560.2 µmol, 0.99 eq), **5a** (4.30 mg, 5.58 µmol, 0.01 eq), K_2CO_3 (498.0 mg, 3.60 mmol, 6.37 eq), tris(dibenzylideneacetone)-dipalladium(0) $CHCl_3$ (1.29 mg, 1.40 µmol, 0.25 mol%), SPhos (1.77 mg, 4.29 µmol, 0.76 mol%) and one drop of Aliquat336 were added to a 15 mL screw capped vial, and the mixture was purged with argon for 30 min. After addition of degassed toluene (1.5 mL) and degassed H_2O (1.8 mL), the reaction mixture was stirred 24 h at 70 °C. Following cooling to room temperature, the aqueous phase was removed via pipette. The organic phase was diluted with toluene (5 mL) and precipitated in methanol (80 mL). The polymer was purified by Soxhlet extraction with ethyl acetate and chloroform. After concentrating the chloroform fraction under reduced pressure, the polymer was precipitated in methanol (80 mL), filtered and dried over night in the vacuum oven (50 °C).

o-DPP-P*mmp*P **(6a)**

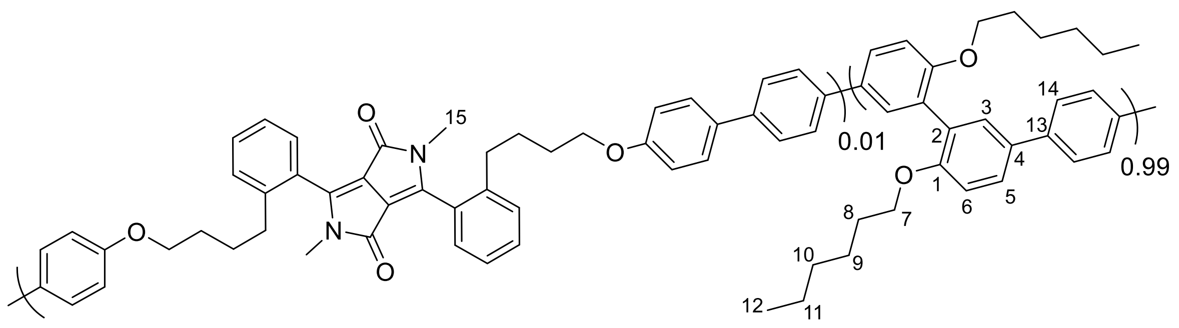

CF-fraction: yellow solid (196 mg)

^1H NMR (600 MHz, $CDCl_3$): δ 7.68 (d, 2H, H_3), 7.66 (s, 4H, H_{14}), 7.60 (dd, 2H, H_5), 7.05 (d, 2H, H_6), 4.00 (t, 4H, H_7), 3.03-2.98 (2s, H_{15}), 1.68 (m, 4H, H_8), 1.32 (m, 4H, H_9), 1.21 (m, 8H, $H_{10,11}$), 0.82 ppm (m, 6H, H_{12}); ^{13}C NMR (151 MHz, $CDCl_3$): δ 156.3 (C_1), 139.2 (C_{13}), 132.7 (C_4), 130.5 (C_3), 128.5 (C_2), 127.0 (C_{14}), 126.8 (C_5), 112.6 (C_6), 68.8 (C_7), 31.7 (C_{10}), 29.4 (C_8), 25.9 (C_9), 22.7 (C_{11}), 14.2 ppm (C_{12}).

SEC (THF): M_n = 34 kg mol^{-1}; M_w = 130 kg mol^{-1}; $Đ$ = 3.8

m-DPP-P*mmp*P **(6b)**

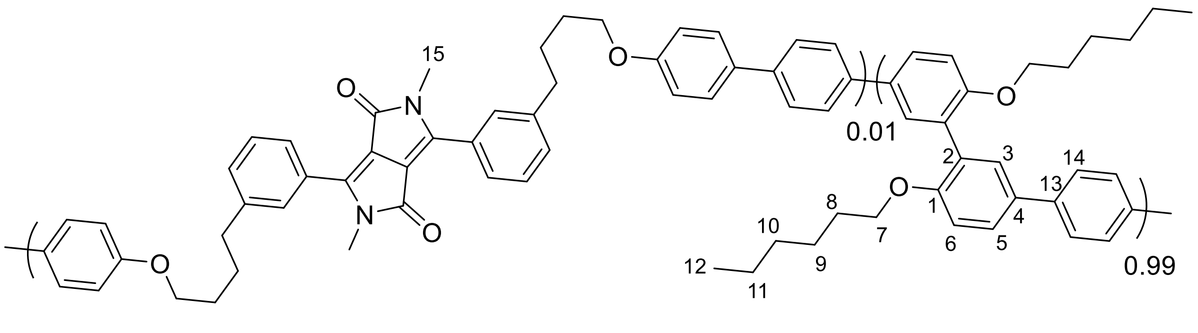

CF-fraction: orange solid (228 mg)

^{1}H NMR (600 MHz, $CDCl_3$): δ 7.67 (d, 2H, H_3), 7.66 (s, 4H, H_{14}), 7.59 (dd, 2H, H_5), 7.05 (d, 2H, H_6), 3.99 (t, 4H, H_7), 3.36 (s, H_{15}), 1.68 (m, 4H, H_8), 1.32 (m, 4H, H_9), 1.21 (m, 8H, $H_{10,11}$), 0.81 ppm (m, 6H, H_{12}); ^{13}C NMR (151 MHz, $CDCl_3$): δ 156.3 (C_1), 139.2 (C_{13}), 132.7 (C_4), 130.5 (C_3), 128.5 (C_2), 127.0 (C_{14}), 126.8 (C_5), 112.6 (C_6), 68.8 (C_7), 31.7 (C_{10}), 29.4 (C_8), 25.9 (C_9), 22.7 (C_{11}), 14.2 ppm (C_{12}).

SEC (THF): M_n = 33 kg mol^{-1}; M_w = 84 kg mol^{-1}; $Đ$ = 2.5

o-DPP-P*mmp*P (oligomeric sample) (**6a-O**)

The synthesis of **6a** was repeated using the slightly changed ratio **M1** :**M2**:**5a** of 1:0.98:0.02 and a reduced reaction time of 6 h. The precipitated polymer was fractionated by Soxhlet extraction using methyl *tert*-butyl ether (MTBE), cyclohexane (CH) and CF, and the cyclohexane fraction was used.

6a-O-CH: 16 mg, 6%; SEC (THF): M_n = 9.2 kg mol^{-1}; M_w = 13 kg mol^{-1}; $Đ$ = 1.4

Pristine non-functionalized P*mmp*P[11]

The protocol was repeated without DPP unit using a ratio **M1**:**M2** of 1:1; SEC (THF): M_n = 29 kg mol^{-1}; M_w = 88 kg mol^{-1}; $Đ$ = 3.0; NMR spectra correspond to **6a/b**.

3,6-bis(2-(4-brombutyl)phenyl)-2,5-dimethyl[3,4-c]pyrrole-1,4-dione **(7)**

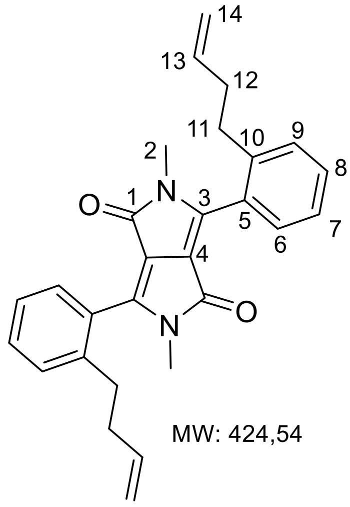

But-4-enylzinc bromide: In a flame dried Schlenk tube, zinc powder (980 mg, 15.0 mmol) and iodine (148 mg, 0.583 mmol) were mixed with dry, degassed DMAc (10 mL) and stirred for 2 min at 80 °C under argon atmosphere. After discoloration of the suspension, 4-bromobutene (1.00 mL, 1.35 g, 10.0 mmol) was added. The mixture was stirred for 16 h at 80 °C.

In a flame dried Schlenk tube under argon atmosphere, compound **3a** (475 mg, 1.00 mmol) tris(dibenzylidene-acetone)dipalladium(0) (10.0 mg, 10.9 µmol, 1 mol%) and 2-dicyclohexylphosphino-2′,6′-dimethoxybiphenyl (SPhos, 36.0 mg, 87.7 µmol, 8 mol%) were mixed with dry, degassed DMAc (5 mL) and stirred for 5 min at 80 °C. The solution of 4-bromobutylzinc bromide was added and the mixture was stirred for 16 h at 80 °C. The reaction mixture was cooled to room temperature, mixed with an aqueous solution of HCl (1M, 20 mL) and stirred for 10 min. The organic phase was extracted with ethyl acetate (3x20 mL), washed with brine (25 mL) and dried over $MgSO_4$. The solvent was removed under reduced pressure (7 mbar, 65 °C). The product was purified by column chromatography (silica gel, petroleum ether / ethyl acetate 4:1 (v/v)) and obtained as yellow solid (100 mg, 0.235 mmol, 23%).

mp: 144-146 °C; ^{1}H NMR (600 MHz, $CDCl_3$): δ 7.46-7.42 (m, 2 H, H_8), 7.40 (m, 2 H, H_9), 7.32 (m, 2 H, H_7), 7.34-7.26 (m, 2 H, H_6), 5.78-5.70 (m, 1 H, H_{13}), 4.97-4.90 (m, 2 H, H_{14}), 3.03-2.74 (m, 4 H, H_{11}), 2.97 (2 s, 6 H, H_2), 2.40-2.25 ppm (m, 4 H, H_{12}); ^{13}C NMR (151 MHz, $CDCl_3$): δ 161.9 (C_1), 148.3-148.2 (C_3), 141.8-141.5 (C_{10}), 137.6 (C_{13}), 130.7 (C_8), 130.3-130.2 (C_9), 129.1-128.9 (C_6), 127.9 (C_5), 126.4 (C_7), 115.5 (C_{14}), 109.7 (C_4), 35.2-35.1 (C_{12}), 32.9-32.7 (C_{11}), 28.2 ppm (C_2); HRMS (m/z): $[M+H]^+$ calcd. for $C_{28}H_{29}N_2O_2$, 425.2224; found, 425.2207.

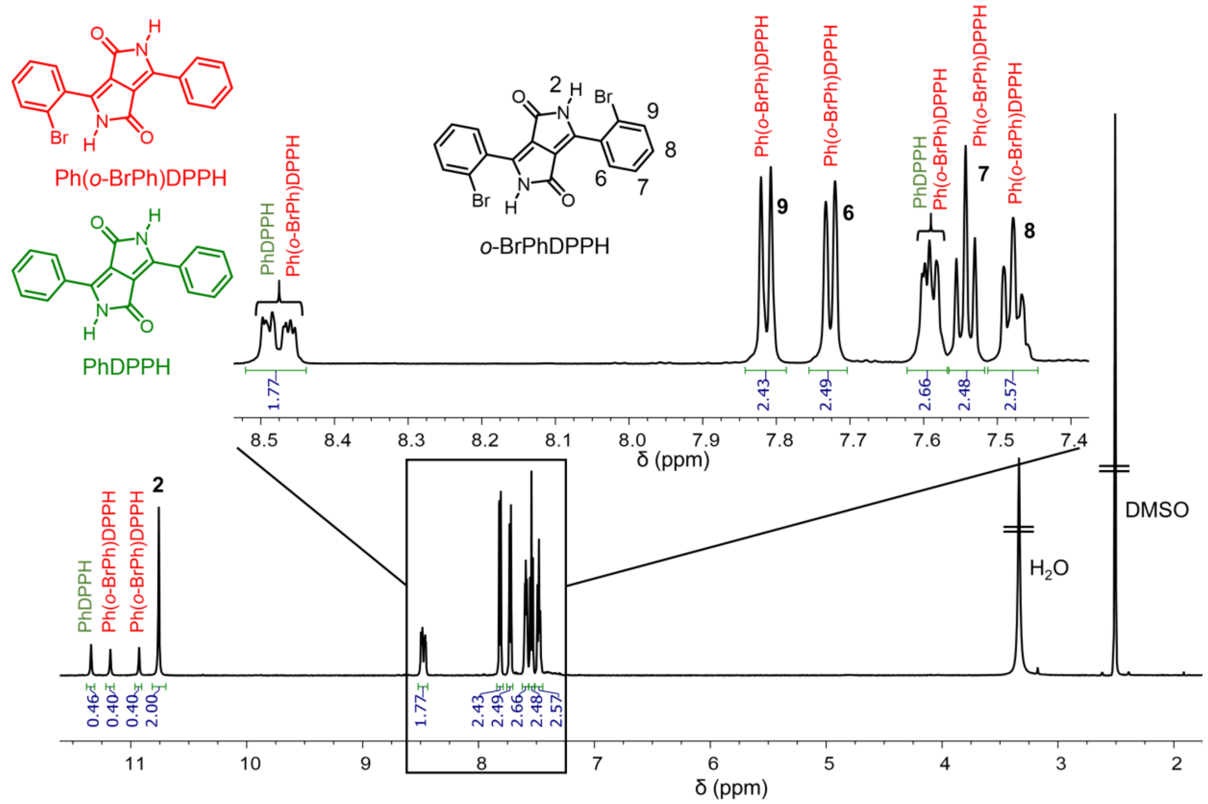

Supplementary Fig. 19. ^{1}H NMR spectrum (600 MHz) of *o*-BrPhDPPH (**2a**) in DMSO-d6. In the aromatic region, the proton signals of **2a** overlap with the signals of both mono-debrominated (Ph(*o*-BrPh)DPPH, red) and fully debrominated (PhDPPH, green) side product, which could not be separated. The amount of **2a** in the product mixture is determined by integrating the isolated N-H peaks at δ 11.4 – 10.7 ppm.

5. Thermal analysis

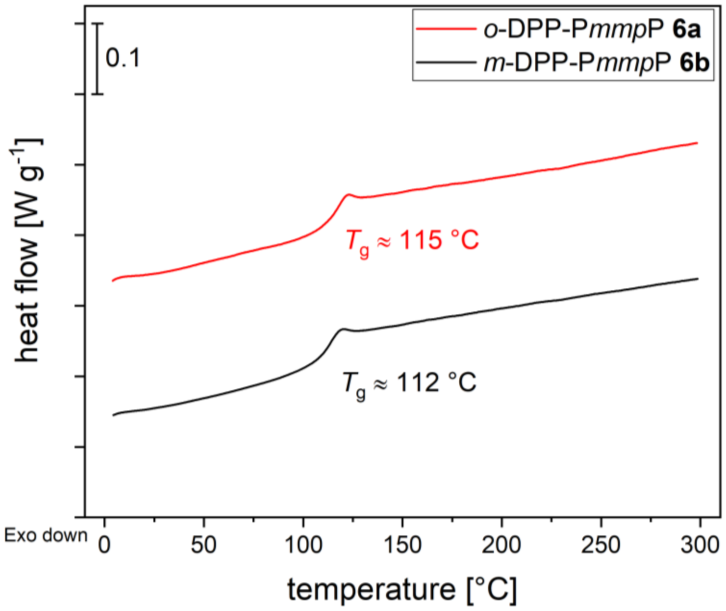

Supplementary Fig. 20. Differential Scanning Calorimetry (DSC) 2. heating scans of **6a** and **6b** (heating/cooling rate 10 °C min^{-1}).

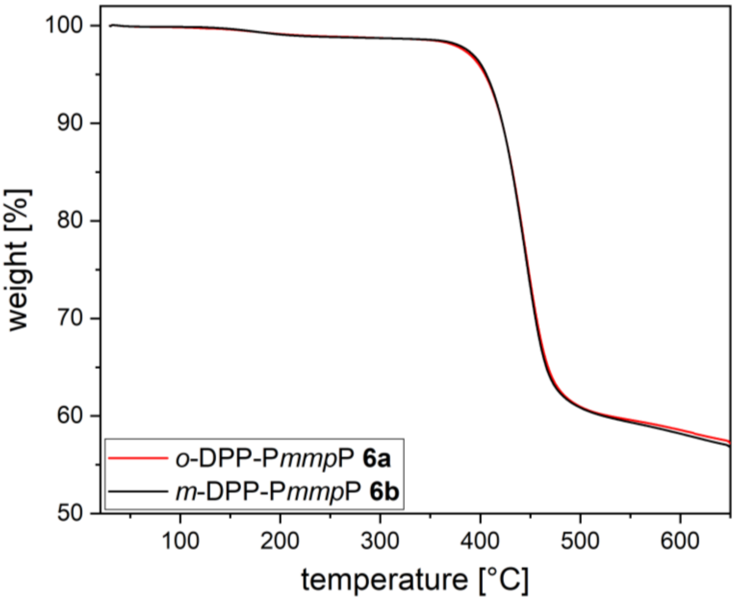

Supplementary Fig. 21. Thermogravimetric analysis (TGA) of **6a** and **6b** in N_2 atmosphere and a heating rate of 10 °C min^{-1}

6. Experimental investigation of mechanochromic response in polyphenylene

Preparation of specimens without standard dumbbell shape

***6a-K**, **6b-K**:* The under Methods described thin films of **6a** and **6b** were die-cut using a self-made punching tool of the form shown in Supplementary Fig. 24. The dimensions are length l = 25 mm and width (at mid-point) w = 3.3 mm.

Optical characterization

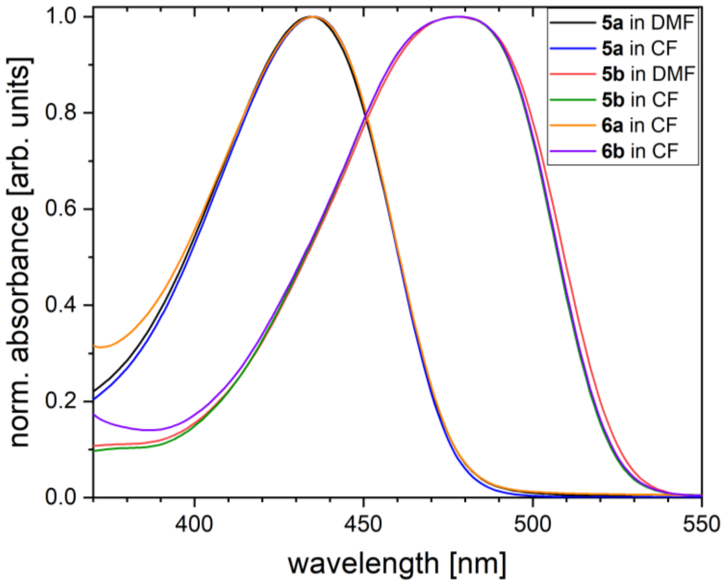

Supplementary Fig. 22. UV-vis absorption spectra of solutions of the DPP monomers *o*-LBrPhDPP (**5a**) and *m*-LBrPhDPP (**5b**) in dimethylformamid (DMF) and chloroform (CF) (conc. 0.026 mmol L^{-1}) together with the corresponding P*mmp*P polymers **6a** and **6b** in CF (conc. 1.67 g L^{-1}).

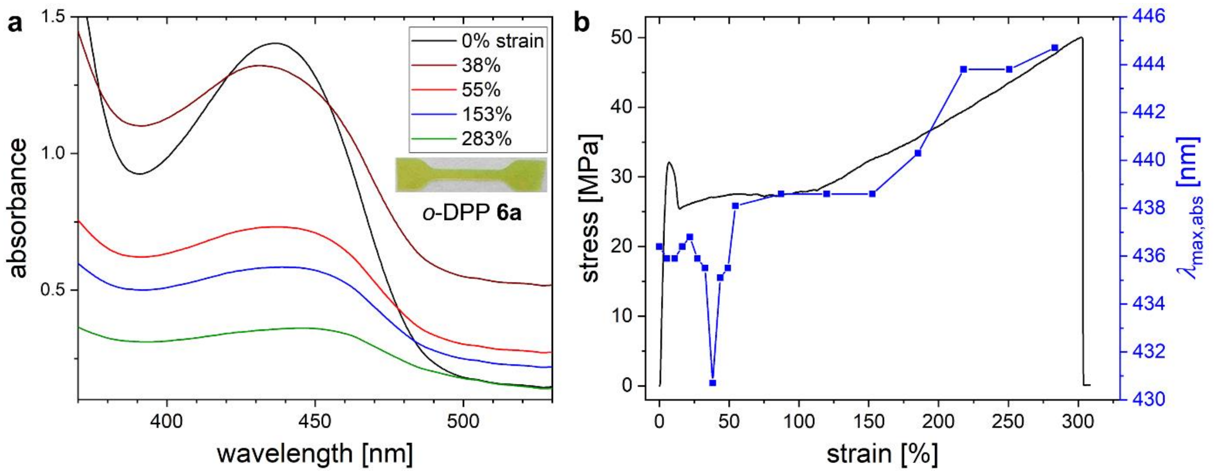

Supplementary Fig. 23. a UV-vis absorption spectra of *o*-DPP-P*mmp*P **6a** during tensile testing. **b** Stress-strain curve of **6a** (black) together with the corresponding values of absorbance maxima $\lambda_{\mathrm{max,abs}}$ (blue). UV-vis detection takes place in the mid-point of the specimen. Below 50% strain, necking has not occurred yet at the position of observation.

For the experimental mechano-optical investigations of the DPP-based DA torsional spring, blends of spring-functionalized P*mmp*P **6a** and **6b** with pristine P*mmp*P (see preparation of thin film samples) were prepared by solution blending. Dilution of the spring enabled measuring UV-vis absorption in transmission without saturation of the detector. Importantly, absorption of the P*mmp*P matrix is in the UV region (Supplementary Fig. 25a) and therefore does not interfere significantly with the DA spring.

One major drawback of the standard dumbbell shape used here is the onset of necking, which does not necessarily occur at the specimen's mid-point where the absorption/emission measurement takes place. For this reason, detection of the mechanochromic response is inevitably delayed until necking reaches this region, and thus artificially suggests a lower sensitivity of strain. For this reason, we carried out additional experiments using the specimens **6a-K** and **6b-K** shown in Supplementary Fig. 24. The shape of the latter ensures that the area of maximum tensile stress is always in the middle of the specimen and thus enables observation of mechanochromic response for low strains.

Supplementary Figs. 24a and 24b report a series of normalized and non-normalized UV-vis absorption spectra of **6a-K**, respectively, during uniaxial tensile testing. With increasing elongation, the absorption band broadens and slightly shifts towards longer wavelengths. In order to enable a clearer observation of changes in absorption, the absorption maxima $\lambda_{\mathrm{max,abs}}$

are displayed together with the corresponding stress-strain curve in Supplementary Fig. 24c. The form of the mechanochromic response (blue curve) resembles a delayed stress-strain curve. While significant changes of the optical properties are not visible in the elastic range of *o*-DPP **6a-K**, $\lambda_{max,abs}$ increases strongly right after the yield point, followed by a further increase of 4 nm before sample failure. A quantitative comparison of the stress values **6a-K** vs. **6a** (Fig. 5) is not reasonable due to the different specimen shapes used. As already shown for **6b** in the main text (Fig. 5b), **6b-K** does not show any clear mechanochromic response during tensile testing (Supplementary Fig. 24d).

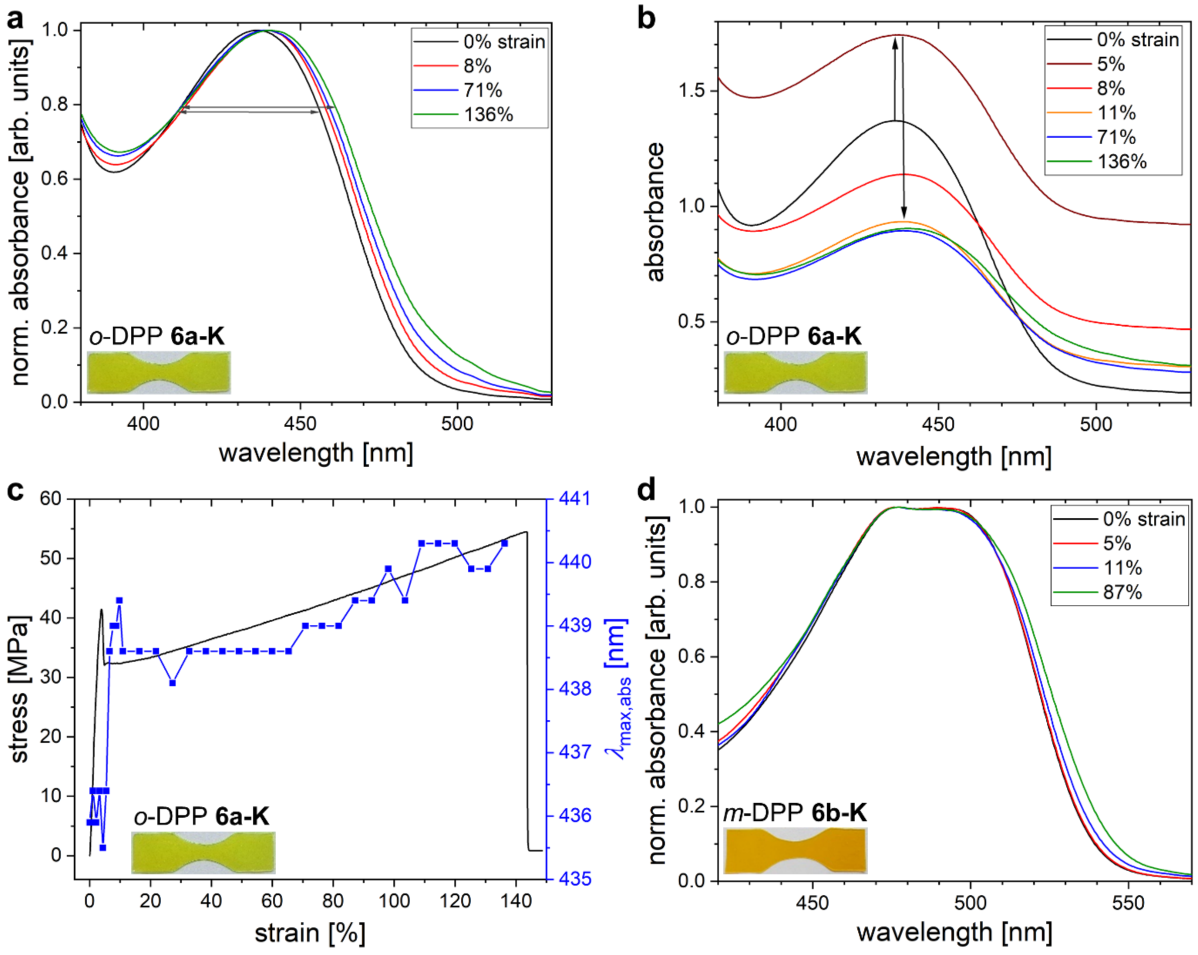

Supplementary Fig. 24. a Normalized and **b** non-normalized UV-vis absorption spectra of *o*-DPP-P*mmp*P **6a-K** during tensile testing using a different, non-standard specimen shape. **c** The corresponding stress-strain curve of **6a-K** (black) in relation to the absorbance maxima $\lambda_{max,abs}$ (blue). **d** Normalized UV-vis absorption spectra of *m*-DPP-P*mmp*P **6b-K** during tensile testing using the same, non-standard specimen shape.

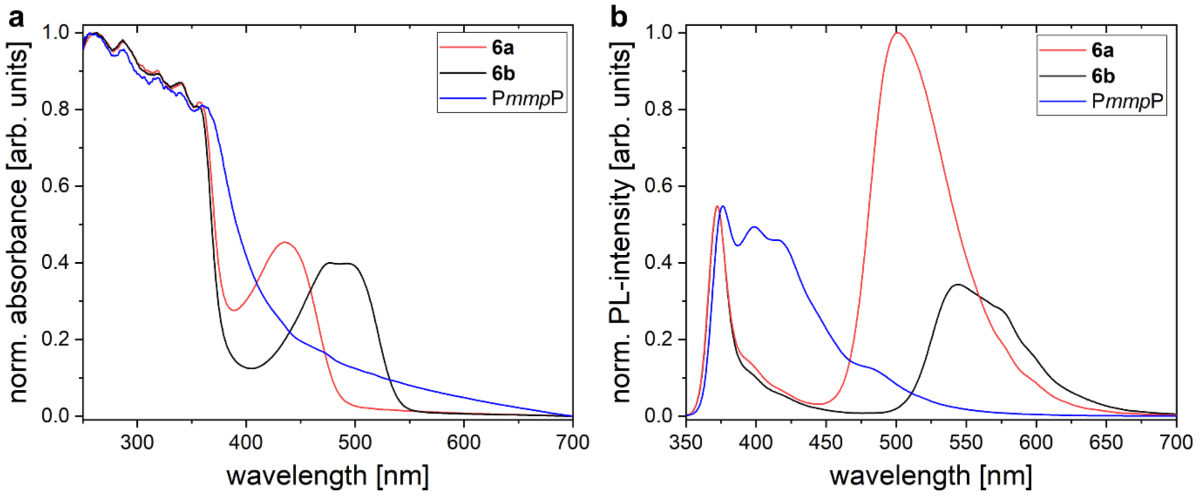

Supplementary Fig. 25. a UV-vis absorption spectra and **b** PL spectra of thin films of *o*-DPP-P*mmp*P **6a**, *m*-DPP-P*mmp*P **6b** together with a similar shaped specimen of pristine P*mmp*P.

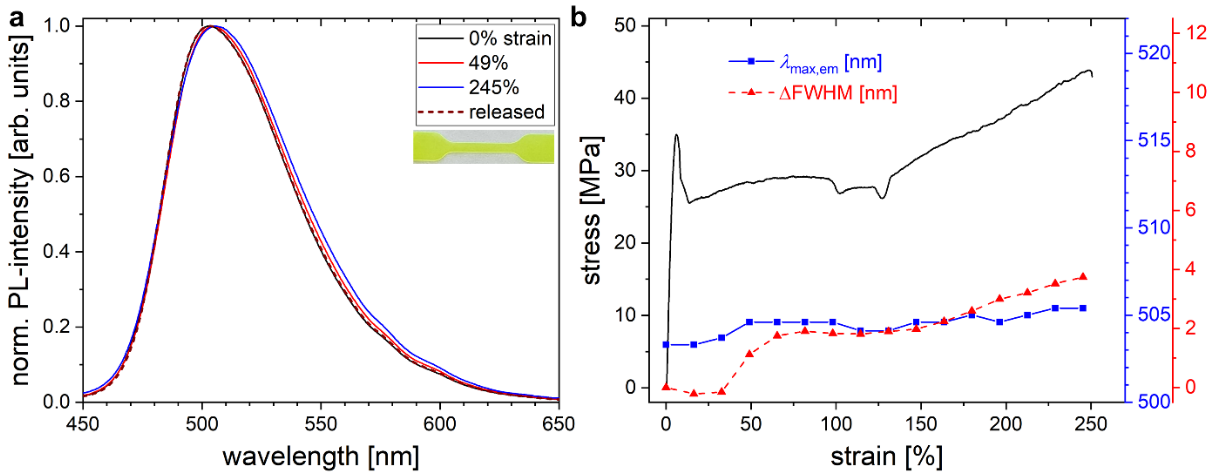

Supplementary Fig. 26. Characterization of the mechanochromic response of *o*-DPP-P*mmp*P oligomers (**6a-O-CH**) with M_n = 9.2 kg mol^{-1}; M_w = 13 kg mol^{-1}. A thin film specimen of **6a-O-CH** (16 mg) in 180 mg pristine P*mmp*P was produced analogous to **6a** (see preparation of thin film samples under methods). **a** Normalized PL-spectra during tensile testing. **b** Stress-strain curve (black) together with the detected emission maxima $\lambda_{max,em}$ (blue) and the changes in Full Width at Half Maximum of the emission bands (ΔFWHM, red). The observed shifts of $\lambda_{max,em}$ and FWHM are clearly smaller compared to the ones of **6a** shown in Fig. 5e. This poor mechanochromic response of **6a-O-CH** with its similar strain-induced orientation but most likely negligible amount of entanglements support our theory of force-induced planarization of a DA torsional spring presented in Fig. 1.

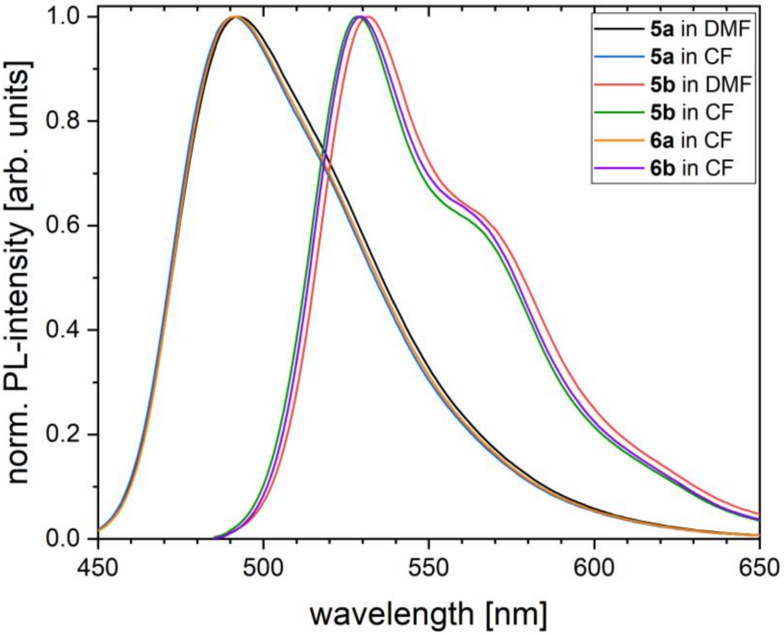

Supplementary Fig. 27. PL spectra of solutions of the DPP monomers *o*-LBrPhDPP (**5a**) and *m*-LBrPhDPP (**5b**) in dimethylformamide (DMF) and chloroform (CF) (conc. 0.026 mmol L^{-1}) together with the corresponding P*mmp*P polymers **6a** and **6b** in CF (conc. 1.67 g L^{-1}). Excitation wavelengths: $\lambda_{ex,ortho}$ = 435 nm and $\lambda_{ex,meta}$ = 475 nm.

7. NMR Spectra

^{1}H NMR spectrum of **2a** in DMSO-d6

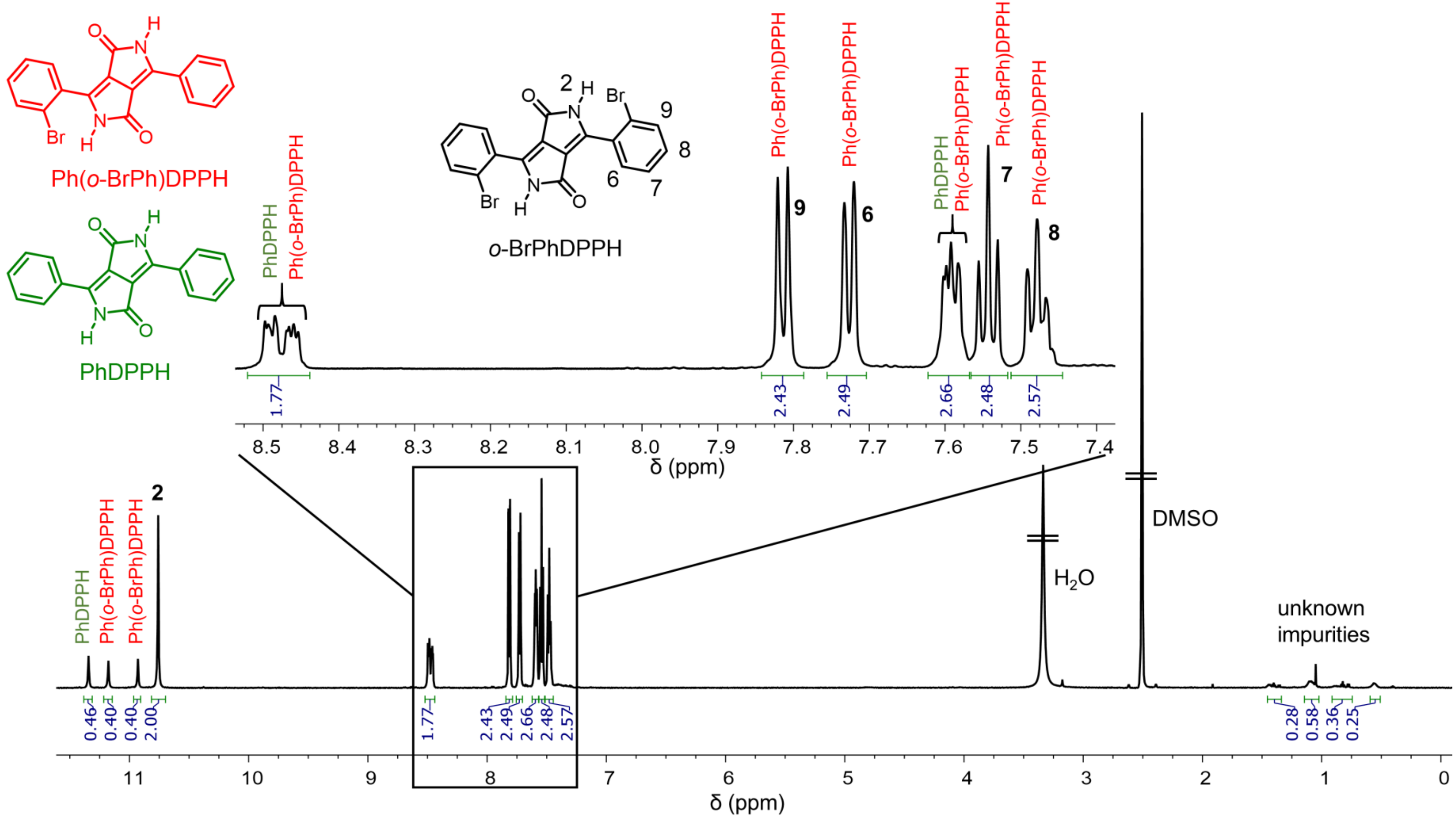

^{13}C NMR spectrum of **2a** in DMSO-d6

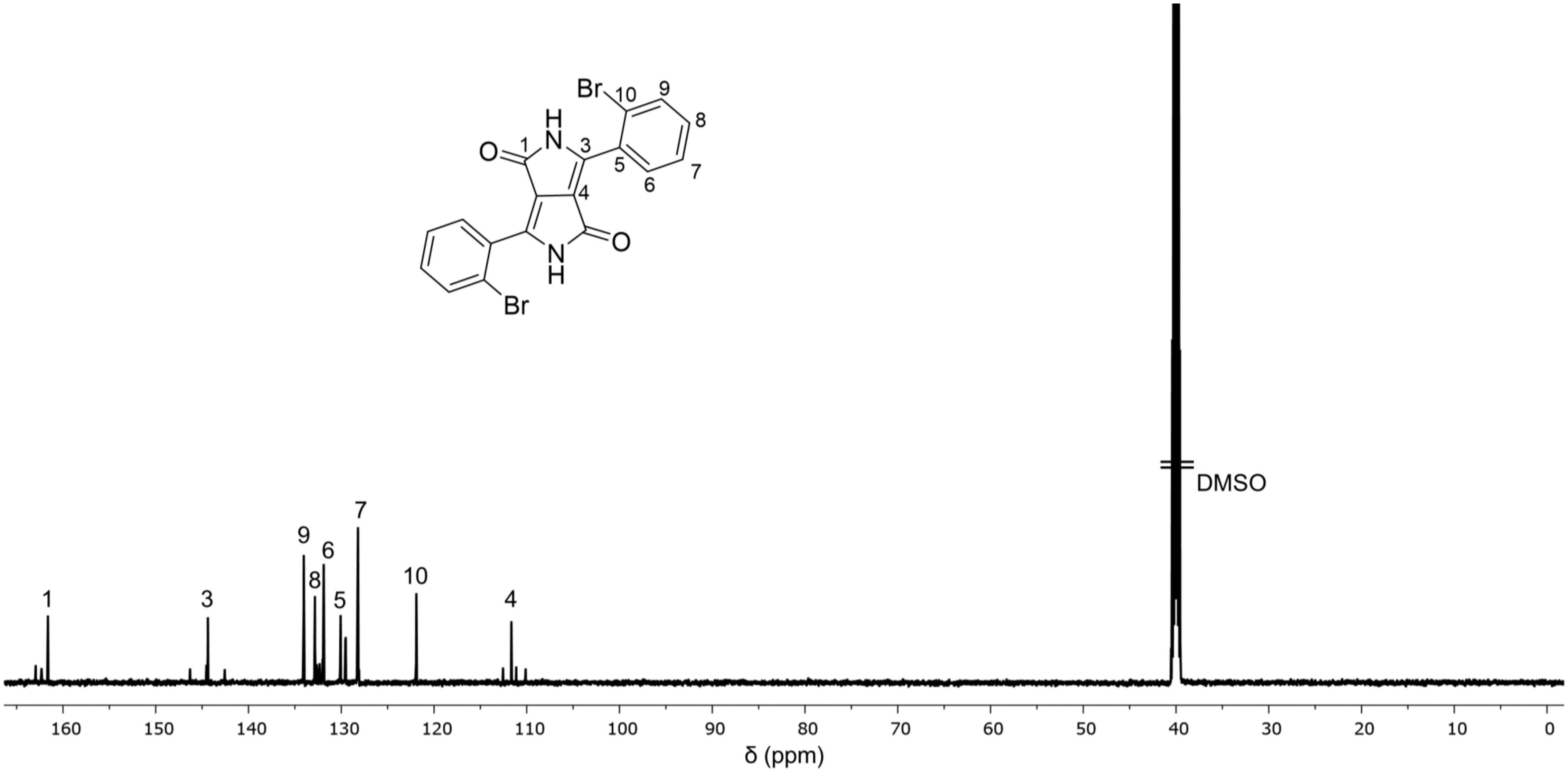

^{1}H NMR spectrum of **2b** in DMSO-d6

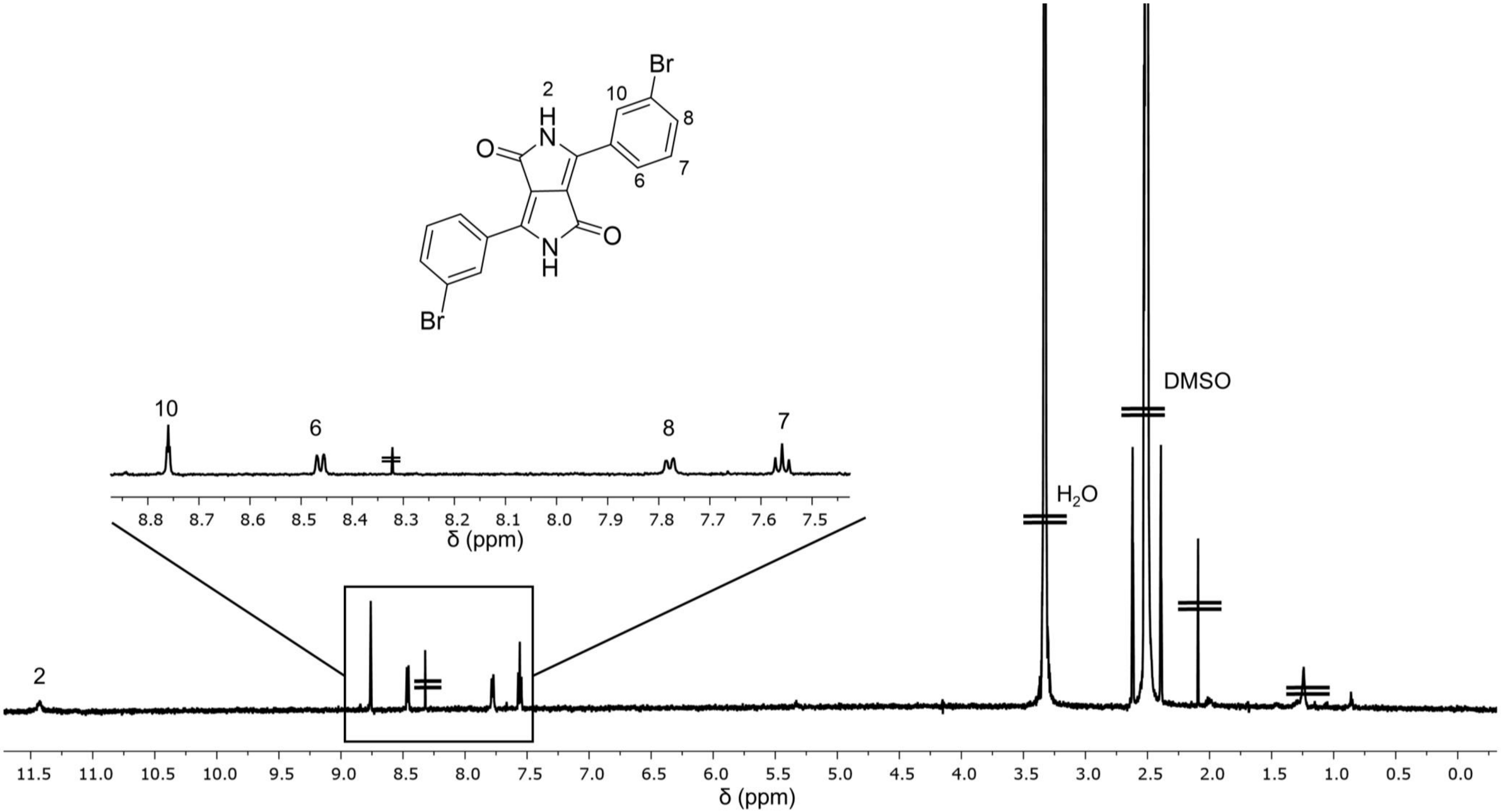

^{1}H NMR spectrum of **3a** in $CDCl_3$

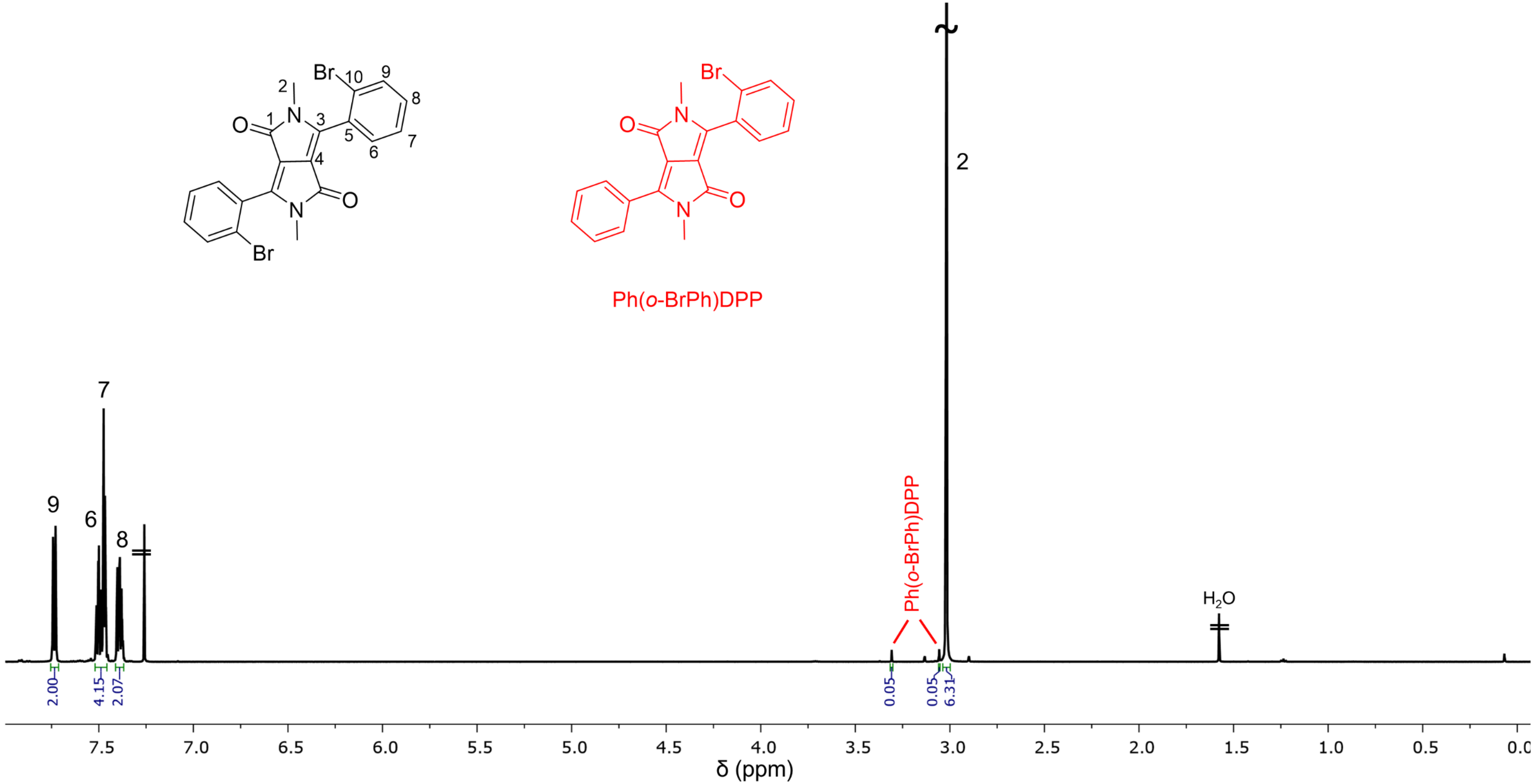

^{13}C NMR spectrum of **3a** in $CDCl_3$

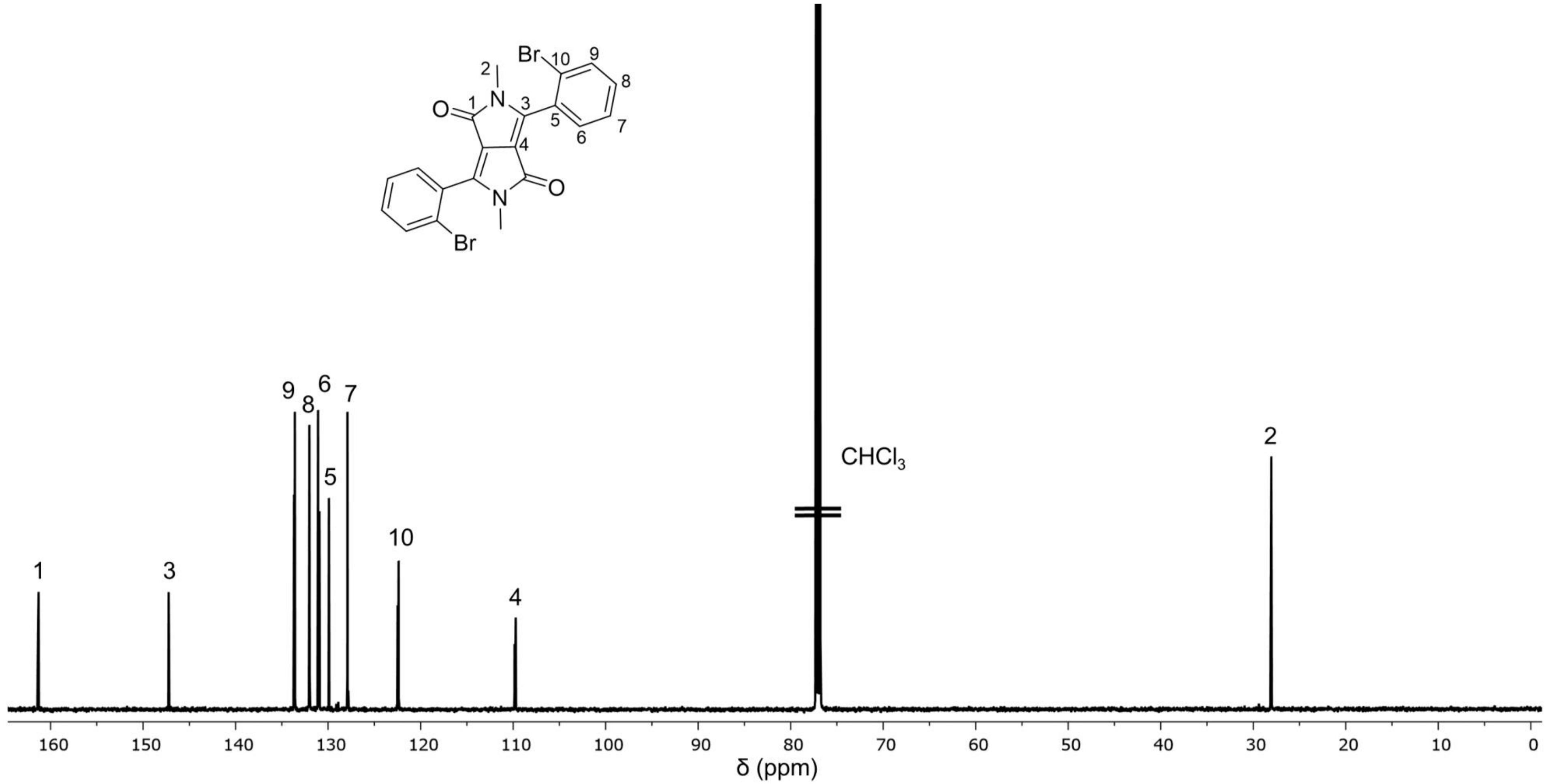

^{1}H NMR spectrum of **3b** in $CDCl_3$

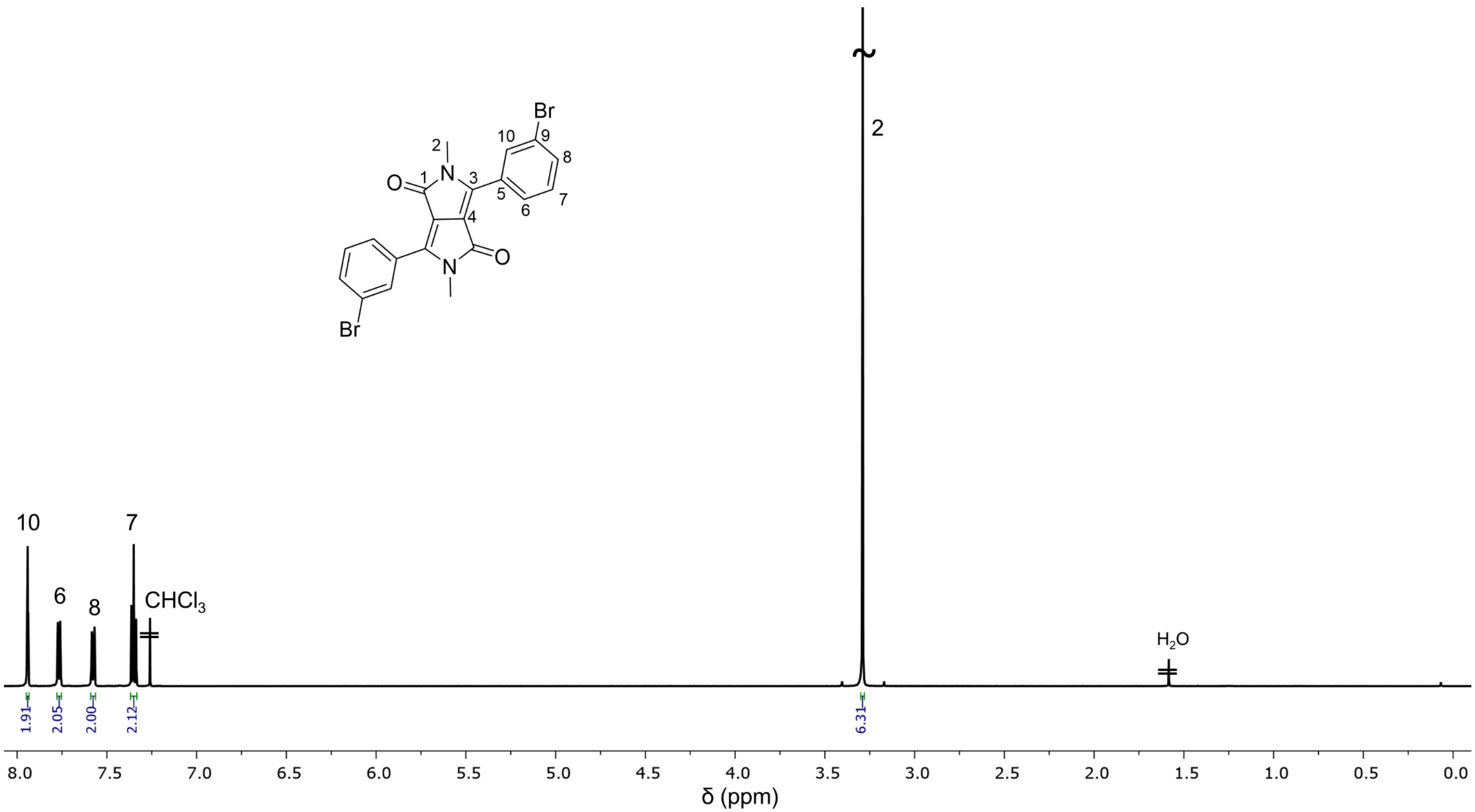

^{13}C NMR spectrum of **3b** in $CDCl_3$

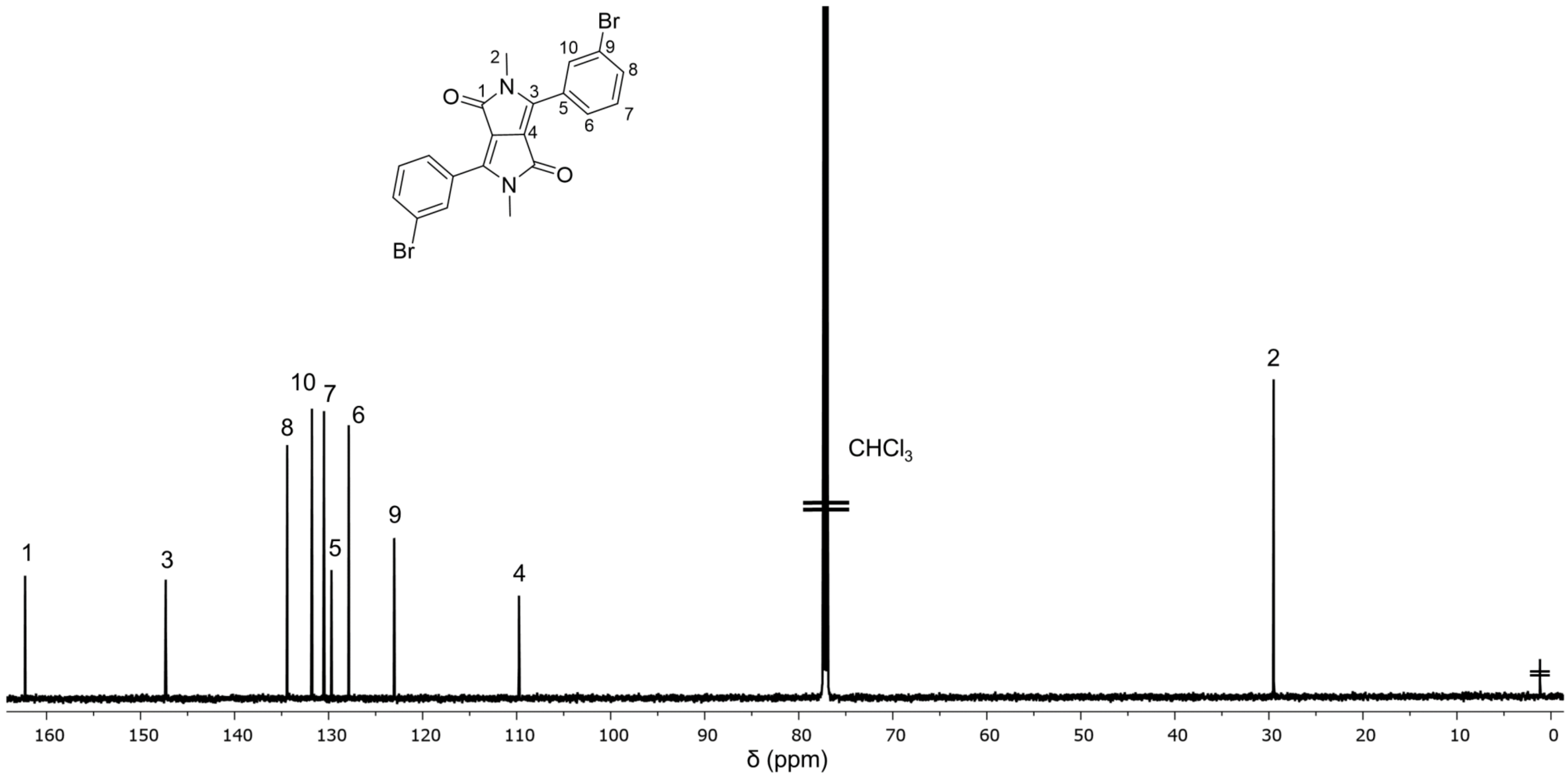

^{1}H NMR spectrum of **4a** in $CDCl_3$

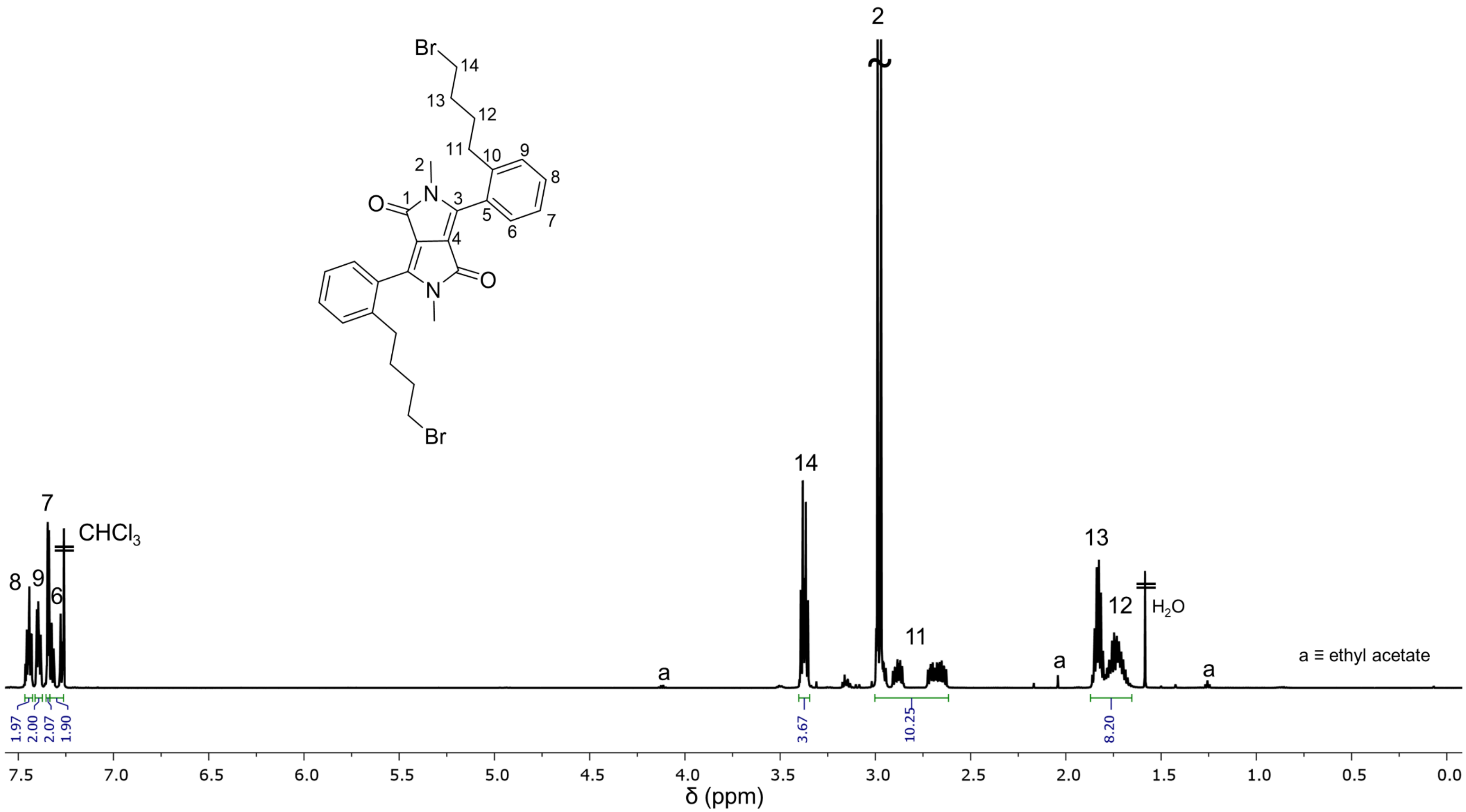

^{13}C NMR spectrum of **4a** in $CDCl_3$

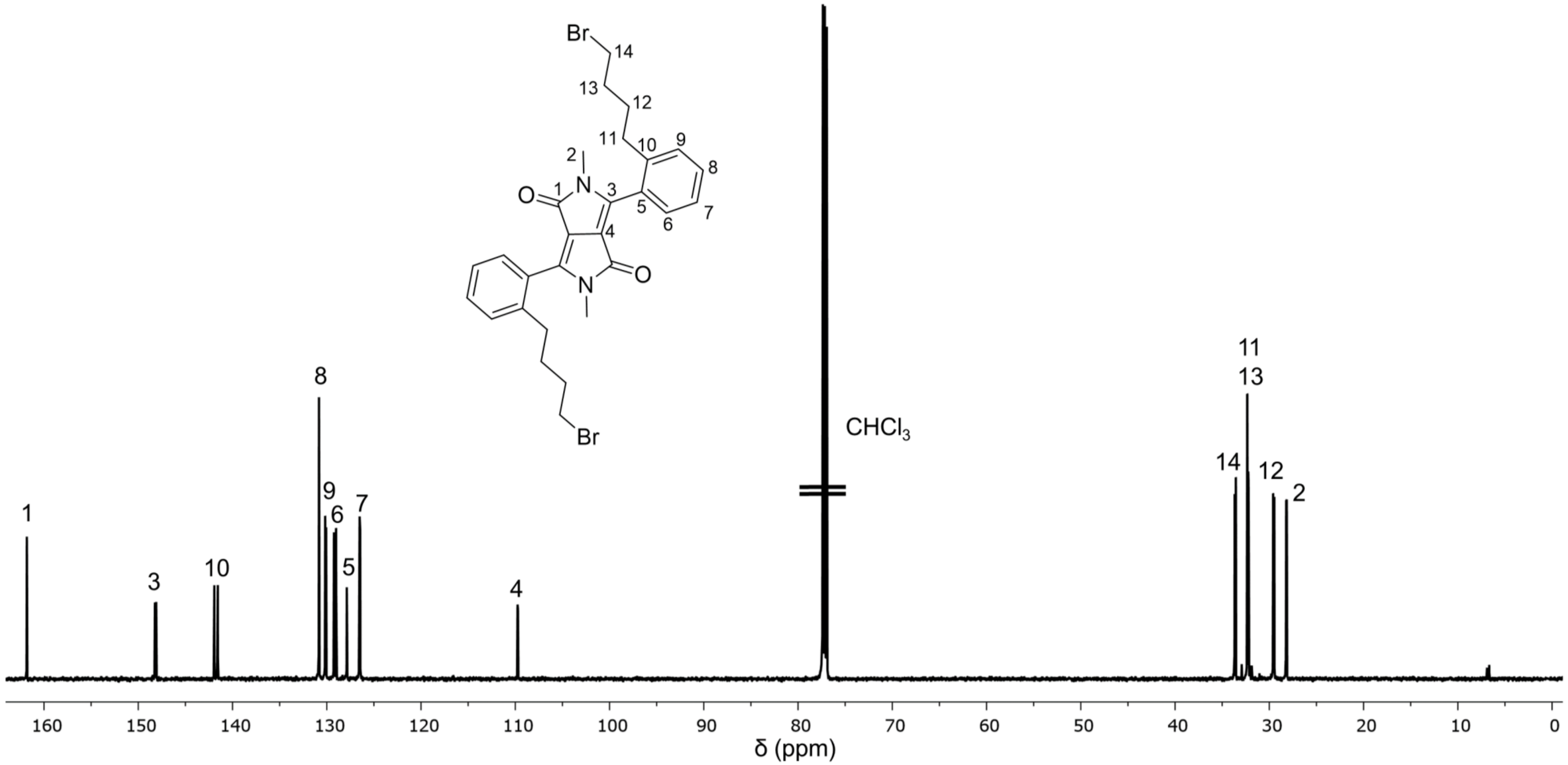

^{1}H NMR spectrum of **4b** in $CDCl_3$

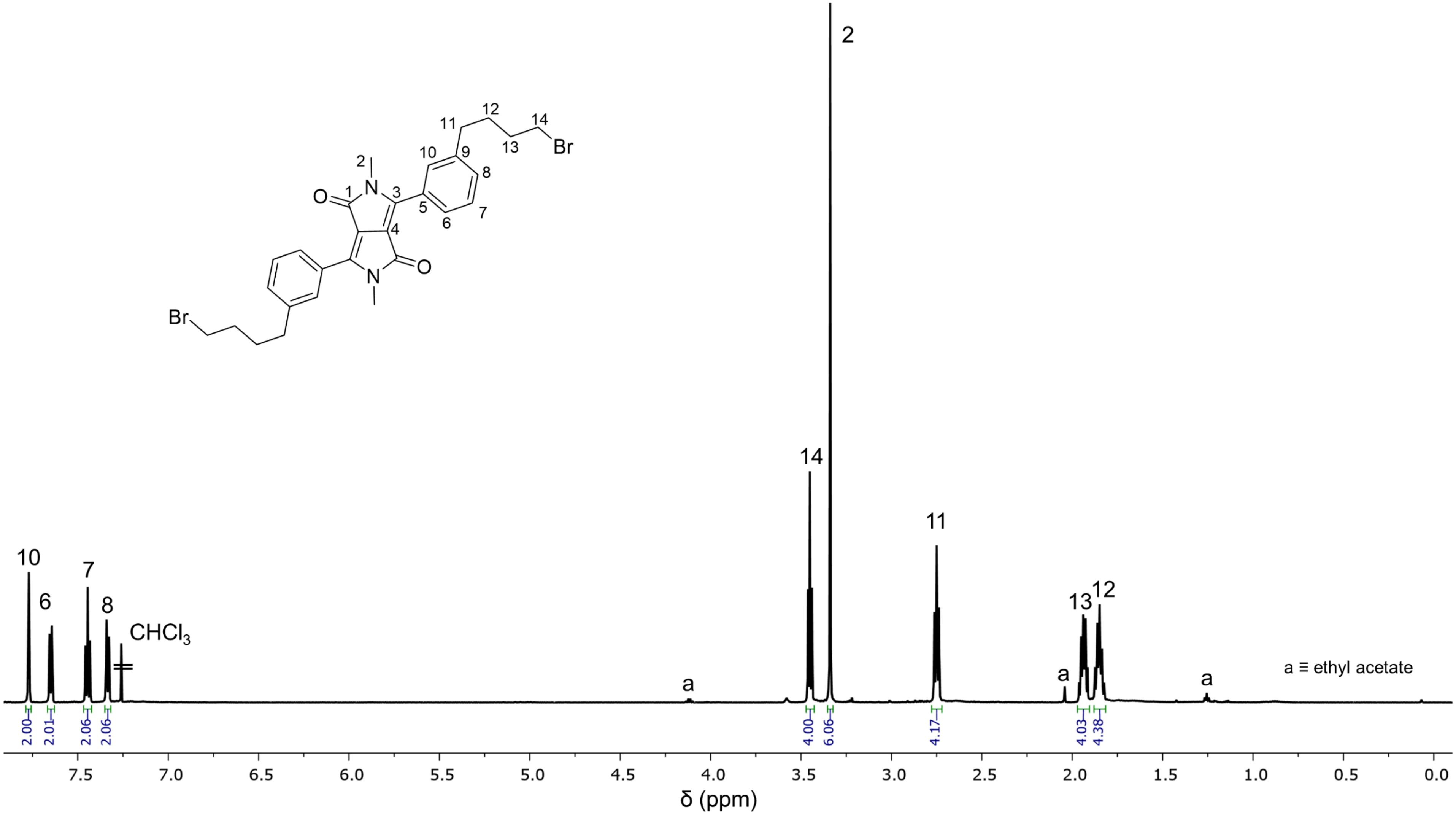

^{13}C NMR spectrum of **4b** in $CDCl_3$

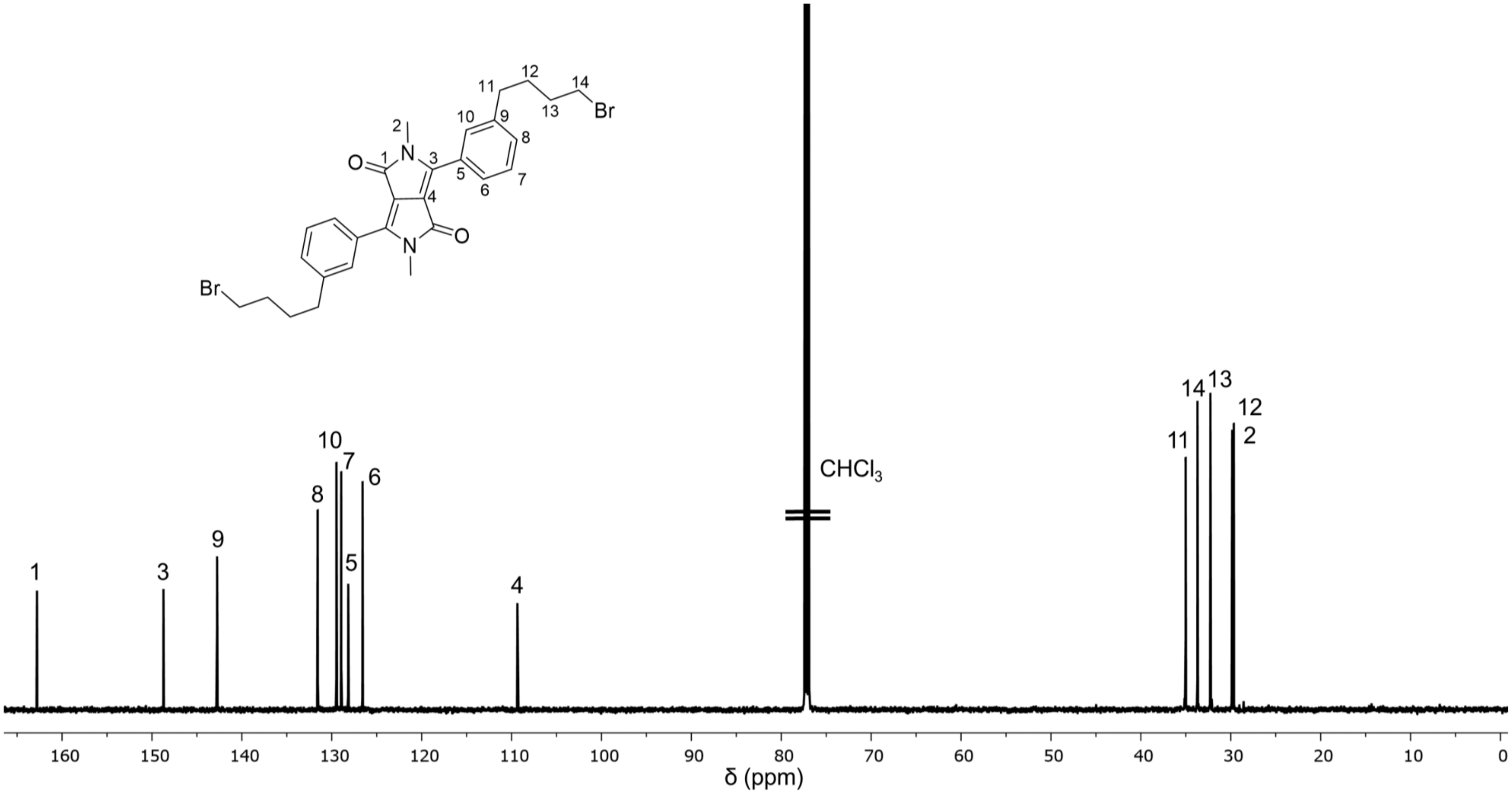

^{1}H NMR spectrum of **5a** in $CDCl_3$

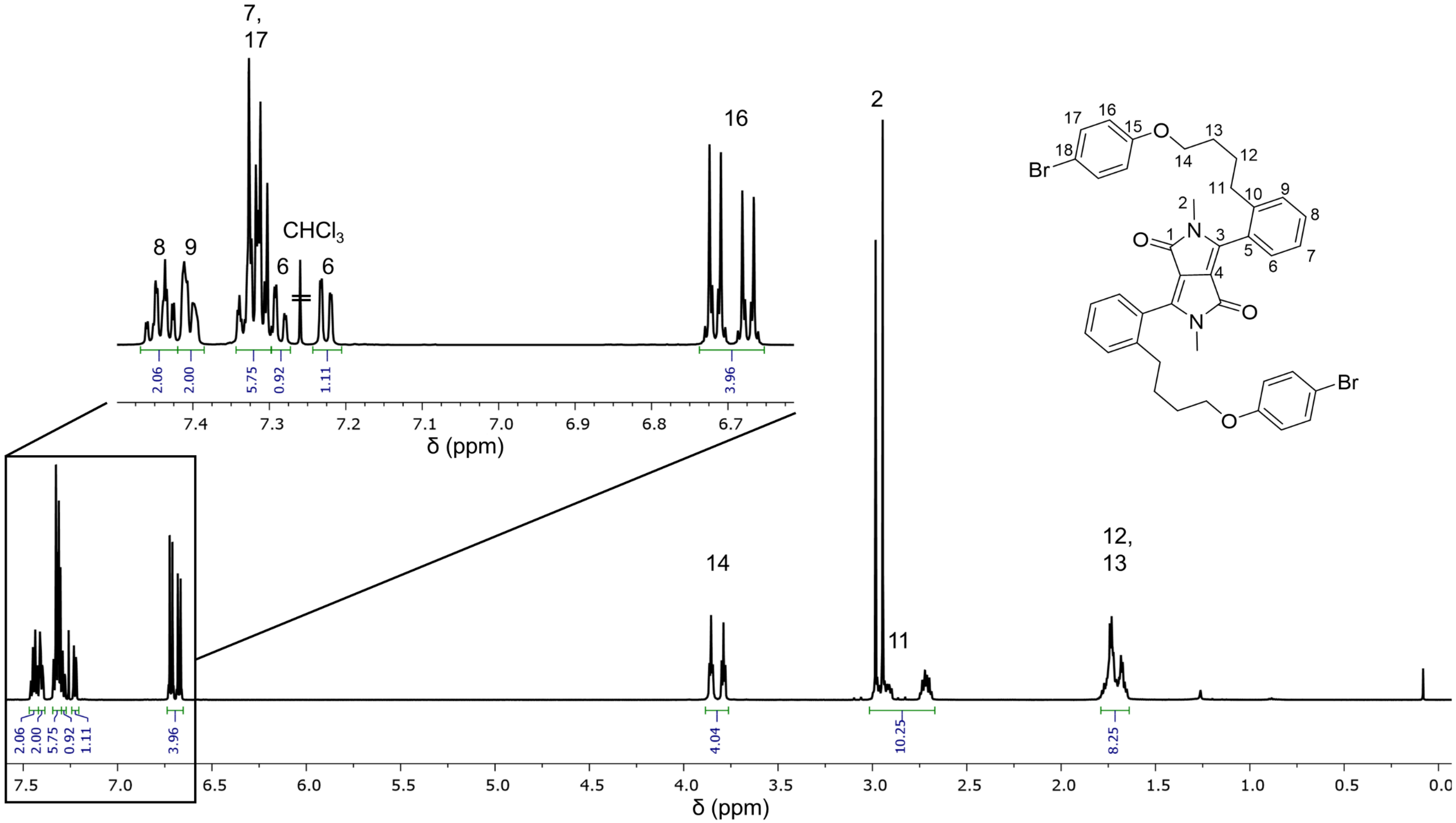

^{13}C NMR spectrum of **5a** in $CDCl_3$

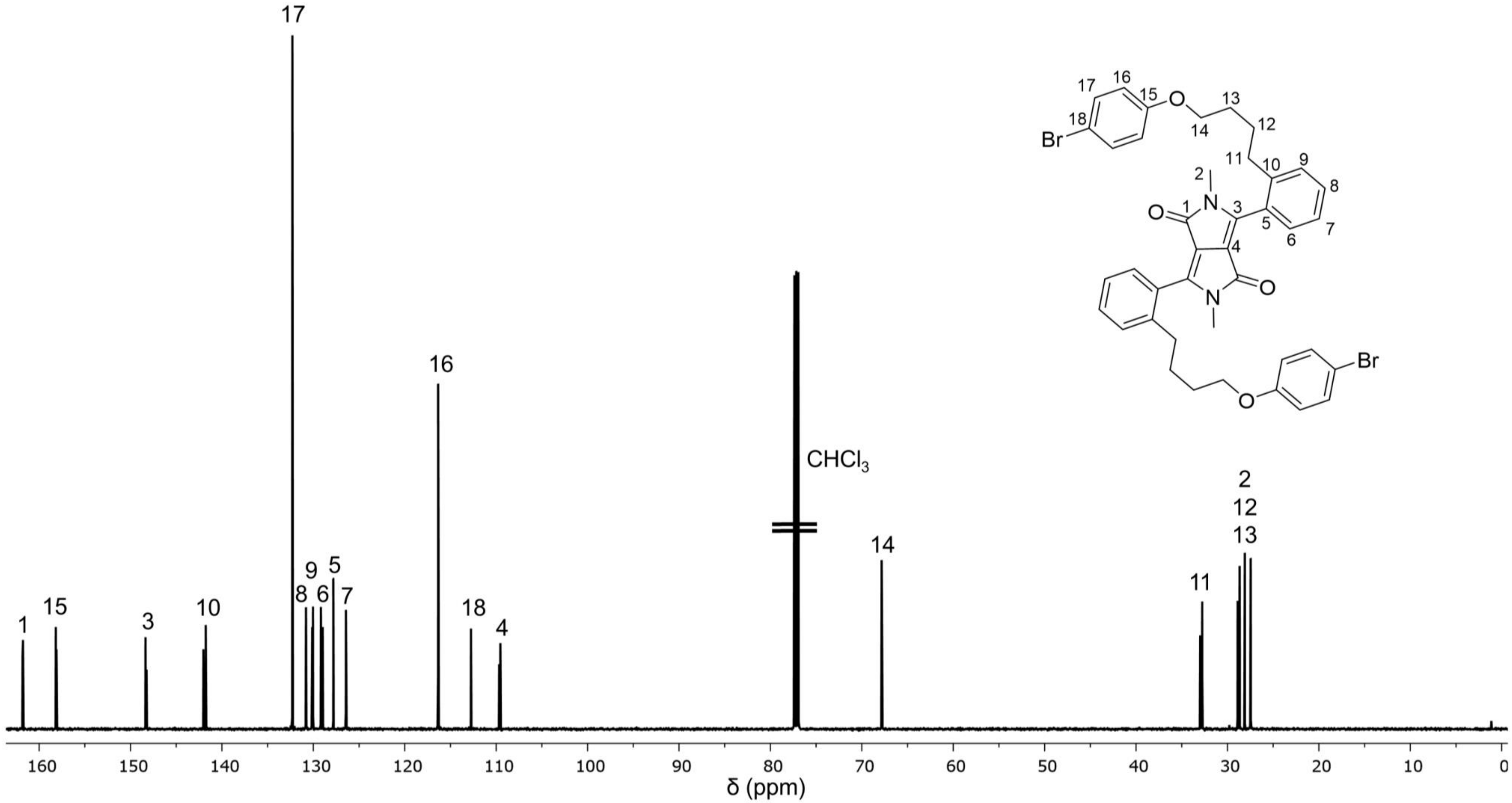

HSQC of **5a** in $CDCl_3$

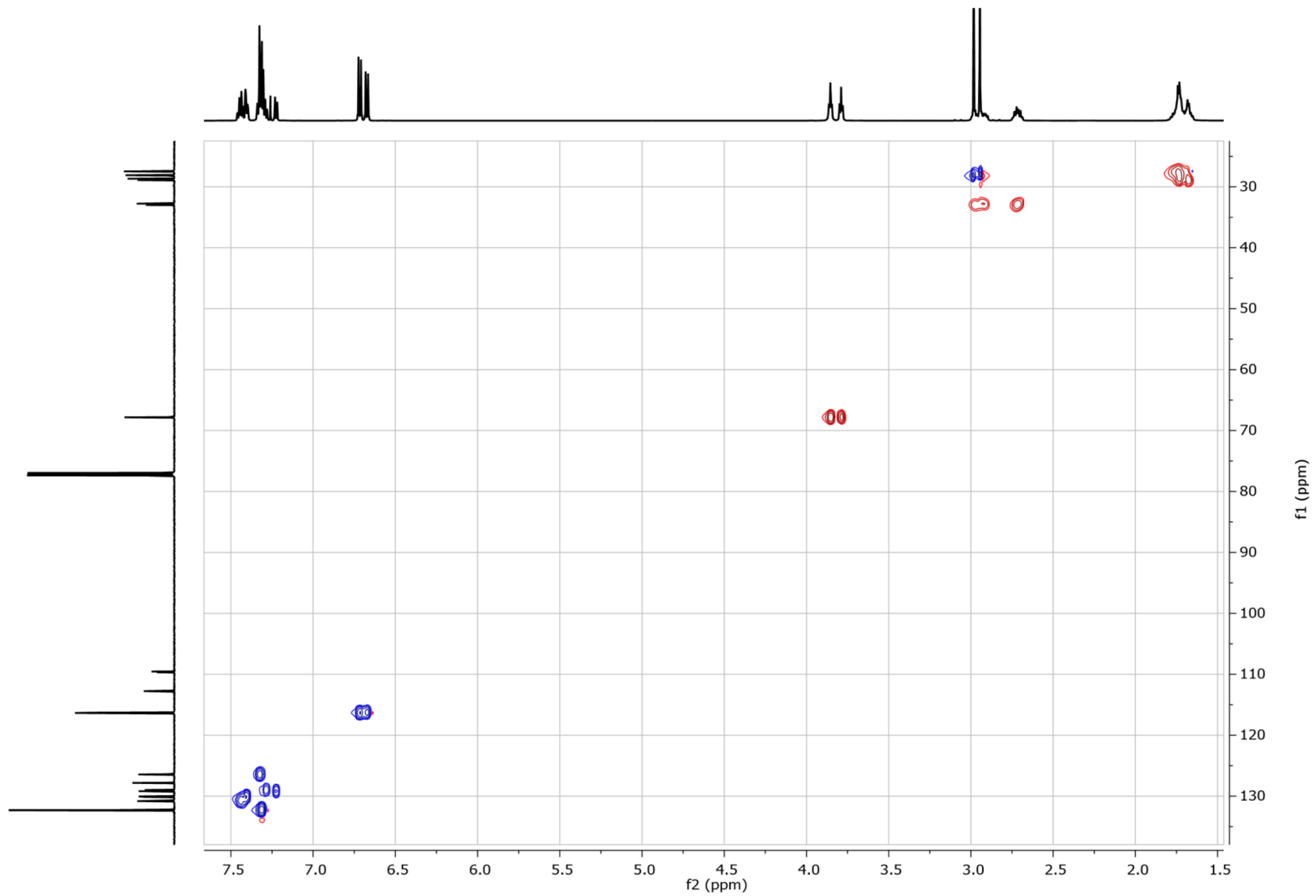

HMBC of **5a** in $CDCl_3$

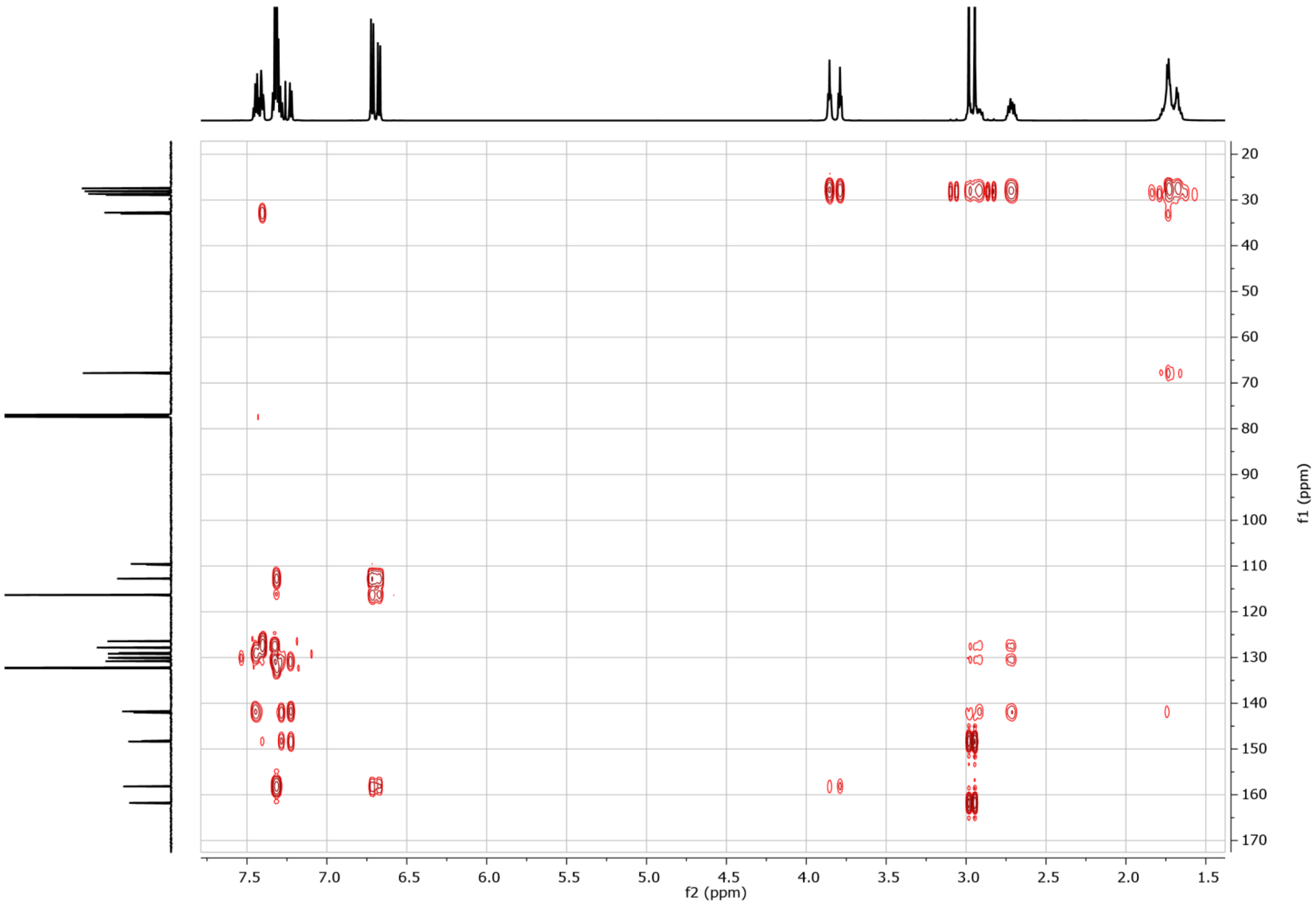

^1H NMR spectrum of **5b** in $CDCl_3$

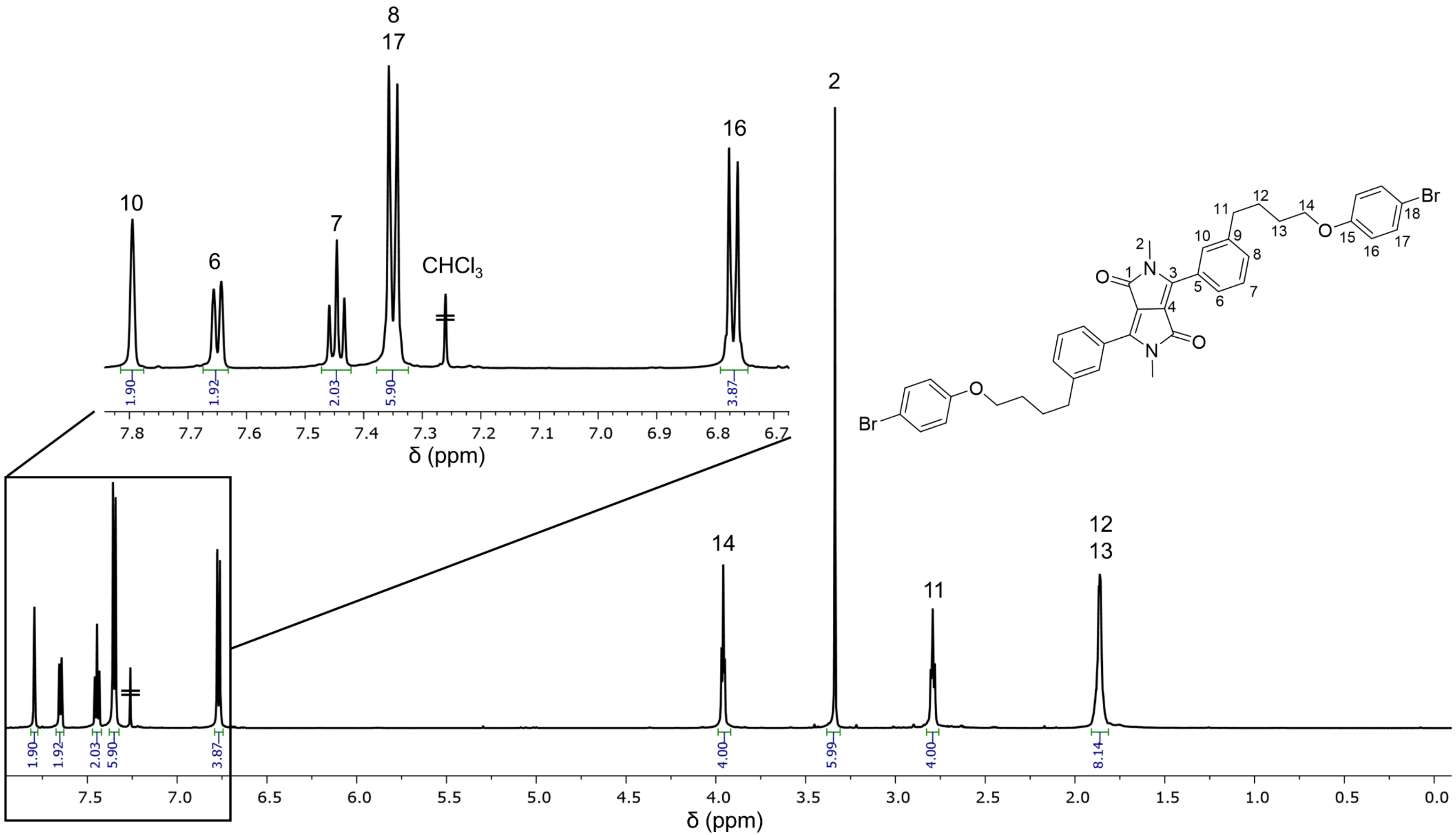

^{13}C NMR spectrum of **5b** in $CDCl_3$

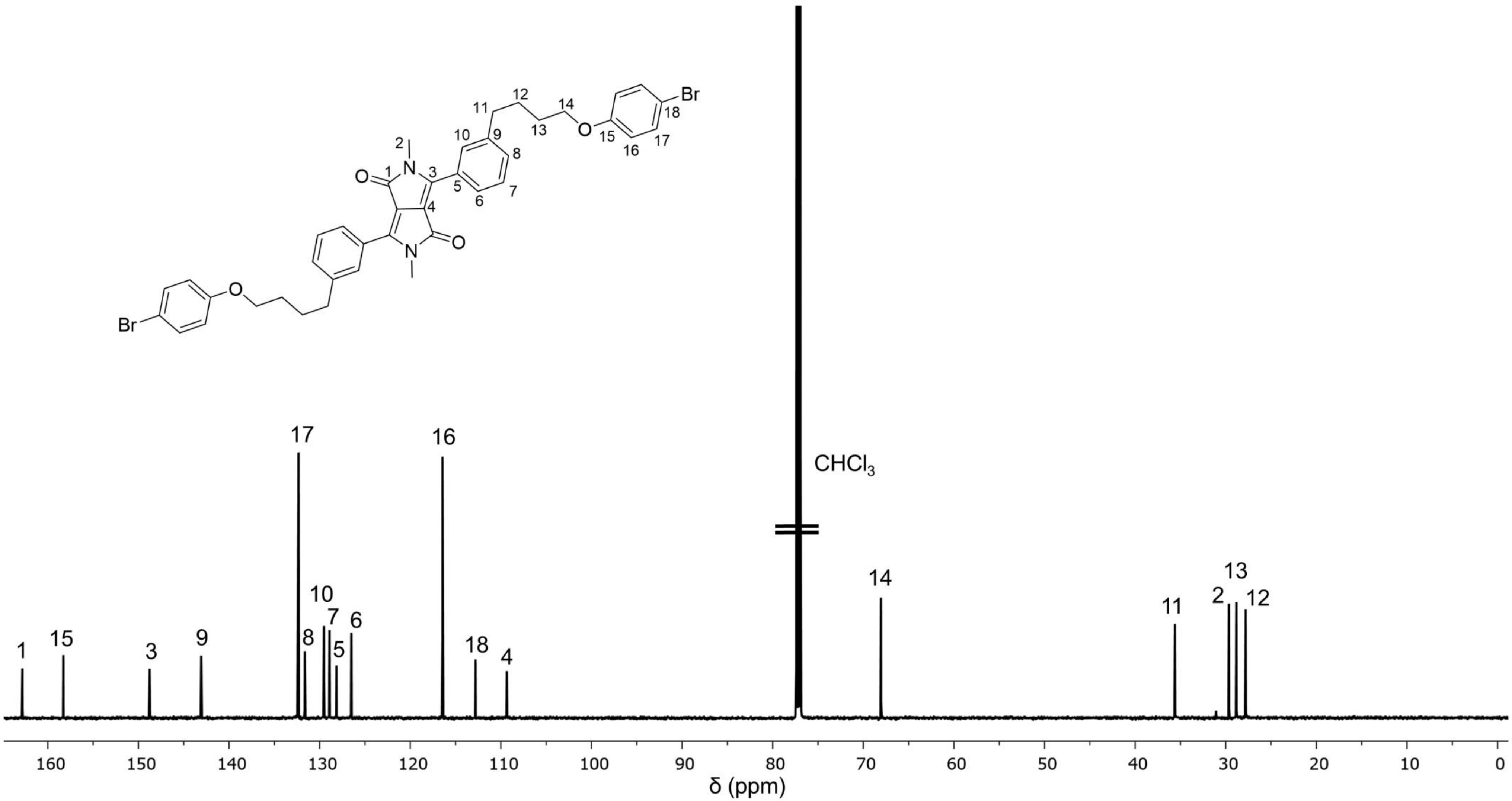

HSQC of **5b** in $CDCl_3$

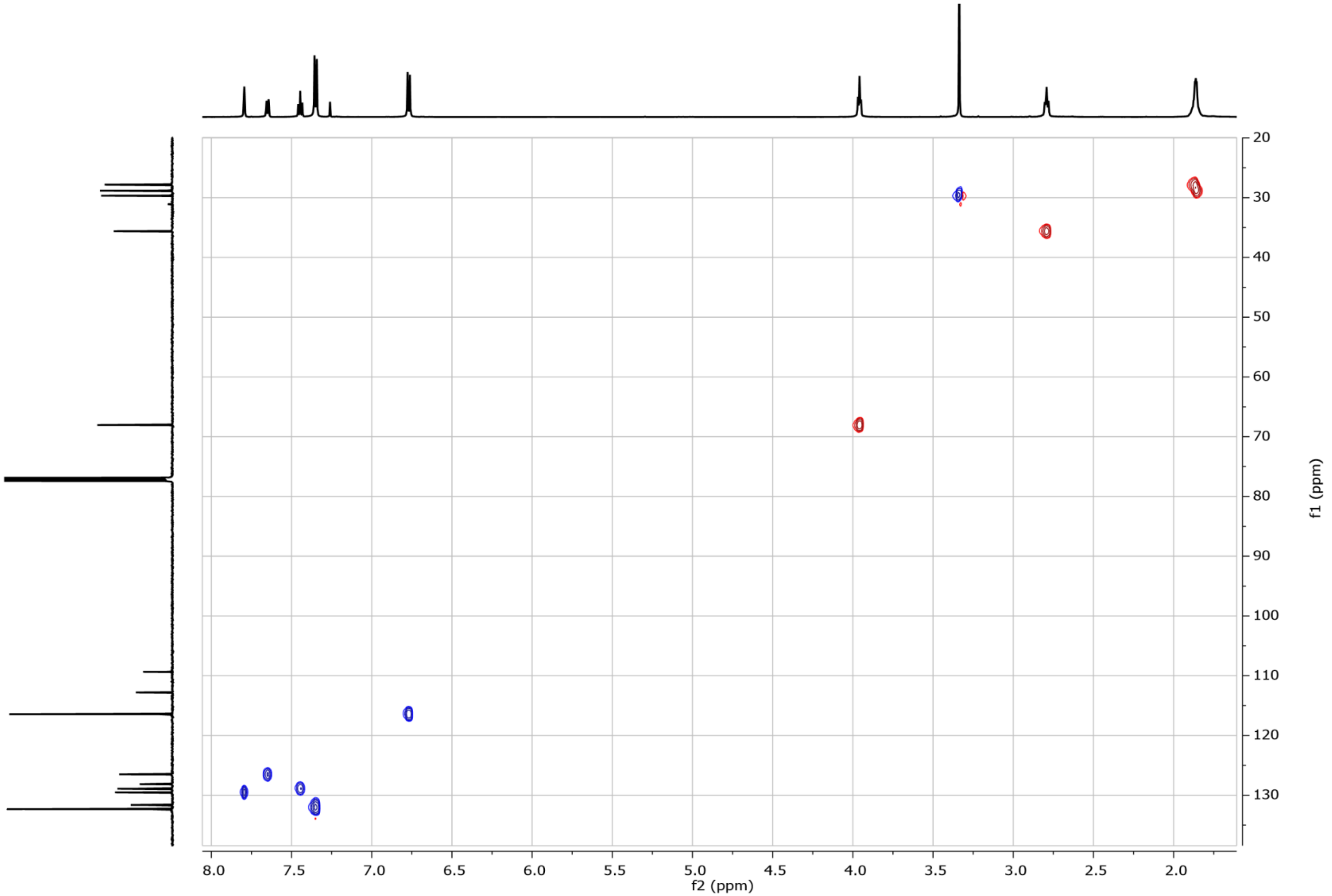

HMBC of **5b** in $CDCl_3$

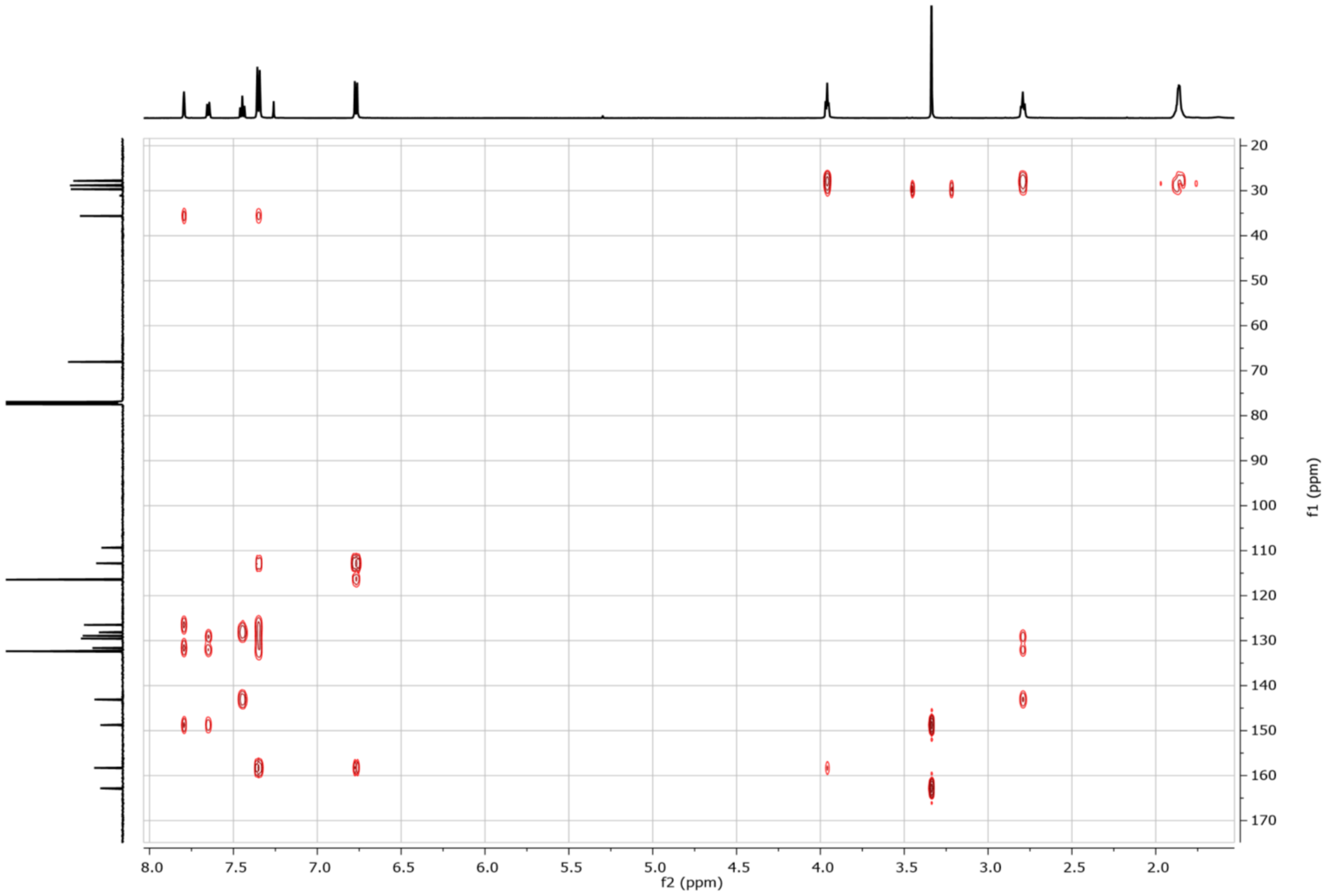

^{1}H NMR spectrum of **6a** in $CDCl_3$

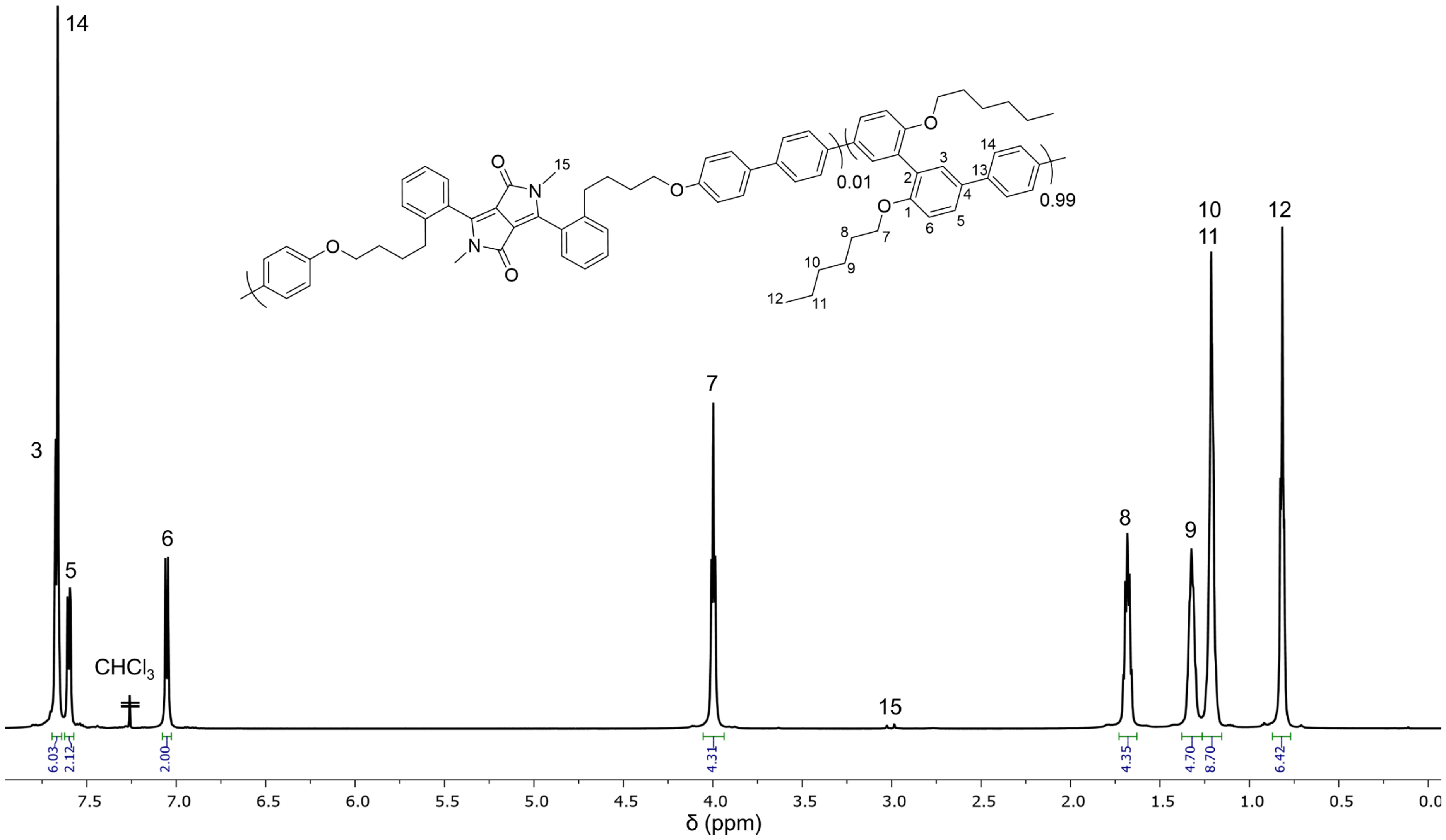

^{13}C NMR spectrum of **6a** in $CDCl_3$

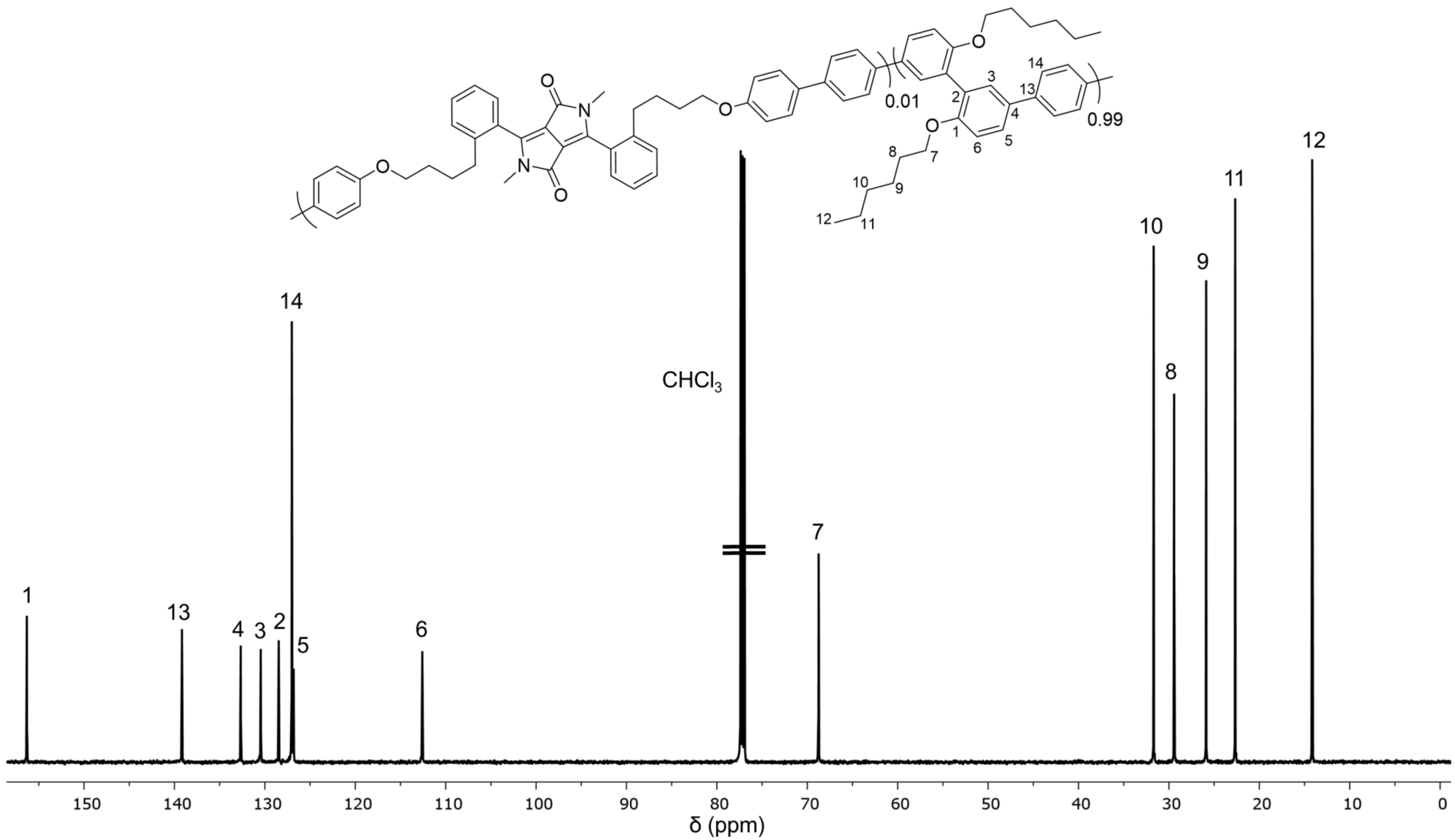

^1H NMR spectrum of **6b** in $CDCl_3$

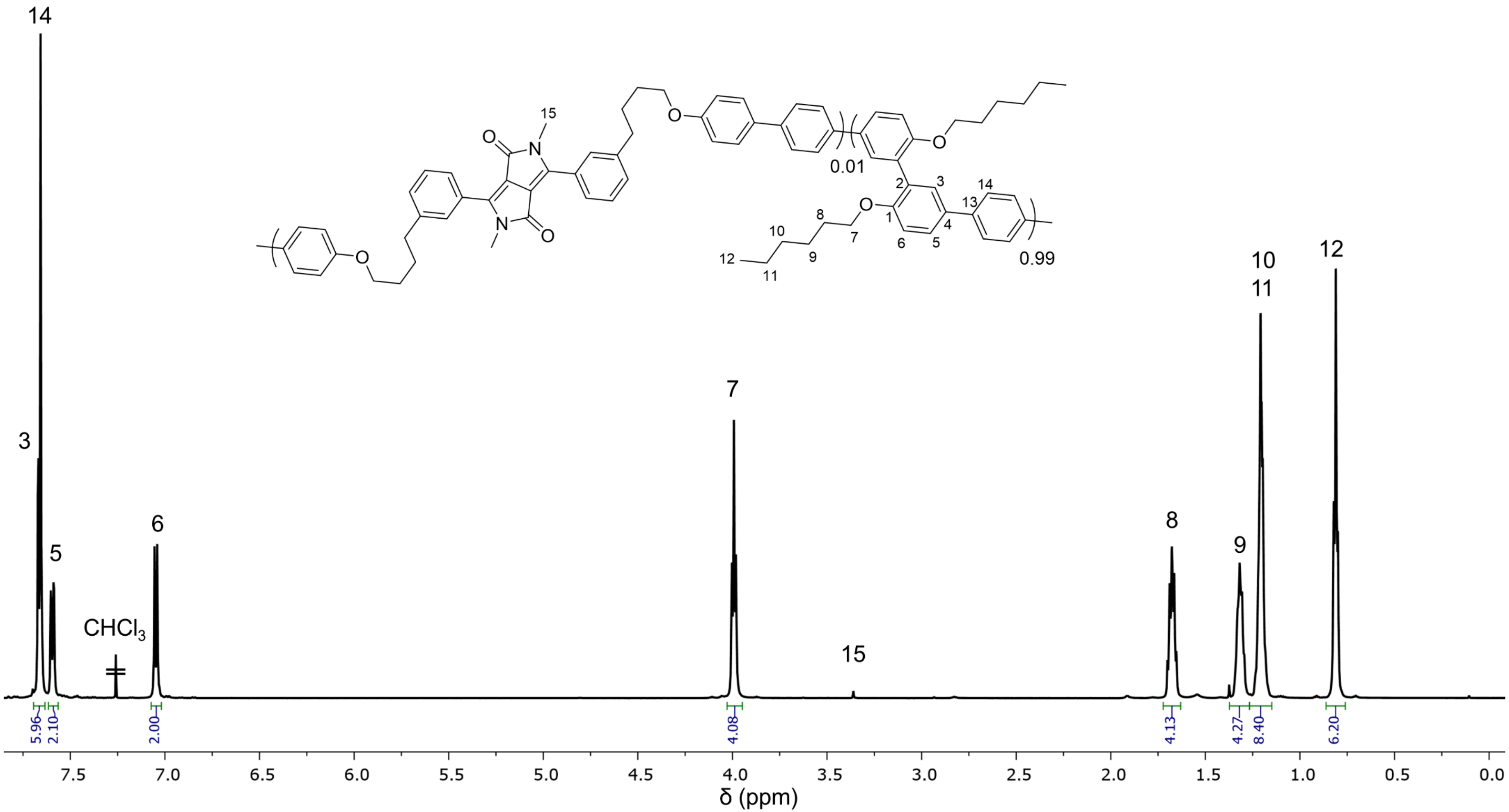

^{13}C NMR spectrum of **6b** in $CDCl_3$

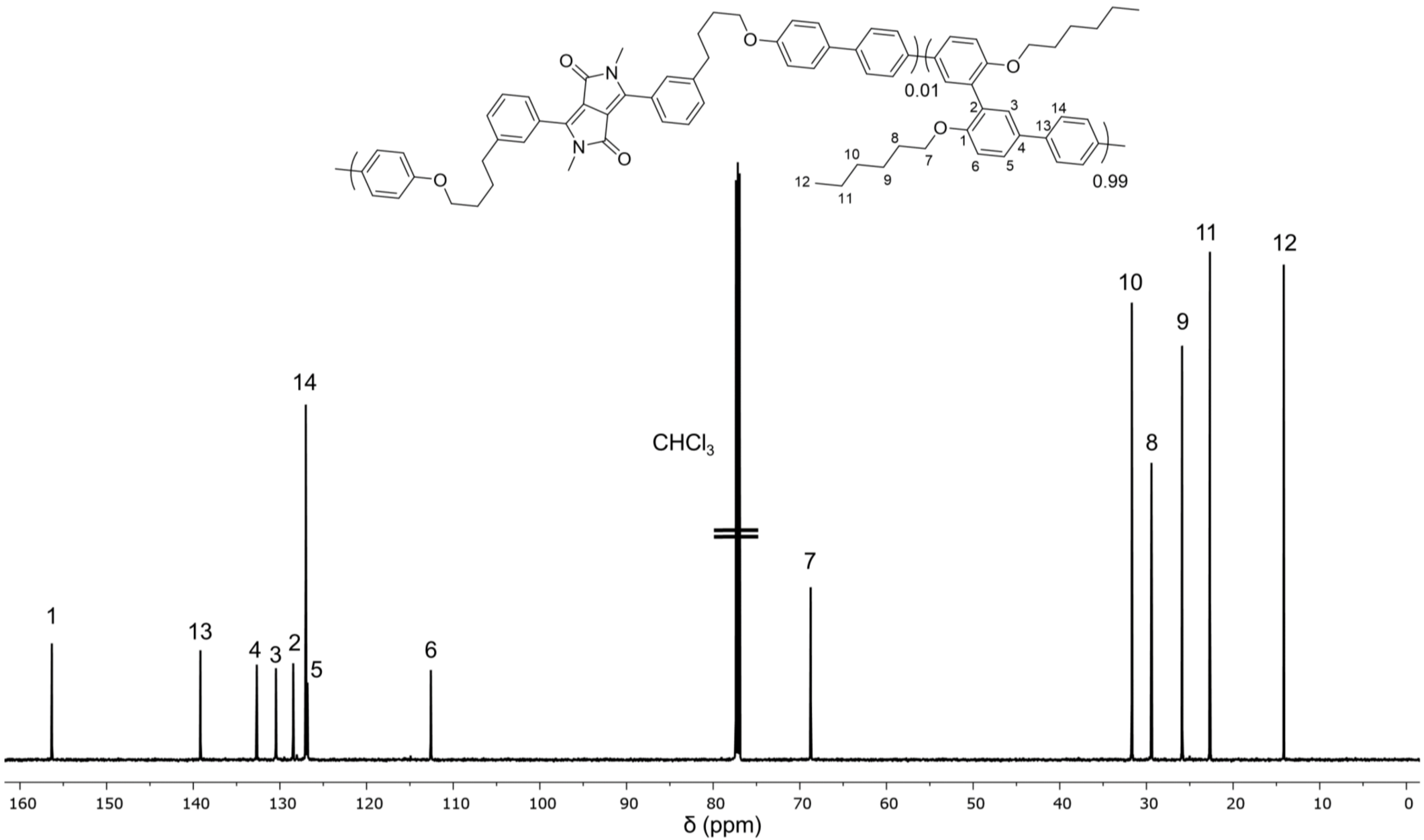

^{1}H NMR spectrum of **7** in $CDCl_3$

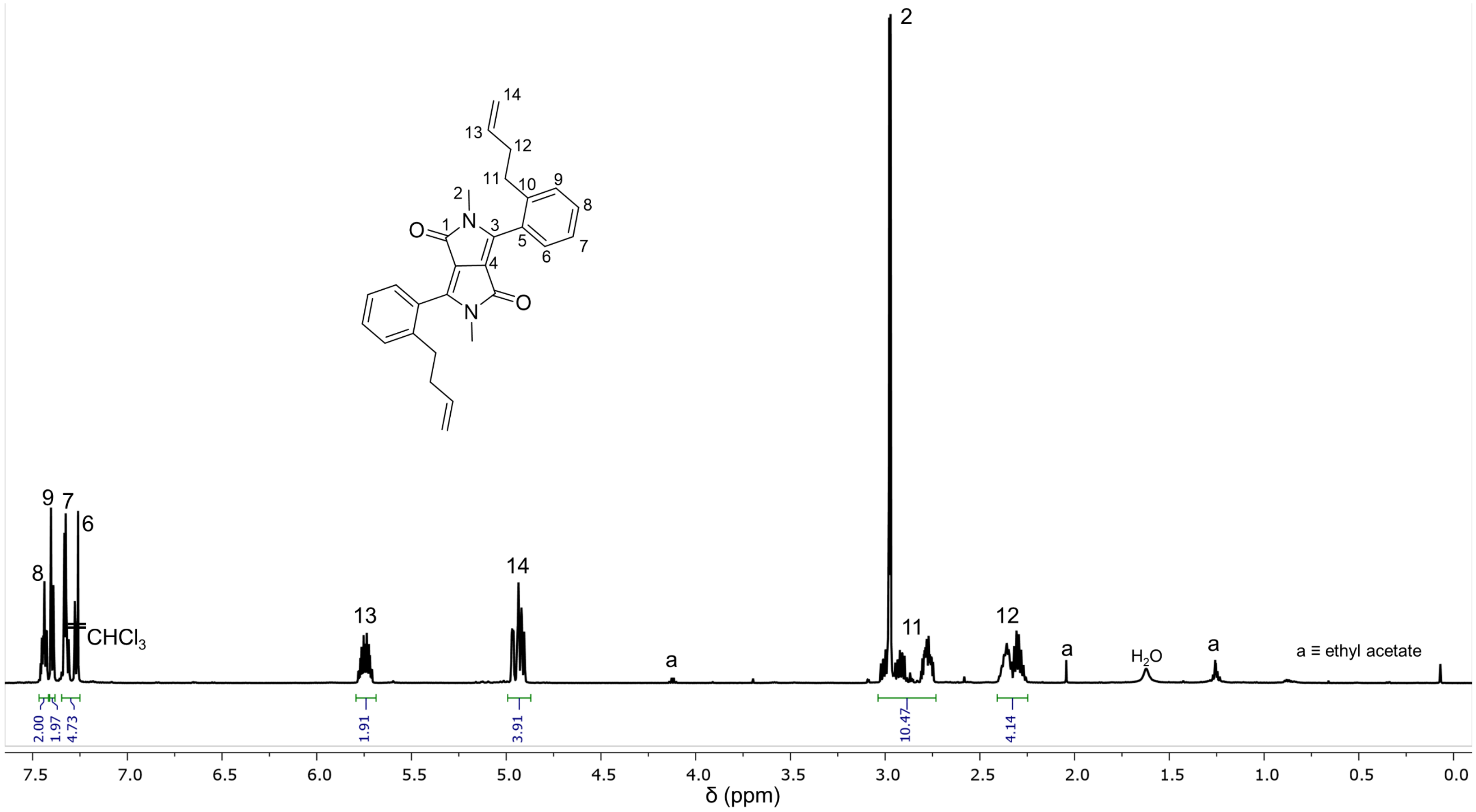

^{13}C NMR spectrum of **7** in $CDCl_3$

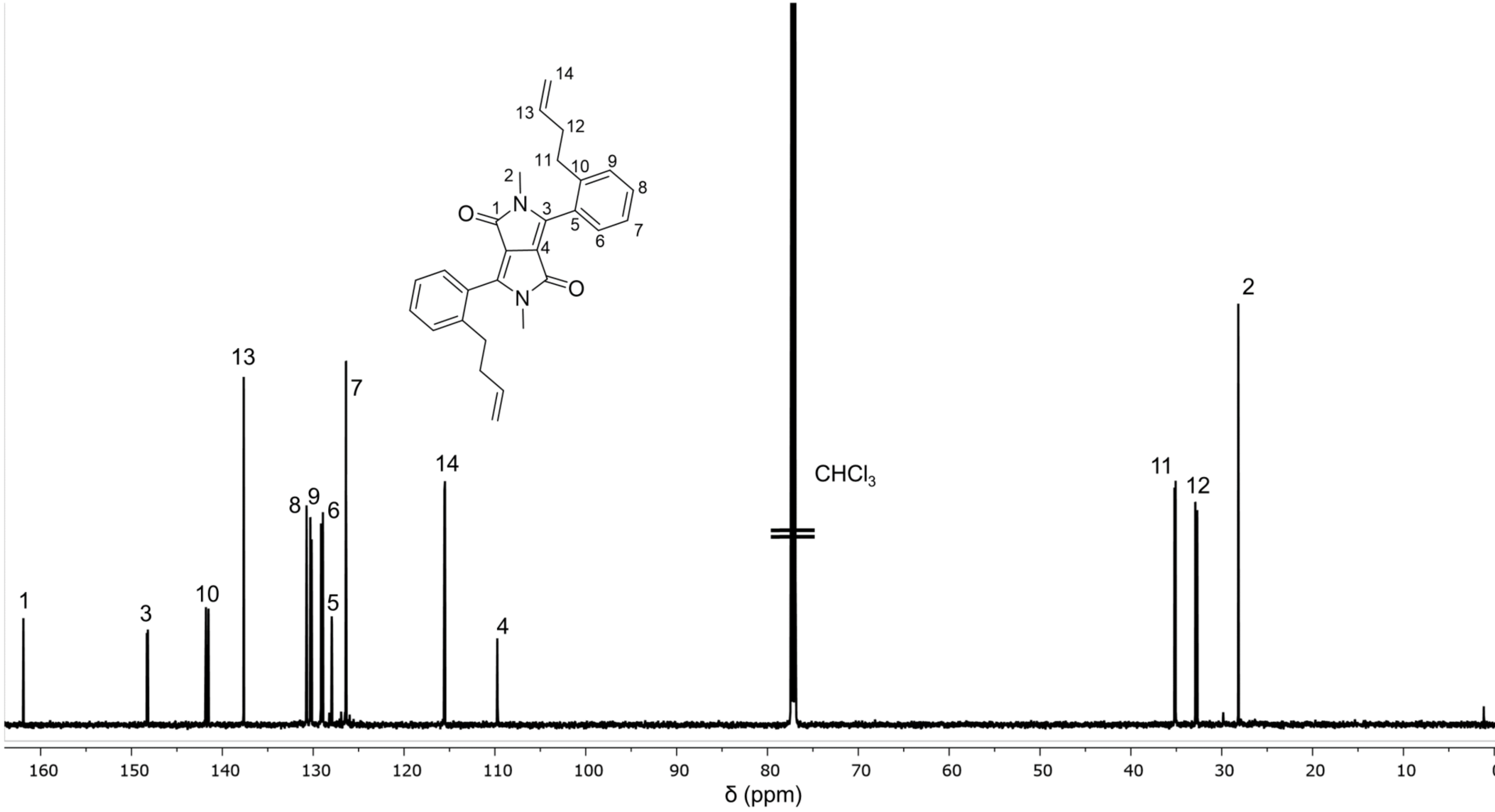

^{1}H NMR spectrum of **M1** in $CDCl_3$

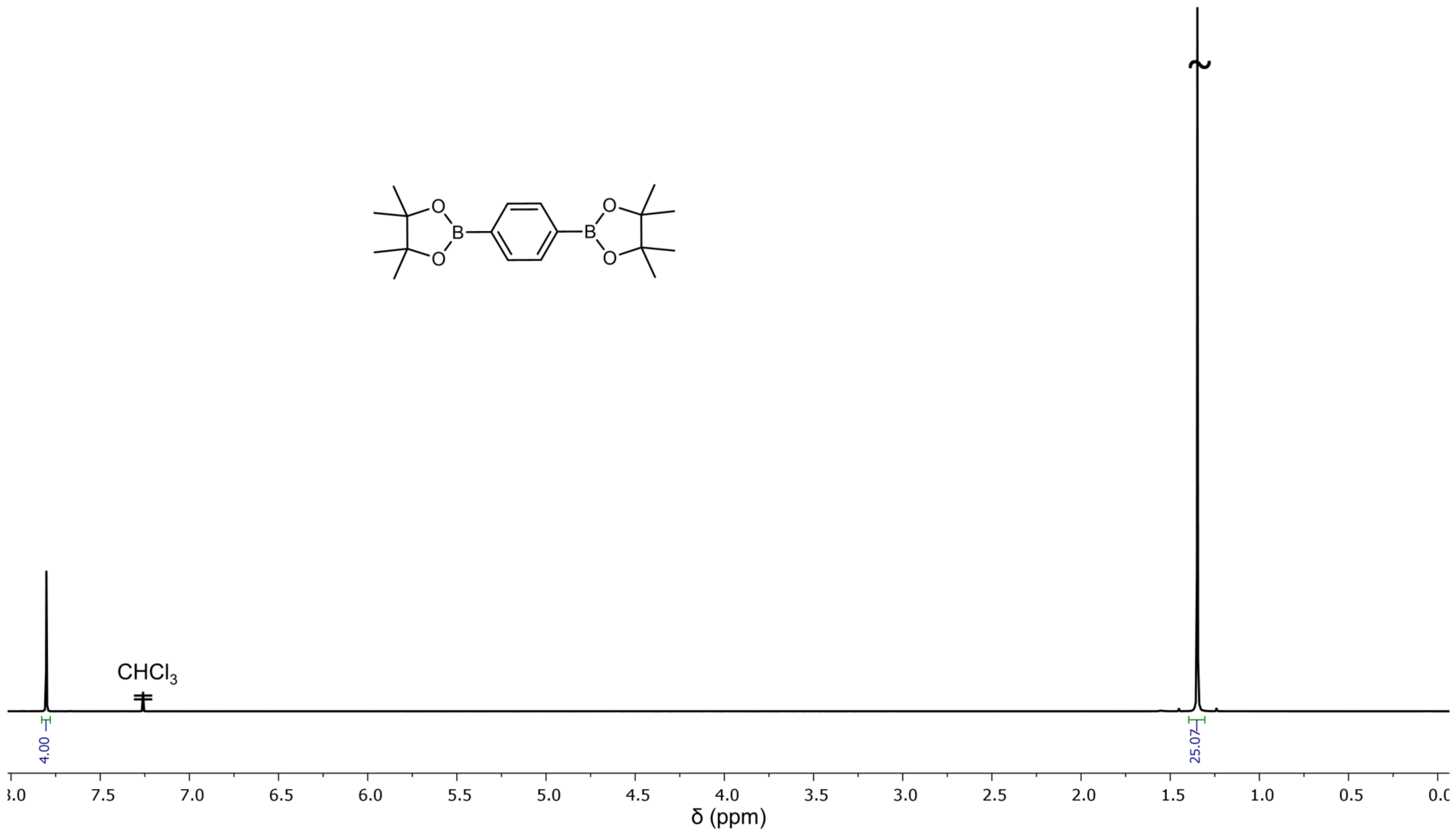

^{13}C NMR spectrum of **M1** in $CDCl_3$

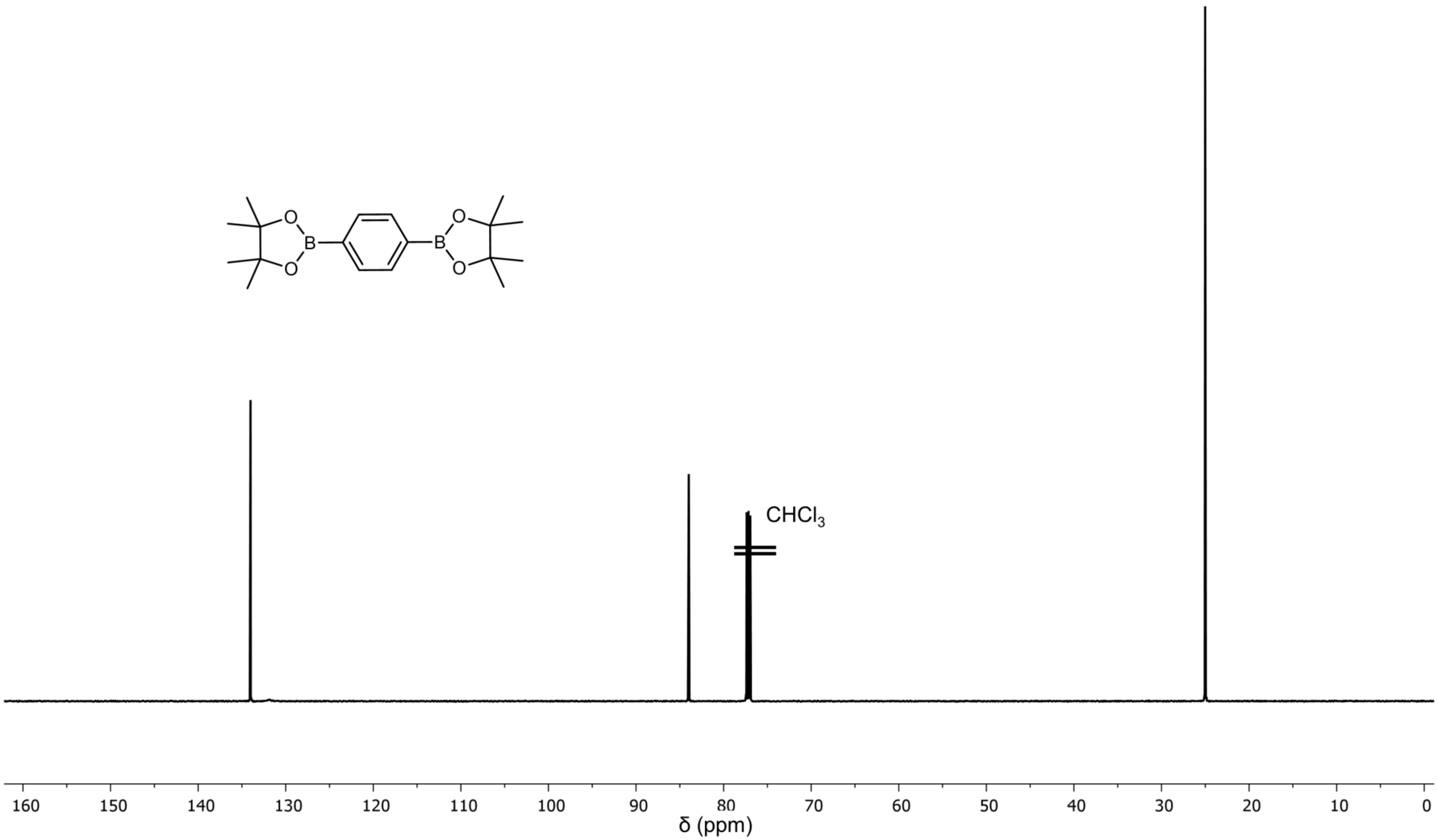

^{1}H NMR spectrum of **M2** in $CDCl_3$

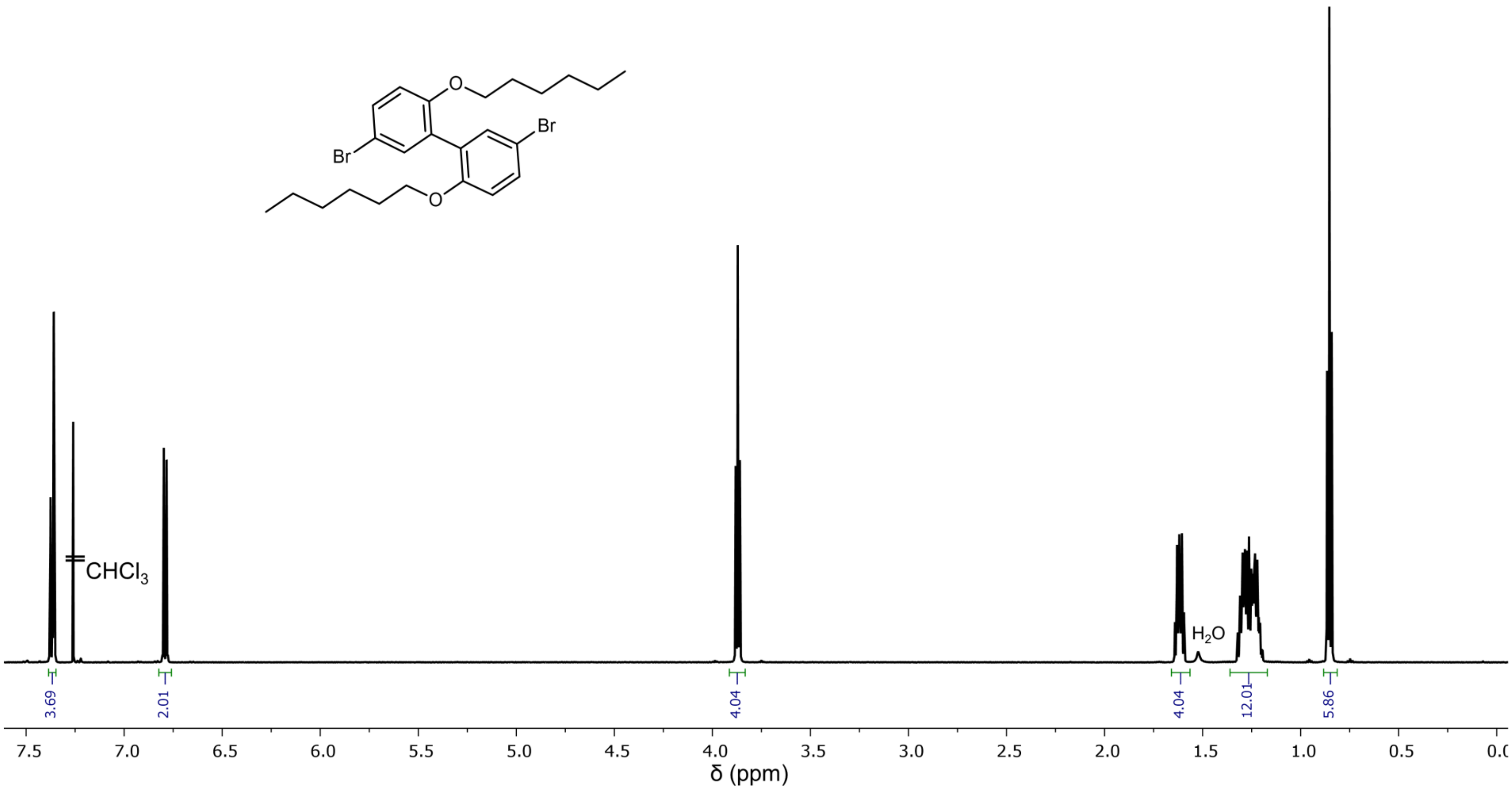

^{13}C NMR spectrum of **M2** in $CDCl_3$

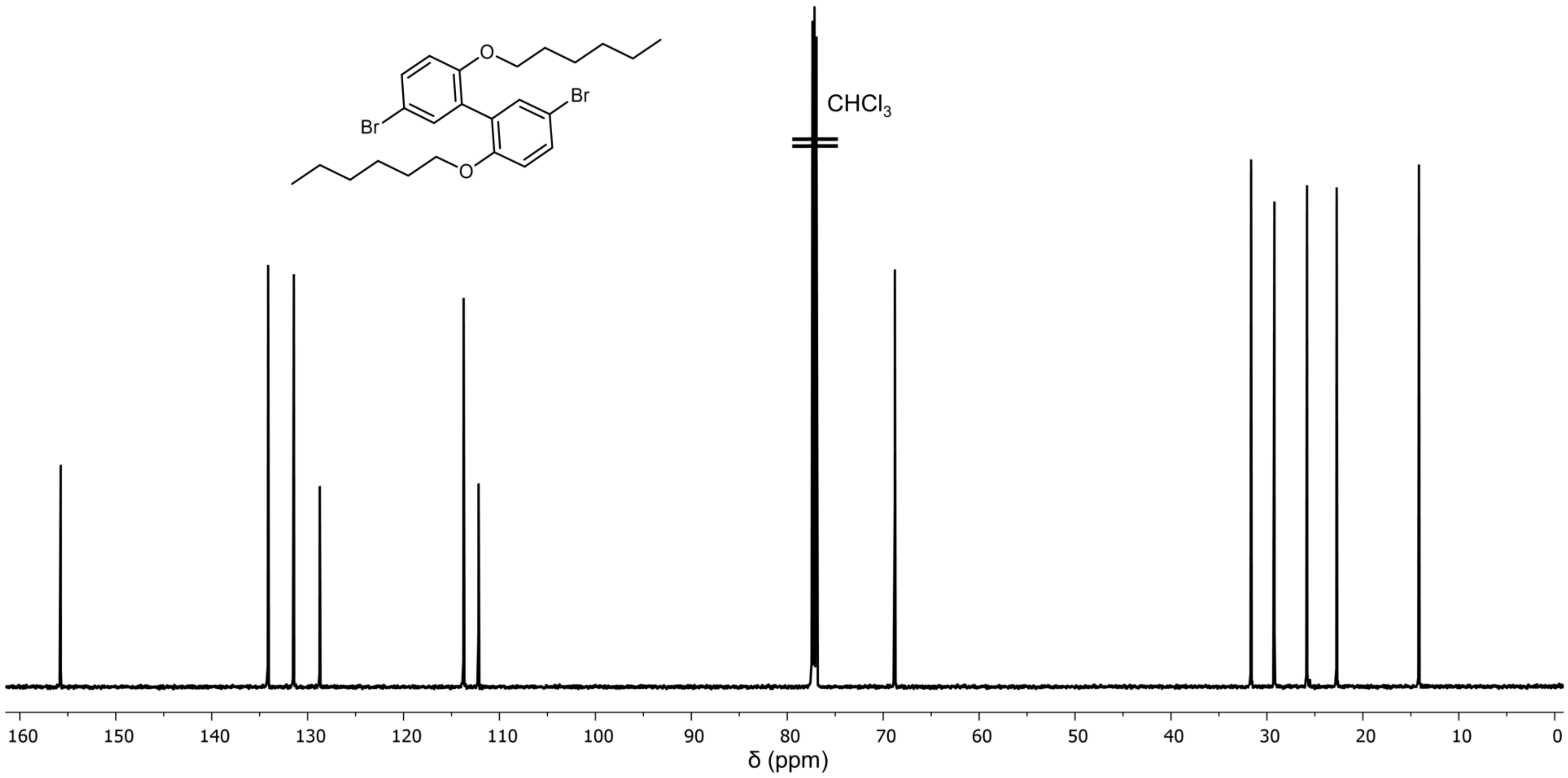

8. Supplementary references

1. Mortensen, J. J., Hansen, L. B. & Jacobsen, K. W. Real-space grid implementation of the projector augmented wave method. *Phys. Rev. B* **71**, 035109 (2005).
2. Enkovaara, J. *et al.* Electronic structure calculations with GPAW: a real-space implementation of the projector augmented-wave method. *J. Phys.: Condens. Matter* **22**, 253202 (2010).
3. Perdew, J. P., Burke, K. & Ernzerhof, M. Generalized Gradient Approximation Made Simple. *Phys. Rev. Lett.* **77**, 3865–3868 (1996).
4. Mark E. Casida. Time-Dependent Density Functional Response Theory for Molecules. in *Recent Advances in Density Functional Methods* vol. 1 155–192 (World Scientific, 1995).
5. Walter, M. *et al.* Time-dependent density-functional theory in the projector augmented-wave method. *The Journal of Chemical Physics* **128**, 244101 (2008).
6. Beyer, M. K. The mechanical strength of a covalent bond calculated by density functional theory. *The Journal of Chemical Physics* **112**, 7307–7312 (2000).
7. Ribas-Arino, J., Shiga, M. & Marx, D. Understanding Covalent Mechanochemistry. *Angewandte Chemie International Edition* **48**, 4190–4193 (2009).
8. Oliver Brügner & Michael Walter. Temperature and loading rate dependent rupture forces from universal paths in mechanochemistry. *Physical Review Materials* **2**, 113603 (2018).
9. Gossweiler, G. R. *et al.* Mechanochemical Activation of Covalent Bonds in Polymers with Full and Repeatable Macroscopic Shape Recovery. *ACS Macro Letters* **3**, 216–219 (2014).
10. Huo, S. Highly Efficient, General Procedure for the Preparation of Alkylzinc Reagents from Unactivated Alkyl Bromides and Chlorides. *Org. Lett.* **5**, 423–425 (2003).
11. Kempe, F. *et al.* A Simply Synthesized, Tough Polyarylene with Transient Mechanochromic Response. *Angewandte Chemie International Edition* **57**, 997–1000 (2018).
